# ﻿Systematic revision of species of *Atractilina* and *Spiropes* hyperparasitic on Meliolales (Ascomycota) in the tropics

**DOI:** 10.3897/mycokeys.103.115799

**Published:** 2024-04-11

**Authors:** Miguel A. Bermúdez-Cova, Tina A. Hofmann, Nourou S. Yorou, Meike Piepenbring

**Affiliations:** 1 Mycology Research Group, Faculty of Biological Sciences, Goethe University Frankfurt Am Main, Frankfurt Am Main, Germany Goethe University Frankfurt Am Main Frankfurt am Main Germany; 2 Departamento de Biología de Organismos, División de Ciencias Biológicas, Universidad Simón Bolívar, Caracas, Venezuela Universidad Simón Bolívar Caracas Venezuela; 3 Centro de Investigaciones Micológicas (CIMi), Herbario UCH, Universidad Autónoma de Chiriquí, David, Panama Universidad Autónoma de Chiriquí David Panama; 4 Research Unit Tropical Mycology and Plants-Soil Fungi Interactions (MyTIPS), Faculty of Agronomy, University of Parakou, BP 123, Parakou, Benin University of Parakou Parakou Benin

**Keywords:** Anamorph-teleomorph connection, Benin, Dothideomycetes, Hyperparasitism, Leotiomycetes, Panama

## Abstract

*Atractilina* Dearn. & Barthol. and *Spiropes* Cif. are genera of asexual fungi that comprise species mainly hyperparasitic on black mildews (Meliolales, Ascomycota). Although a common group of anamorphic fungi, they have been described up to now only by morphology and their systematic position is unknown. The present study provides a morphological treatise of all known species of *Atractilina* and *Spiropes* hyperparasitic on Meliolales, including insights into their systematic position, based on DNA sequences generated here for the first time. The study was conducted, based on 33 herbarium specimens and 23 specimens recently collected in Benin and Panama. The obtained DNA sequence data (28S rDNA and ITS rDNA) of *A.parasitica* and of two species of *Spiropes* show systematic placements in the Dothideomycetes and Leotiomycetes, respectively. The sequence data of the two *Spiropes* spp. do not group together. Moreover, the anamorph-teleomorph connection between *Atractilinaparasitica* and *Malacariameliolicola*, a pseudothecioid fungus, is confirmed. Three species in the genus *Spiropes* are proposed as new to science, namely *S.angylocalycis*, *S.carpolobiae* and *S.croissantiformis*. Four species are reported for Benin for the first time, three species for Panama and one species for mainland America. *Atractilina* and *Spiropes* are currently two genera with highly heterogeneous species and they might have to be split in the future, once the taxonomic concepts are validated by morphology and molecular sequence data.

## ﻿Introduction

Meliolales (Sordariomycetes, Ascomycota) form a large order of biotrophic, obligate plant parasitic fungi in the Tropics and subtropics (Piepenbring et al. 2011; Hongsanan et al. 2015; Zeng et al. 2017). The order comprises two families, Armatellaceae and Meliolaceae, with *Armatella* Theiss. & Syd. and *Meliola* Fr. being the most species-rich genera of each family, respectively (Hosagoudar 2003; Jayawardena et al. 2020). They are commonly known as “black mildews”, because they produce black colonies that are composed of dark, thick-walled, branched, superficial hyphae (Rodríguez Justavino et al. 2015).

Approximately 200 species of hyperparasitic fungi, i.e. fungi parasitic on other parasites, have been reported to grow on colonies of Meliolales (Bermúdez-Cova et al. 2022, 2023a). These hyperparasites mainly belong to the Dothideomycetes and the Sordariomycetes, although the systematic positions of a large number of these fungi still remain unknown (Bermúdez-Cova et al. 2022; Bermúdez-Cova et al. 2023a). Hyperparasitic fungi frequently overgrow entire colonies of black mildews, so the meliolalean host may be detected only by careful search with a light microscope (Stevens 1918; Ciferri 1955; Bermúdez-Cova et al. 2023a).

Amongst the hyperparasitic fungi, species of the anamorphic genera *Atractilina* Dearn. & Barthol. and *Spiropes* Cif. are common hyperparasites of black mildews in the tropics. In the past, they were regarded as conidial stages of Meliolales (Ciferri 1955; Bermúdez-Cova et al. 2023b) and nowadays as incertae sedis in the Ascomycota (Bermúdez-Cova et al. 2022). The genus *Atractilina* includes six species of mostly hyperparasitic hyphomycetes with true synnemata, denticulate conidiogenous loci and pale pluriseptate conidia (Deighton and Pirozynski 1972; Mel’nik and Braun 2013). On the other hand, the genus *Spiropes* comprises 34 species of dematiaceous, mostly hyperparasitic hyphomycetes with mononematous, fasciculate or synnematous conidiophores (Ellis 1968, 1971, 1976; Seifert and Hughes 2000; Bánki et al. 2023). Species of *Spiropes* are characterised by the presence of conidiogenous cells with conspicuous, flat and numerous scars, as well as pigmented conidia with 1–9 septa or pseudosepta (Ellis 1968).

*Arthrobotryum* Ces., *Cercospora* Fresen. ex Fuckel, *Helminthosporium* Link, *Pleurophragmium* Costantin and *Podosporium* Schwein. are only a few of the many genera to which species of *Atractilina* and *Spiropes* have been assigned in the past, although they were not congeneric with the type specimens of those genera (Ellis 1968; Deighton and Pirozynski 1972; Alcorn 1988). This resulted in taxonomic uncertainty with species being transferred from one genus to another. This problem was initially addressed by Ellis (1968) and Deighton and Pirozynski (1972), as they did an extensive morphological revision of taxa now assigned to *Atractilina* or *Spiropes*. For example, all the synnematous fungi, hyperparasitic on Meliolales formerly assigned to the genus *Arthrobotryum*, were transferred to the genus *Spiropes* by Ellis (1968), with the exception of *A.parasiticum* (Winter) Hansf., which was transferred to the genus *Atractilina* by Deighton and Pirozynski (1972).

There is currently one valid species of *Atractilina*, namely *A.parasitica* (G. Winter) Deighton & Piroz. and 19 species of the genus *Spiropes* known to be hyperparasitic on colonies of Meliolales (Ellis 1968; Deighton and Pirozynski 1972; Mel’nik and Braun 2013; Bermúdez-Cova et al. 2022). However, species delimitation within these two genera has up to now been done by morphology only, as species were described in the past before the molecular era and because of the challenges of isolating DNA from mixed infections (Bermúdez-Cova et al. 2022, 2023a, 2023b). As a result, the systematic position of both genera within the Ascomycota remained unknown. The present study revises the morphology of the species of *Atractilina* and *Spiropes* and provides the first insights into their systematic position according to molecular sequence data, with emphasis on the species hyperparasitic on Meliolales.

## ﻿Materials and methods

### ﻿Sample collection and morphological characterisation

Samples of leaves infected with black mildews were opportunistically collected in western Panama from January-March 2020 and in Benin in February as well as September-October 2022. For the present study, colonies of Meliolales hyperparasitised by *Atractilinaparasitica* and species of *Spiropes* were considered. Infected leaves were dried in a plant press and deposited in the Herbarium at the Universidad Autónoma de Chiriquí (UCH, specimens from Panama) or in the Mycological Herbarium of the University of Parakou (UNIPAR) in Benin. Duplicates of large-sized samples were deposited in the Botanische Staatssammlung München (M). In some cases, fungal tissue was collected prior to drying of the specimens and preserved in CTAB buffer for subsequent DNA extraction.

Dried specimens were observed by stereomicroscopy and by light microscopy (LM). Measurements of at least 20 conidia and other structures have been made for each specimen at magnifications of 600× and 1000×. Measurements are presented as mean value ± standard deviation with extreme values in parentheses. Line drawings were made freehand on scaled paper. Scars on conidiophores are drawn in surface view although further cells of the conidiophore are drawn in optical sections. Images and drawings were edited with Photoshop (Adobe, San Jose, California). Specimens were also analysed morphologically by scanning electron microscopy (SEM). Materials used for SEM were prepared according to Hofmann et al. (2010).

### ﻿Host plant identification

Host plants were identified by morphological characteristics and, in some cases, by molecular sequence data. Morphological identifications were made by comparison with herbarium specimens, literature (e.g. Akoègninou et al. (2006); Condit et al. (2011)) and with the help of local botanists. Molecular sequence data for species identifications were obtained by polymerase chain reaction (PCR) for the amplification of the partial region of chloroplast rbcL with the primer pairs rbcLa-F (Levin et al. 2003) and rbcLa-R (Kress et al. 2009). DNA was extracted from approx. 0.05 g of leaf tissue dried with silica gel using the innuPREP Plant DNA Kit (Analytik Jena, Germany) and following the manufacturer’s instructions. Protocols for PCR were carried out as described by Fazekas et al. (2012).

### ﻿DNA extraction, PCR amplification and sequencing of fungal DNA

DNA was isolated from the synnemata and hyphae of specimens using the E.Z.N.A Forensic DNA Extraction Kit, following the manufacturer’s instructions. To extract total genomic DNA, a small amount of clean synnemata or single conidiophores were transferred into a sterile Eppendorf tube with approx. 200 μl of distilled water using sterilised tweezers and trying to avoid picking cells of any other organism associated with the leaves and the colonies of black mildews. For example, for the synnemata of *Atractilinaparasitica* and *Spiropesmelanoplaca*, only the upper parts were used for DNA extraction, in order to avoid the basal parts that are in direct contact with cells of other organisms. The samples were frozen for 24 h at -20 °C, and later homogenised for 10–12 min. using a Retsch Mixer Mill MM301 with TL buffer and 2.5 mm Zirconia beads. Isolated DNA was re-suspended in elution buffer and stored at -20 °C.

Two partial nuclear gene regions (ribosomal loci) were amplified and sequenced: For the large subunit nuclear ribosomal DNA (nrLSU, 28S rDNA), the primers LR0R (Wagner and Ryvarden 2002) and LR5 (Vilgalys and Hester 1990) were used. For the internal transcribed spacer region of ribosomal DNA (ITS), the primers ITS5 and ITS4 (White et al. 1990) were used. The PCR mixtures consisted of 1 μl genomic DNA, 15× MgCl_2_ reaction buffer (Bioline, Luckenwalde, Germany), 25 mM MgCl_2_, 25 μM of each dNTP, 10 μM of each primer and 5 U Taq DNA polymerase (VWR) in a total volume of 30 μl. Cycling parameters of the PCR were as follows: initial denaturation at 94 °C for 3 min, followed by 35 cycles of amplification [denaturation at 94 °C for 30 s, primer annealing at 52 °C for 30 s and primer extension at 72 °C for 45 s] and a final extension at 72 °C for 5 min, followed by storage at 8 °C. PCR-products were checked on 1.5% agarose electrophoresis gels containing HDGreenPlus DNA stain. Amplified PCR products were purified with the Cycle Pure Kit (VWR-Omega, USA). Sequencing was performed at Seqlab GmbH, Germany.

### ﻿Phylogenetic analyses

Consensus sequences of trace files were generated with Geneious 10.2.2 (https://www.geneious.com, Kearse et al. 2012) and searched against GenBank (https://www.ncbi.nlm.nih.gov/, Benson et al. 2014) with MegaBLAST. Ambiguous and miscalled bases were corrected, when possible, after examination of the corresponding chromatogram files. Sequences with a high similarity were aligned with MAFFT v. 7 using the L-INS-i algorithm (Nakamura et al. 2018). The alignments were manually checked by using MEGA v. 7 (Kumar et al. 2016). Gblocks v. 0.91b (Talavera and Castresana 2007) was used to remove poorly-aligned positions and divergent regions from the DNA alignment. Phylogenetic analyses of this study were conducted by applying Maximum Likelihood (ML) in RAxML-HPC2 v.8.2.12 (Stamatakis 2014) on XSEDE (Miller et al. 2010) and Bayesian phylogenetic inference with the programme MrBayes 3.2.6. (Ronquist et al. 2012) on XSEDE (Miller et al. 2010), available on the CIPRES Science Gateway web portal (http://www.phylo.org/sub_sections/portal/). The alignment and tree are included in Suppl. material 1.

We also used T-BAS 2.1 (Carbone et al. 2019) and the “Place Unknowns” tool to place newly-generated ITS sequences on to the Pezizomycotina tree version 2. Two FASTA files of the newly-generated ITS sequences of *Spiropes* were uploaded to the T-BAS interface. We selected the “de novo” option for the RAxML placement, with 500 bootstrap replicates.

## ﻿Results

### ﻿Taxonomy

Based on morphological evidence, the hyperparasitic fungi collected in Panama and Benin are assigned to the genera *Atractilina* or *Spiropes*. Amongst these, three species are proposed as new to science, all in the genus *Spiropes*. Four species represent new reports for Benin and three for Panama. We also present a revision from herbarium material of 17 of the 19 known species of the genus *Spiropes* and one species of *Atractilina* hyperparasitic on Meliolales. All species synonyms, unless specified, are taken from Deighton and Pirozynski (1972) for *Atractilinaparasitica* and from Ellis (1968) for species of *Spiropes*.

#### ﻿*Atractilina* Dearn. & Barthol., Mycologia 16: 175, 1924.

##### ﻿ Atractilina parasitica

Taxon classificationFungiMeliolalesAscomycota

(G. Winter) Deighton & Piroz., Mycol. Pap. 128: 34, 1972

CCE64AAC-C623-5E27-829A-E3902FEADE7D

Fig. 1

≡ Arthrosporiumparasiticum G. Winter, Hedwigia 25: 103, 1886.
≡ Arthrobotryumparasiticum (G. Winter) Hansf., Proc. Linn. Soc. Lond. 155: 64, 1943.
= Isariopsispenicillata Ellis & Everh., Bull. Torrey bot. Club 22: 438, 1895.
≡ Phaeoisariopsispenicillata (Ellis & Everh.) S.C. Jong & E.F. Morris, Mycopath. Mycol. appl. 34: 271, 1968.
= Arthrobotryumtecomae Henn., Hedwigia 43: 397, 1904.
= Arthrobotryumcaudatum Syd. & P. Sydow, Etudes sur la Flore du Bas et Moyen Congo 3(1): 22, 1909.
= Arthrobotryumdieffenbachiae F. Stevens, Bot. Gaz. 65: 237, 1918.
= Atractilinacallicarpae Dearn. & Barthol., Mycologia 16: 175, 1924.
= Podosporiumpallidum Pat., Scient. Surv. P. Rico 8(1) Bot.: 103, 1926.
= Eriomycopsisbosquieae Hansf., Bothalia 4(2): 466, 1942.
= Arthrobotryumdeightonii Hansf., Mycol. Pap. 15: 218, 1946.
= Malacariameliolicola Syd., Annls. Mycol. 28(1/2): 69, 1930. New synonym proposed in this study.
= Paranectriaflagellata Hansf., Proc. Linn. Soc. London 153(1): 28, 1941. New synonym proposed in this study.
≡ Malacariaflagellata (Hansf.) Hansf., Mycol. Pap. 15: 128, 1946. New synonym proposed in this study.

###### Description.

***Colonies*** effuse, rust brown or pale brown, with hyphae that form large, erect, dark synnemata clearly visible under the stereomicroscope, but sometimes only loose unstalked tufts around the tips of the setae of the meliolalean host. ***Hyphae*** superficial, branched, septate, thin-walled, 1–2.5 µm wide, smooth. ***Conidiophores*** may form straw-coloured or pale olivaceous synnemata up to 1.5 mm long, 40 µm wide at the basal stalk-like part. Sometimes the synnemata grow around and up the setae of the meliolalean host. Individual conidiophores straight or sometimes flexuous, cylindrical, 2.5–5 µm thick towards the apex, pale olivaceous brown, with denticles. ***Conidia*** solitary, straight or slightly curved, fusiform, truncate at the base, tapering towards the apex and often terminating in a little bulbous swelling, 1 to mostly 3 septate, thin-walled, variable in size, (17–)30–37(–80) × (3.5–)7–8.5 µm, at first more or less colourless, at maturity becoming pale straw coloured, minutely rough-walled. As seen by SEM, the ornamentation of the surface of the conidia is distinctly reticulated, with thin networks and no ridges.

**Figure 1.**
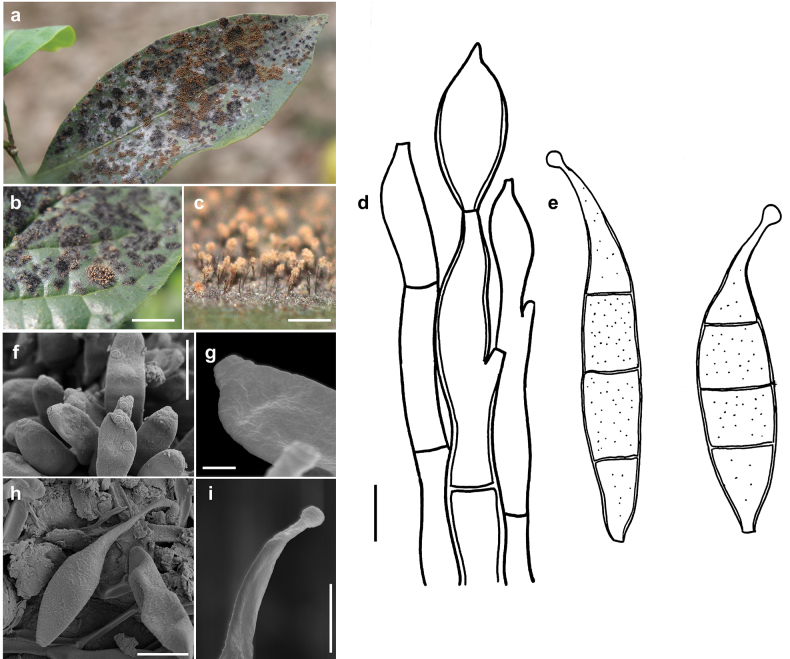
*Atractilinaparasitica* (MB127, MB136) **a** synnemata (gold spots) on colonies of *Meliola* sp. (black spots) on a leaf of *Opiliaceltidifolia***b** synnemata of (gold spots) on colonies of *Meliolaclerodendricola* (black spots) on a leaf of *Clerodendrumcapitatum***c** synnemata **d** conidiophores drawn in optical section. The thickness of the wall is indicated only in the drawing in the middle **e** conidia shown in optical section **f–i** as seen by SEM**f** conidiophores with denticles **g** a denticle at the tip of a conidiophore **h** conidium **i** bulbous swelling at the tip of a conidium. Scale bars: 1.5 mm (**b**); 1 mm (**c**); 5 μm (**d,e,i**); 8 μm (**f**); 1 μm (**g**); 6 μm (**h**).

###### Specimens examined.

On *Meliola* sp. on living leaves of *Opiliaceltidifolia* (Opiliaceae), Benin, Campus University of Abomey-Calavi, botanical garden, 6°25'7"N, 2°20'34"E, 24 m a.s.l., 9 February 2022, M. A. Bermúdez-Cova, A. Tabé, D. Dongnima, O.P. Agbani, M. Piepenbring, N.S. Yorou, MB127 (UNIPAR, M); on *Meliolaclerodendricola* on living leaves of *Clerodendrumcapitatum* (Lamiaceae), Benin, Abomey-Calavi, Zopah, 6°30'8"N, 2°20'24"E, 37 m a.s.l., 12 February 2022, M. A. Bermúdez-Cova, A. Tabé, D. Dongnima, O.P. Agbani, M. Piepenbring, N.S. Yorou, MB133; on *Meliolaclerodendricola* on living leaves of *Clerodendrumcapitatum*, Benin, Allada, Sékou, 6°38'56"N, 2°11'38"E, 48 m a.s.l., 12 February 2022, M. A. Bermúdez-Cova, A. Tabé, D. Dongnima, O.P. Agbani, M. Piepenbring, N.S. Yorou, MB136 (UNIPAR, M, GenBank accession number: OR804686); on *Meliola* sp. on living leaves of *Pterocarpussantalinoides* (Fabaceae), Benin, Lokoli, border of forest, 7°3'41"N, 2°15'26"E, 22 m a.s.l., 20 February 2022, M. A. Bermúdez-Cova, A. Tabé, D. Dongnima, L. Konetche, M. Piepenbring, R. Hounkarin, MB160 (M); on *Meliola* sp. on living leaves of *Coffeaarabica* (Rubiaceae), Benin, Attogon, Niaouli, CRA-Sud center, 6°44'24"N, 2°8'25"E, 122 m a.s.l., 28 February 2022, M. A. Bermúdez-Cova, A. Tabé, I. Agonglo, M. Piepenbring, N.S. Yorou, O.P. Agbani, MB178 (UNIPAR, M, GenBank accession numbers: OR804685 and OR804687); on *Meliola* sp. on living leaves of *Coffeaarabica*, Benin, Atlantique, Attogon, Niaouli Forest, 6°44'23"N, 2°8'26"E, 119 m a.s.l., 19 September 2022, A. Krauß, A. Tabé, O. Koukol, N.S. Yorou, AK06H (UNIPAR, M, GenBank accession number: OR804684); on *Meliola* sp. on living leaves of *Clerodendrumcapitatum*, Benin, Atlantique, Attogon, Pahou Forest, 6°22'56"N, 2°9'35"E, 13 m a.s.l., 6 October 2022, A. Krauß, A. Tabé, O. Koukol, N.S. Yorou, AK61.

###### Additional specimens examined.

On *Meliolalasiotricha* on leaves of unknown plant host, Puerto Rico, 1926, M.B. Ellis (IMI 130722, type specimen of *Podosporiumpallidum*); On *Meliolaclerodendri* on leaves of *Clerodendrumcyrtophyllum*, Taiwan, 1938, W. Yamamoto (IMI 31921b, type specimen of *Atractilinaparasitica*).

###### Illustrations.

This species was illustrated by Deighton and Pirozynski (1972).

###### Known hosts and distribution.

On colonies of *Amazonia* spp., *Asteridiella* spp., *Irenopsis* spp. and *Meliola* spp. on living leaves of various plants in Congo, Ghana, Guinea, India, Mauritius, Nigeria, Perú, Philippines, Puerto Rico, Sierra Leone, St. Thomé, Taiwan, Tanzania, Uganda, U.S.A. and Venezuela. Only one single collection on *Balladyna* sp. (Balladynaceae, Dothideomycetes) as a fungal host (Deighton and Pirozynski 1972). *Atractilinaparasitica* is reported here for the first time for Benin.

###### Notes.

Only two species of the genus *Atractilina* with hyperparasitic lifestyle are known, namely *A.asterinae* and *A.parasitica* (Deighton and Pirozynski 1972). *Atractilinaasterinae* differs from *A.parasitica* by the presence of 3–10 septate, thick-walled conidia.

The specimens of *A.parasitica* collected on leaves of *Coffeaarabica* (MB 178, AK4H, AK06H) were found growing together with pseudothecia of *Malacariameliolicola* Syd. (Tubeufiales, Dothideomycetes). According to Hansford (1941, as *Paranectriaflagellata*; 1946), *M.flagellata* is most probably the perfect state of *A.parasitica*. The specimens collected by Hansford were also growing on coffee leaves. The latter and the fact that the DNA sequences we obtained from *A.parasitica* (GenBank accession numbers: OR804684, OR804686, OR804685 and OR804687) and *M.meliolicola* (GenBank accession numbers: OR805247 and OR805248) clustered together in one single strongly-supported clade (Fig. 22), confirm the anamorph-teleomorph connection between both species. For an updated species description of *M.meliolicola*, see Bermúdez-Cova et al. (2023b).

#### ﻿*Spiropes* Cif., Sydowia 9(1–6): 302, 1955

##### ﻿ Spiropes angylocalycis

Taxon classificationFungiMeliolalesAscomycota

Berm.-Cova & M. Piepenbr. sp. nov.

C3D553E4-6AB9-5649-8BC7-F994A00E7FDB

MycoBank No: 850990

Fig. 2

###### Holotype.

On *Meliola* sp. on living leaves of *Angylocalyxoligophyllus* (Fabaceae), Benin, Atlantique, Attogon, Niaouli Forest, 6°44'42"N, 2°7'50"E, 69 m a.s.l., 28 February 2022, M.A. Bermúdez, A. Tabé, D. Dongnima, I. Agonglo, O.P. Agbani, M. Piepenbring, N.S. Yorou, MB167 (M).

**Figure 2.**
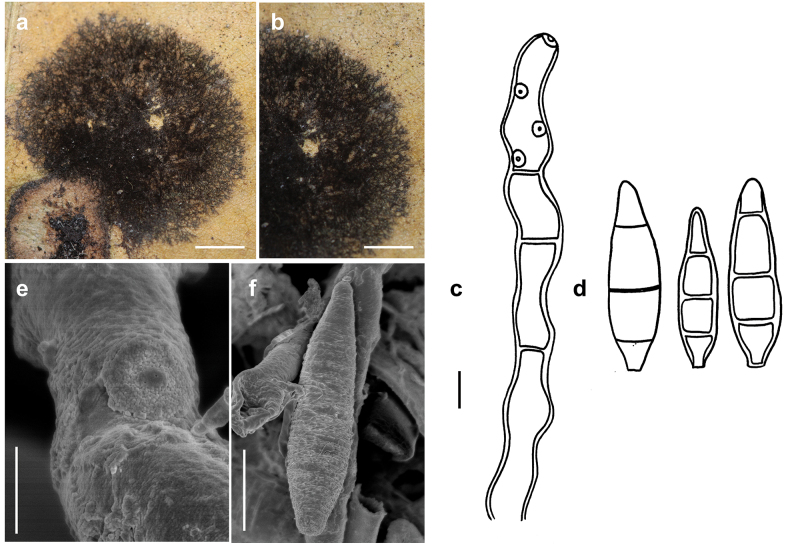
*Spiropesarmatellae* (MB 167) **a, b** conidiophores growing intermingled with hyphae of *Meliola* sp. on leaves of *Angylocalyxoligophyllus***c** conidiophore with scars **d** conidia shown in optical section. The thickness of the wall is shown in the two drawings on the right-hand side **e, f** as seen by SEM**e** part of a conidiophore with scar **f** conidium. Scale bars: 0.3 mm (**a**); 0.2 mm (**b**); 5 μm (**c, d**); 2 μm (**e**); 7 μm (**f**).

###### Etymology.

Named after the genus of the host plant.

###### Description.

***Colonies*** effuse, dark brown to black, velvety to hairy. ***Hyphae*** superficial, branched, anastomosing, septate, 0.5–2 µm wide, straw-coloured, smooth. ***Conidiophores*** arising singly, erect or ascending, straight to flexuous, mostly flexuous at the tips, septate, up to 350 µm long, 4–6 µm thick, pale olivaceous-brown to brown, with rough surface, with scattered scars mostly in upper parts of the conidiophores. ***Conidia*** solitary, straight or slightly curved, fusiform to obclavate, 3–septate, (15–)17–25(–30) × 5–6.5 µm, 2–3 µm wide at the base, brown, the cells at each end pale brown, septa darker in colour, verrucose. As seen by SEM, the ornamentation of the spores is distinctly reticulated, with thin to thick networks and no ridges.

###### Known distribution.

On colonies of *Meliola* sp. on living leaves of *Angylocalyxoligophyllus* in Benin.

###### Notes.

*Spiropesangylocalycis* is similar to *S.clavatus* by the presence of 3–septate mostly fusiform conidia, with a similar size range (Ellis 1968). However, the conidiophores of *S.clavatus* are synnematous, while they are mononematous in *S.angylocalycis*.

##### ﻿ Spiropes armatellae

Taxon classificationFungiMeliolalesAscomycota

M.B. Ellis, Mycol. Pap. 125: 15, 1971

20569C14-36AD-5541-9681-61B2AC859D69

Fig. 3

###### Type.

On *Armatellacinnamomicola* on leaves of *Cinnamomum* sp. (Lauraceae), Sri Lanka, Ceylon, 1971, M.B. Ellis (IMI134405b. The type specimen was not available for loan).

**Figure 3.**
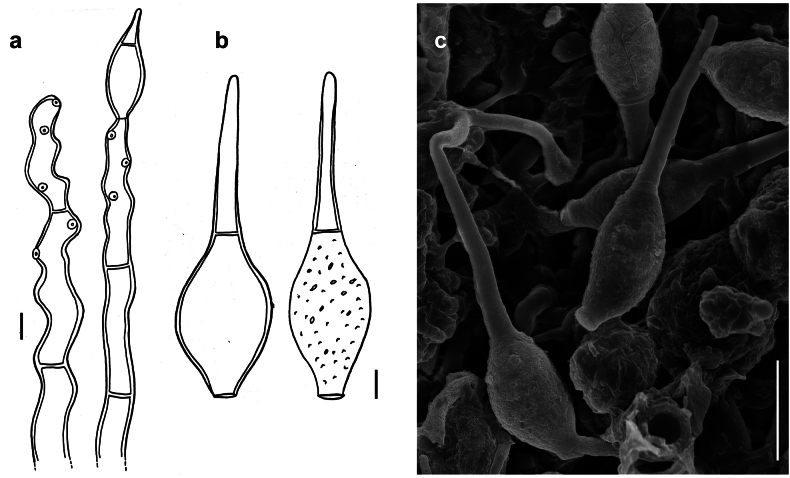
*Spiropesarmatellae* (IMI 161265) **a** conidiophores with young conidium **b, c** conidia **b** shown in optical section. The thickness of the wall is indicated only in the drawing on the left-hand side **c** as seen by SEM. Scale bars: 5 μm (**a**); 2.5 μm (**b**); 10 μm (**c**).

###### Description.

***Colonies*** effuse, dark brown to black, hairy. ***Hyphae*** superficial, branched, septate, 1–3 µm wide, straw-coloured, smooth. ***Conidiophores*** arising singly, erect or ascending, straight to flexuous, mostly flexuous at their tips, septate, up to 300 µm long, 5–8 µm thick, brown to dark brown, paler towards the apex, with rough surface, with scattered scars in upper parts of the conidiophores. ***Conidia*** solitary, straight or slightly curved, obclavate to obpyriform, mostly 1–septate, (20–)30–42(–50) × (6–)7–8(–10) µm, 2–3.5 µm wide at the base, brown, paler towards the ends, verrucose when seen by LM and SEM.

###### Specimen examined.

On *Armatellalitseae* on leaves of *Daphnidiumpulcherrimum* (Lauraceae), India, west Bengal, 1967, M.K. Maity (IMI 136371); on *Armatellacinnamomicola* on leaves of *Cinnamomum* sp., Myanmar, Thaton, 1971, M.M. Thaung, (IMI 161265).

###### Known hosts and distribution.

On colonies of *Armatella* spp. on various plants in India, Myanmar and Sri Lanka (Ellis 1971).

###### Illustrations.

This species was illustrated by Ellis (1971).

###### Notes.

Two known species of *Spiropes* are hyperparasitic on species of the genus *Armatella* (Meliolales, Armatellaceae), namely *S.armatellae* and *S.armatellicola* (Ellis 1971, Hosagoudar et al. 2002). According to Hosagoudar et al. (2002), both species are similar, but differ by the ornamentation of the conidia. The conidia of *S.armatellicola* are smooth, while those of *S.armatellae* are distinctly reticulated. However, it is sometimes difficult to observe the surface of the conidia by LM. Therefore, we recommend to analyse the ornamentation of the spores of *S.armatellicola* by SEM. The scars of *S.armatellae* could not be observed by SEM and it is necessary to collect fresh specimens of this fungus for further morphological analysis.

##### ﻿ Spiropes armatellicola

Taxon classificationFungiMeliolalesAscomycota

M.B. Ellis, Mycol. Pap. 125: 15, 1971

A72945E1-11E7-5AE7-BC81-35E336BF2117

###### Type.

On *Armatella* sp. on leaves of *Actinodaphne* sp. (Lauraceae), Banasuran Hills, Wyanad, Kerala, India, 16 April 1999, C.K. Biju (HCIO 43621. The type specimen was not available for loan by HCIO).

###### Species description.

This species was described by Hosagoudar et al. (2002).

###### Known hosts and distribution.

On colonies of *Armatella* sp. on living leaves of *Actinodaphne* sp. in India (Hosagoudar et al. 2002).

###### Illustrations.

This species was illustrated by Hosagoudar et al. (2002).

###### Notes.

This species is only known from the type specimen.

##### ﻿ Spiropes capensis

Taxon classificationFungiMeliolalesAscomycota

(Thüm.) M.B. Ellis, Mycol. Pap. 114: 5, 1968

7348AD6E-3596-57D8-8EBB-46436A16460C

Fig. 4

≡ Cercosporacapensis (Thüm.) Sacc., Syll. fung. 4: 469, 1886.
≡ Helminthosporiumcapense (Thüm.) [as ‘Helmisporium’], Flora, Regensburg 59: 570, 1876.
≡ Pleurophragmiumcapense (Thüm.) S. Hughes, Can. J. Bot. 36: 796, 1958.
= Helminthosporiumcarpocrinum Cif. [as ‘Helmisporium’], Annls. Mycol. 36(2/3): 236, 1938.
= Helminthosporiumcoffeae Massee [as ‘Helmisporium’], Bull. Misc. Inf., Kew: 167, 1901.
≡ Sporhelminthiumcoffeae (Massee) Speg., Physis, Rev. Soc. Arg. Cienc. Nat. 4(no. 17): 292, 1918.
= Helminthosporiumfici H.S. Yates [as ‘*ficuum*’], Philipp. J. Sci. (Bot.) 13: 382, 1918.
= Helminthosporiumficinum Sacc. [as ‘Helmisporium’], Atti Accad. Sci. Ven.-Trent.-Istr., Sér. 3, 10: 90, 1919.
= Helminthosporiumfumagineum Sacc. [as ‘Helmisporium’], Atti Accad. Sci. Ven.-Trent.-Istr., Sér. 3, 10: 90, 1919.
= Helminthosporiumfilicicola Henn., Hedwigia 44: 71, 1905.
= Helminthosporiumglabroides F. Stevens [as ‘Helmisporium’], Bot. Gaz. 65(3): 240, 1918.
= Helminthosporiummelioloides Sacc. [as ‘Helmisporium’], Atti Accad. Sci. Ven.-Trent.-Istr., Sér. 3, 10: 89, 1919.
= Helminthosporiumorbiculare Lév., Annls. Sci. Nat., Bot., Sér. 3, 5: 299, 1846.
= Helminthosporiumphilippinum Sacc. [as ‘Helmisporium’], Atti Accad. Sci. Ven.-Trent.-Istr., Sér. 3, 10: 89, 1919.
= Helminthosporiumsubsimile Sacc., Boll. Orto bot., Napoli 6: 23, 1921.
= Helminthosporiumtapurae Allesch., Hedwigia 36(4): 245, 1897.
= Napicladiumportoricense Speg., Boln Acad. nac. Cienc. Córdoba 26(2–4): 363, 1921.
≡ Helminthosporiumportoricense (Speg.) Cif., Sydowia 9(1–6): 298, 1955.
= Nascimentoapseudoendogena Cif. & Bat., Publicações Inst. Micol. Recife 44:4, 1956.

###### Description.

***Colonies*** effuse, dark brown to black, hairy (Ellis 1968). ***Hyphae*** superficial, branched, septate, 2–4 µm wide, pale olive to olivaceous-brown, smooth. ***Conidiophores*** arising singly or in groups, sometimes in large groups of 50–100 conidiophores, terminally or laterally from the hyphae, erect or ascending, straight or flexuous, septate, up to 600 µm long, 5–9 µm thick along most of their length, brown to dark brown, paler closer to the apex, with terminal and lateral scars. ***Conidia*** solitary, straight or curved, fusiform to obclavate, truncate at the base, 3–6 (usually 4 or 5) pseudosepta, (33–)50–60(–78) × (5.5–)6–11(–16) µm, 1–4 µm wide at the base, light brown to brown, smooth.

**Figure 4.**
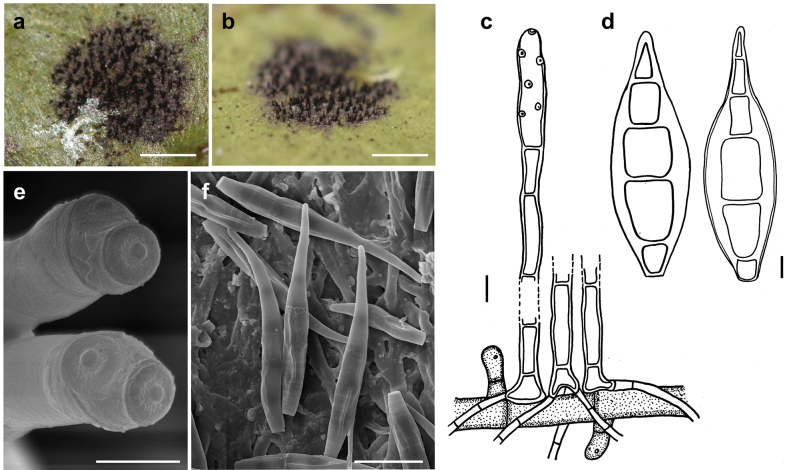
*Spiropescapensis* (AK06H) **a, b** groups of conidiophores growing on hyphae of *Meliola* sp **c** conidiophores growing on hyphae of *Meliola* sp. shown in optical section **d** conidia shown in optical section. The thickness of the outer wall layer is indicated only in the drawing on the right-hand side **e, f** as seen by SEM**e** conidiophores with scars **f** conidia. Scale bars: 1 mm (**a, b**); 8.5 μm (**c**); 5 μm (**d**); 5 μm (**e**); 20 μm (**f**).

###### Specimen examined.

On *Meliola* sp. on living leaves of *Coffeaarabica*, Benin, Atlantique, Attogon, Niaouli Forest, 6°44'23"N, 2°8'26"E, 119 m a.s.l., 19 September 2022, A. Krauß, A. Tabé, O. Koukol, N.S. Yorou, AK06H.

###### Additional specimens examined.

– On leaves of *Ficusulmifolia* (Moraceae), Philippines, Los Baños, 1915, C.F. Baker, 451 (IMI 130940, type of *Helminthosporiumfumagineum*); on *Meliolacompositarum* on leaves of *Eupatoriumportoricense* (Asteraceae), Puerto Rico, Bega Vaja, 1921, no. 1753 (IMI 100331a, type of *Napicladiumportoricense*).

###### Known hosts and distribution.

On colonies of *Appendiculella* spp., *Asteridiella* spp., *Irenopsis* spp. and *Meliola* spp. on living leaves of various plants in Amboina, Bolivia, Brazil, Cameroon, Congo, Dominican Republic, Ghana, India, Jamaica, Malaya, Peru, Philippines, Puerto Rico, Sabah, Sierra Leone, South Africa, Tanzania, Trinidad, Uganda and Venezuela (Ellis 1968); on *Meliola* sp. on living leaves of *Coffeaarabica* in Benin (this study). *Spiropescapensis* is reported here for the first time for Benin.

###### Illustrations.

This species was illustrated by Ellis (1968).

###### Notes.

According to the nomenclatural and taxonomic database Index Fungorum (http://www.IndexFungorum.org), the current name of the *Spiropescapensis* is *Pleurophragmiumcapense* (Thüm.) S. Hughes. The genus *Pleurophragmium* (*incertae sedis*, Ascomycota) was established by Costantin (1888) and it comprises species with brown to dark brown conidiophores and sympodially proliferating, denticulate conidiogenous cells producing holoblastic, simple, mostly 3–septate, brown to dark brown conidia (Abarca et al. 2007). According to Ellis (1968), the flat double scar is a good taxonomic character to distinguish species of *Spiropes* from *Pleurophragmium*, since, in the latter, the conidia are borne at the tips of tapered denticles. The morphological analysis of our samples and the type specimens (AK06H, IMI 100331a and IMI 130940) revealed the presence of flat double scars (Fig. 4e) and no denticles. We think that the examined species differs morphologically from species in the genus *Pleurophragmium* and, therefore, it should be retained in the genus *Spiropes*.

##### ﻿ Spiropes caribensis

Taxon classificationFungiMeliolalesAscomycota

Hol.-Jech., Česká Mykol. 38(2): 113, 1984

7A205FAD-CAD6-5D4A-AF87-01F216FDC3EC

Fig. 5

###### Description.

***Colonies*** effuse, dark brown to black, velvety to hairy. ***Hyphae*** superficial, branched, septate, 1.5–3.5 µm wide, pale olivaceous-brown, smooth. ***Conidiophores*** arising singly, erect or ascending, straight or flexuous, septate, up to 240 µm long, 4–8 µm thick, pale brown to brown, smooth, with few scars. ***Conidia*** solitary, straight or slightly curved, obclavate, central cells barrel-shaped, 3-septate, (30–)36–48(–41.5) × (7.5–)9.5–11.5 µm, 4.5–6 µm wide at the truncate base, the central cells pale brown, the cells at the ends paler and almost hyaline, smooth.

###### Specimen examined.

On *Meliola* sp. on leaves of an unknown palm-tree, Cuba, Isla de La Juventud (= Isla de Pinos), Los Indios, south-west of La Cañada, 1981, V. Holubová-Jechová (PRM 831531, holotype).

###### Known hosts and distribution.

On *Meliola* sp. on living leaves of an unidentified palm tree in Cuba (Holubová-Jechová and Sierra 1984).

###### Illustrations.

This species was illustrated by Holubová-Jechová and Sierra (1984).

###### Notes.

**Figure 5.**
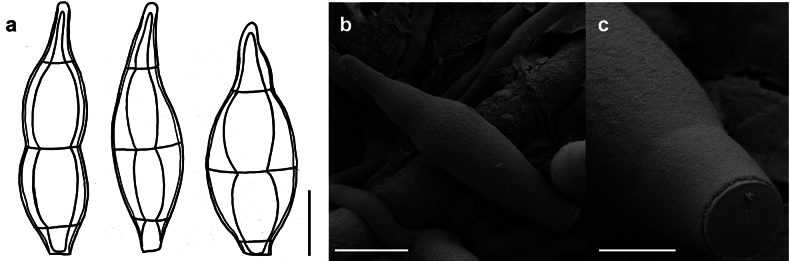
*Spiropescaribensis* (PRM 8311531) **a** conidia shown in optical section **b, c** as seen by SEM**b** conidium **c** basis of a conidium with a flat scar. Scale bars: 10 μm (**a**); 9 μm (**b**); 4 μm (**c**).

*Spiropescaribensis* is similar to *S.helleri*, but differs from the latter by paler conidia, with wider truncate base (*S.helleri* has conidia with a truncate base 3–4 µm wide) and shorter conidiophores (up to 600 µm long in *S.helleri*; Holubová-Jechová and Sierra (1984)). As seen by SEM, conidia of *S.caribensis* are smooth (Fig. 5b), while conidia of *S.helleri* are distinctly reticulated (Fig. 13e). The scars could not be observed by SEM and it is, therefore, necessary to collect fresh specimens of this fungus for further morphological analyses. *S.caribensis* is only known from the type specimen.

##### ﻿ Spiropes carpolobiae

Taxon classificationFungiMeliolalesAscomycota

Berm.-Cova & M. Piepenbr. sp. nov.

DA2405BF-7586-5D68-A5BC-E2F40DB7DE78

MycoBank No: 850987

Fig. 6

###### Holotype.

On Meliolacf.carpolobiae on living leaves of *Carpolobialutea* (Polygalaceae), Benin, Atlantique, Attogon, Niaouli Forest, 6°44'41"N, 2°7'52"E, 68 m a.s.l., 28 February 2022, M.A. Bermúdez, A. Tabé, D. Dongnima, I. Agonglo, O.P. Agbani, M. Piepenbring, N.S. Yorou, MB166 (M).

**Figure 6.**
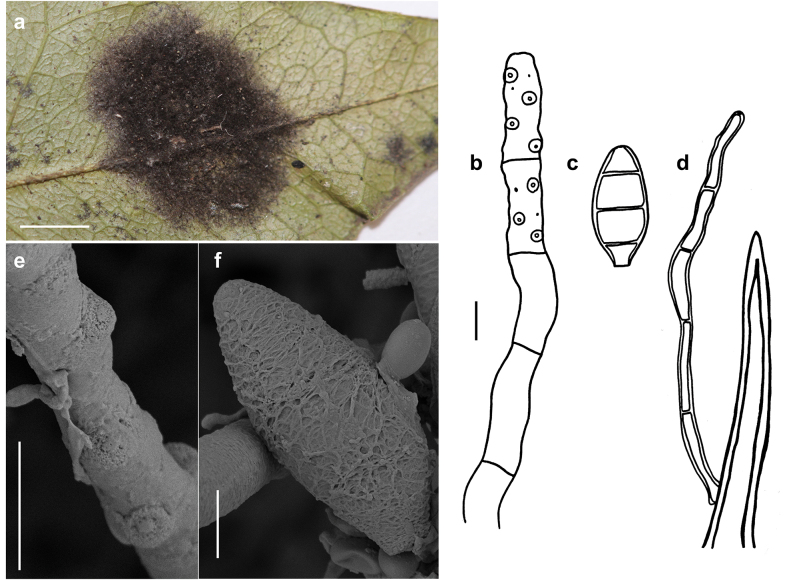
*Spiropescarpolobiae* (MB 166) **a** conidiophores growing intermingled with hyphae of *Meliola* sp. on a leaf of *Carpolobialutea***b** conidiophore with scars **c** Conidia shown in optical section. The thickness of the wall is shown in the left-hand drawing **d, e** as seen by SEM**d** conidiophore with scar **e** conidium. Scale bars: 0.3 mm (**a**); 5 μm (**b, c**); 5 μm (**d**); 3 μm (**e**).

###### Etymology.

Named after the genus of the host plant.

###### Description.

***Colonies*** effuse, dark brown to black, velvety to hairy. ***Hyphae*** superficial, branched, anastomosing, septate, 1–2 µm wide, straw-coloured, smooth. ***Conidiophores*** arising singly, erect or ascending, straight to flexuous, septate, up to 250 µm long, 2–5 µm thick, sometimes thicker at the apex, brown, not smooth, with scattered scars mostly in the upper parts of the conidiophores. ***Conidia*** solitary, straight or slightly curved, ovate to slightly fusiform, 3–septate, (12.5–)13–16(–19) × 5–7 µm, 2–2.5 µm wide at the base, brown, the cells at each end pale brown, septa darker, surface verrucose. As seen by SEM, the ornamentation of the conidia is distinctly reticulated, with thin to thick networks that can form ridges.

###### Known distribution.

On colonies of Meliolacf.carpolobiae on living leaves of *Carpolobialutea* in Benin.

###### Notes.

*S.carpolobiae* is the only known species of *Spiropes* with ovate to slightly fusiform conidia.

##### ﻿ Spiropes clavatus

Taxon classificationFungiMeliolalesAscomycota

(Ellis & Martin) M.B. Ellis, Mycol. Pap. 114: 25, 1968

D3F3C808-60A7-54D8-BD5E-1A5CCADD282D

Fig. 7

≡ Isariopsisclavata Ellis & Martin, Am. Nat. 18: 188, 1884.
≡ Arthrobotryumclavatum (Ellis & Martin) Höhn, Sber. Akad. Wiss. Wien, Math.-naturw. Kl., Abt. 1, 125: 120, 1916.
≡ Bitunicostilbeclavata (Ellis & Martin) M. Morelet, Bull. Soc. Sci. nat. Arch. Toulon et du Var 7: 195, 1971.
= Podosporiumchlorophaeum Speg., An. Mus. nac. Hist. nat. B. Aires 20: 450, 1910.
= Arthrobotryumnoz-moscatae Bat. & J. Silva, Anais IV Congr. Soc. bot. Brasil: 144, 1953.

###### Description.

***Colonies*** effuse, brown to dark brown or black. ***Hyphae*** superficial, branched, anastomosing, septate, 1–3 µm wide, pale olivaceous-brown. ***Conidiophores*** tightly packed to form dark brown to blackish synnemata up to 700 µm long, 20–40 µm thick, often splaying out to a width of up to 110 µm at the apex. Individual hyphae straight or flexous, cylindrical, 1–3 µm thick near the base, 4–7 µm thick near the apex, dark brown, paler towards the apex, verrucose, with numerous conidial scars. ***Conidia*** solitary, fusiform to obclavate, mostly 3–, rarely 1–, 2– or 4–septate, (13–)18–25(–33) × (4–)5–7(–8) µm, tapering to about 1–1.5 µm at the apex and at the base, pale brown to brown, the cells at each end paler, wrinkled. As seen by SEM, the ornamentation of the spores is distinctly reticulated, with thin to thick networks and no ridges.

###### Specimens examined.

On *Meliolapanici* on leaves of *Panicumglutinosa*, Puerto Rico, El Alto de la Bandera, 1913, F.L. Stevens & W.E. Hess, n°4368 (IMI 130764); on *Meliola* sp. on leaves of *Raphiamonbuttorum*, Uganda, 1915, R. Dümmer, (IMI 102772); on *Meliolathouiniae* on leaves of an unknown plant, Brasil, São Paulo, 1940, A.R. Campos (IMI 130975, type of *Arthrobotryumnoz-moscatae*).

###### Illustrations.

This species was illustrated by Ellis (1968).

###### Known hosts and distribution.

On colonies of Meliolales on living leaves of various plants in Argentina, Brazil, Ghana, Malaysia, Puerto Rico, Sierra Leone, Trinidad and Uganda (Ellis 1968).

###### Notes.

**Figure 7.**
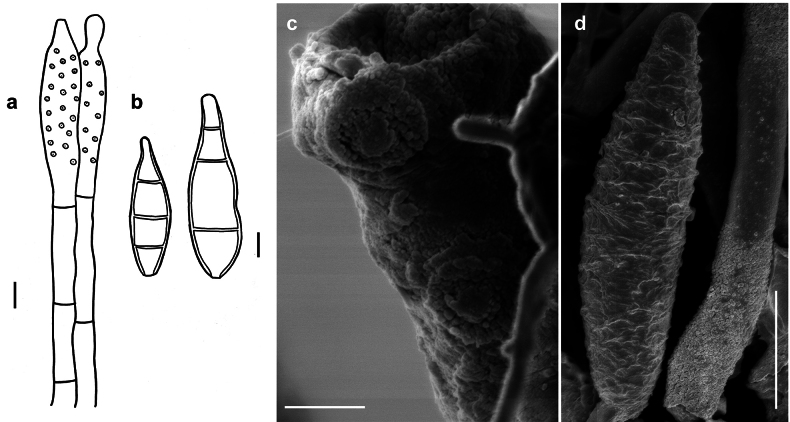
*Spiropesclavatus* (IMI 102772) **a** conidiophores with scars **b** conidia shown in optical section **c, d** as seen by SEM**c** conidiophore with scars **d** conidium. Scale bars: 5 μm (**a**); 2.5 μm (**b**); 1 μm (**c**); 5 μm (**d**).

In the nomenclatural and taxonomic database Index Fungorum (http://www.IndexFungorum.org), the current name of the *Spiropesclavatus* is *Bitunicostilbeclavata* (Ellis & Martin) M. Morelet. The genus *Bitunicostilbe* (*incertae sedis*, Ascomycota) was proposed by Morelet (1971) to accommodate two species, namely *B.clavata* and *B.linderae*, that were previously cited in other genera. Although the publication by Morelet was not available for this study, the morphological analysis of the herbarium specimens (IMI 130764, 130975) revealed that the features of these specimens are consistent with the description of *Spiropesclavatus* by Ellis (1968). The species has typical characteristics of the genus *Spiropes*, such as flat double scars (Fig. 7c) and, therefore, it should be classified in this genus. De Beer et al. (2013) analysed the type and additional specimens of *B.linderae* (as *Graphiumlinderae*) and concluded that this species should be also classified in the genus *Spiropes*.

##### ﻿ Spiropes croissantiformis

Taxon classificationFungiMeliolalesAscomycota

Berm.-Cova & M. Piepenbr. sp. nov.

1E3BA541-260E-528F-A781-DD1D154ACCBD

MycoBank No: 850984

Fig. 8

###### Holotype.

On Meliolacf.xylopiae on living leaves of *Xylopiafrutescens*, Panama, Chiriquí Province, Cochea, Cochea River Trail, 8°32'37"N, 82°23'03"W, 181 m a.s.l., 26 February 2020, M.A. Bermúdez, A. Sanjur, A. Villarreal, MB110 (UCH).

###### Etymology.

Named after the shape of the conidia.

###### Description.

***Colonies*** effuse, dark brown to black, with tightly packed hyphae that form erect, dark synnemata clearly visible under the stereomicroscope. ***Hyphae*** superficial, branched, septate, 1–2 µm wide, straw-coloured, smooth. ***Conidiophores*** tightly packed to form dark brown to blackish synnemata up to 400 µm high, spreading out at the apex, up to 80 µm diam. Individual hyphae mostly straight, cylindrical, 3–5 µm thick, with numerous small scars, brown, paler towards the apex, rough. ***Conidia*** straight or curved, mostly crescent-shaped, sometimes fusiform, mostly 3(–5)–septate, (14–)20–24(–33) × (3.5–)5–6.5 µm, with two golden brown middle cells and paler cells at each. As seen by SEM, the ornamentation of the spores is distinctly reticulated, with thin to thick networks and no ridges.

###### Known distribution.

On colonies of Meliolacf.xylopiae on living leaves of *Xylopiafrutescens* (Annonaceae) in Panama.

###### Notes.

*Spiropesxylopiae* is a synnematous hyperparasitic species of *Spiropes* with the shortest synnemata (up to 400 µm), when compared to other synnematous species, such as *S.melanoplaca* with synnemata that can reach up to 1.5 mm and *S.penicillium* with synnemata up to 700 µm high. In addition to this, the new species has crescent-shaped conidia, a feature that is not present in any other known species of the genus.

**Figure 8.**
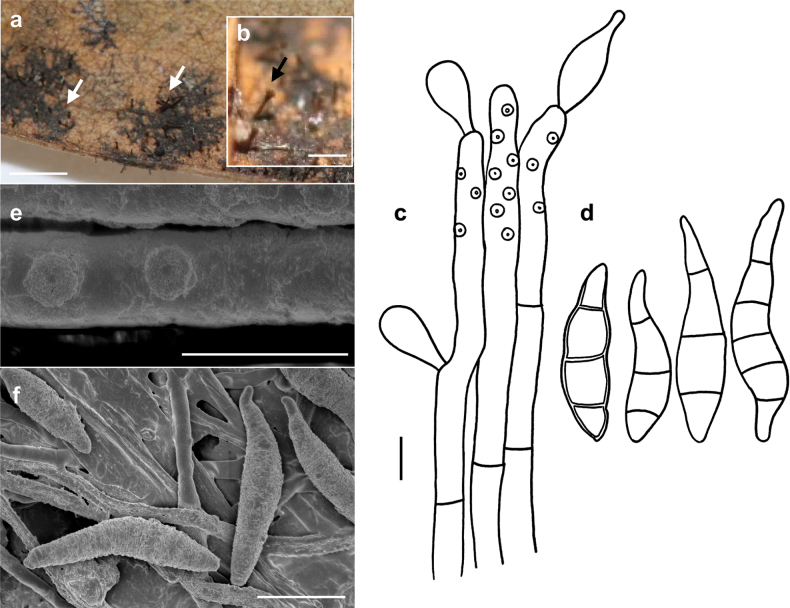
*Spiropescroissantiformis* (MB 110) **a** synnemata (indicated by white arrows) growing on colonies of Meliolacf.xylopiae**b** synnema (indicated by a black arrow) **c** conidiophores with scars and young conidia, shown in optical section **d** conidia shown in optical section. The thickness of the wall is only shown for the first spore from the left **e, f** as seen by SEM**e** part of a conidiophore with scars **f** conidia. Scale bars: 160 μm (**a**); 400 μm (**b**); 5 μm (**c, d**); 5 μm (**e**); 10 μm (**f**).

##### ﻿ Spiropes deightonii

Taxon classificationFungiMeliolalesAscomycota

M.B. Ellis, Mycol. Pap. 114: 18, 1968

A4B397F1-FA33-58E1-8DCE-25243F47CA16

Fig. 9

###### Description.

***Colonies*** effuse, olive to olivaceous-brown, velvety or hairy. ***Hyphae*** superficial, branched, septate, 0.5–2 µm wide, pale olive to olivaceous-brown, smooth. ***Conidiophores*** arising singly or in groups terminally or laterally from the hyphae, erect or ascending, straight or flexous, septate, up to 400 µm long, 2–4 µm thick along most of their length, swollen towards the apex, 5–8 µm thick, brown, reticulate as seen by SEM, with scattered cylindrical scars. ***Conidia*** solitary, straight or slightly curved, obovate to clavate, truncate at their base, 3–septate, (10–)12–14(–15) × (5–)6–8 µm, 1.5–2 µm wide at the base, the cells at each end of a conidium subhyaline or pale brown, intermediate cells brown, ornamented. As seen by SEM, the ornamentation of the spores is distinctly reticulated, with thin to thick networks that can form ridges.

###### Specimen examined.

On *Meliolaborneensis* on *Uvariachamae*, Sierra Leone, 1951, F.C. Deighton, (IMI 48956a, type of *S.deightonii*).

###### Illustrations.

This species was illustrated by Ellis (1968).

###### Known hosts and distribution.

On colonies of *Meliolaborneensis* on living leaves of *Uvariachamae* (Annonaceae) in Sierra Leone (Ellis 1968).

###### Notes.

**Figure 9.**
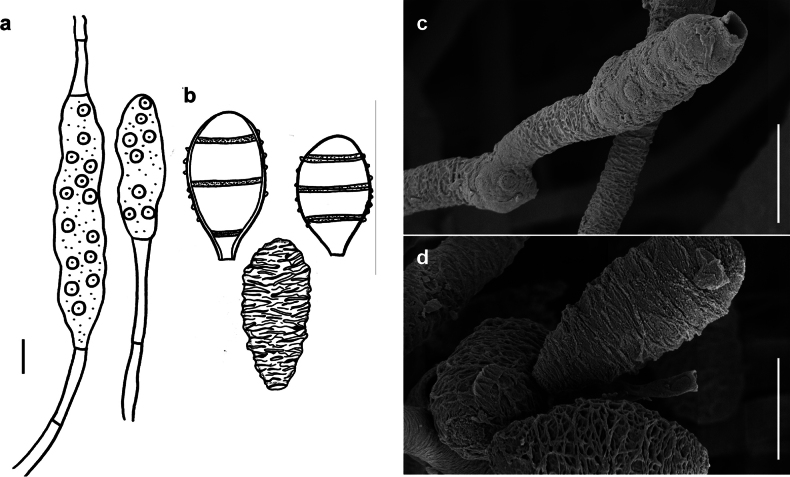
*Spiropesdeightonii* (IMI48956a) **a** conidiophores **b** conidia, as seen by LM (two upper spores; the thickness of the wall is indicated only in the drawing on the left-hand side) and by SEM (bottom spore) **c, d** as seen by SEM**c** conidiophore **d** conidia. Scale bars: 5 μm (**a, b**); 8 μm (**c**); 5 μm (**d**).

*Spiropesdeightonii* and *Spiropesintricatus* are the only known species of the genus that present conidiophores that swell in the areas where conidia are formed (Figs 9, 14; Ellis (1968)). *Spiropesintricatus* differs from *S.deightonii* by the presence of larger conidia (16–23 µm long) that are more oblong-ellipsoid (Ellis 1968), rather than obovate or clavate. *S.deightonii* is only known from the type specimen.

##### ﻿ Spiropes dorycarpus

Taxon classificationFungiMeliolalesAscomycota

(Mont.) M.B. Ellis, Mycol. Pap. 114: 27, 1968

E28AEF10-8BCD-5981-B9FB-0C4758E83E98

Fig. 10

≡ Helminthosporiumdorycarpum Mont., Annls Sci. nat., 2 Sér., 17: 120, 1842.
≡ Pleurophragmiumdorycarpum (Mont.) Hughes, Can. J. Bot. 36: 797, 1958.
= Helminthosporiumorbiculare Lév., Annls Sci. nat., 3 Sér., 5: 299, 1846.
= Napicladiummyrtacearum Speg., An. Soc. cient. Argent. 26: 71, 1888.
≡ Sporhelminthiummyrtacearum (Speg.) Speg., Physis 4(17): 292, 1918.
= Helminthosporiumconspicuum McAlpine, Proc. Linn. Soc. N.S.W. 22: 40, 1897.
= Podosporiumdensum Pat., J. Bot. Paris 11: 373, 1897.
= Helminthosporiumasterinoides Sacc. & P. Syd., apud Saccardo, Rc. Congr. Bot. Palermo, May 1902: 58, 1902.
≡ Sporhelminthiumasterinoides (Sacc. & Syd.) Speg., Physis 4(17): 292, 1918.
= Helminthosporiummelastomacearum F. Stevens, Bot. Gaz. 65: 242, 1918.
= Helminthosporiumpanici F. Stevens, Bot. Gaz. 65: 242, 1918.
= Helminthosporiumparathesicola [as ‘*parathesicolum*’] F. Stevens, Bot. Gaz. 65: 242, 1918.

###### Description.

***Colonies*** effuse, brown to dark brown, hairy. ***Hyphae*** superficial, branched, septate, 1–3 µm wide, straw-coloured, pale brown, smooth. ***Conidiophores*** arising singly or in groups, terminally or laterally from the hyphae, erect or ascending, straight or flexous, septate, up to 700 µm long, 3–7 µm thick, straw-coloured, pale brown to brown, with scattered cylindrical scars towards the apex. ***Conidia*** solitary, straight or slightly curved, variable in shape, but mostly obclavate to fusiform, truncate at the base, mostly 3–septate, but sometimes with 4 to 5 septa, (16–)20–35(–40) × (4.5–)5–7 µm, straw-coloured to pale brown, middle cells slightly darker, wrinkled or verrucose. As seen by SEM, the ornamentation of the spores is distinctly reticulated, with thin to thick networks and no ridges.

###### Specimen examined.

On *Meliola* sp. on living leaves of *Coffeaarabica*, Benin, Atlantique, Attogon, Niaouli Forest, 6°44'23"N, 2°8'26"E, 119 m a.s.l., 19 September 2022, A. Krauß, A. Tabé, O. Koukol, N.S. Yorou, AK06H.

###### Additional specimens examined.

On *Eugeniapungens*, Brasil, Guarapí, 1883, B. Balansa, 3939, (IMI 100322, type of *Napicladiummyrtacearum*); on *Meliola* sp. on leaves of an unknown plant, Cuba, R. de la Sagra (IMI 10002, type of *Helminthosporiumdorycarpum*).

###### Illustrations.

This species was illustrated by Ellis (1968).

###### Known hosts and distribution.

On colonies of *Appendiculella* spp., *Asteridiella* spp., *Clypeolella* spp., *Irenopsis* spp., *Meliola* spp. and *Schiffnerula* spp., on living leaves of various plants in Australia, Brazil, Chile, Congo, Cuba, Dominican Republic, Ghana, Guyana, India, Malaysia, Nigeria, Puerto Rico, Sierra Leone, South Africa, Taiwan, Tanzania and Uganda (Ellis 1968). *Spiropesdorycarpus* is reported here for the first time for Benin.

**Figure 10.**
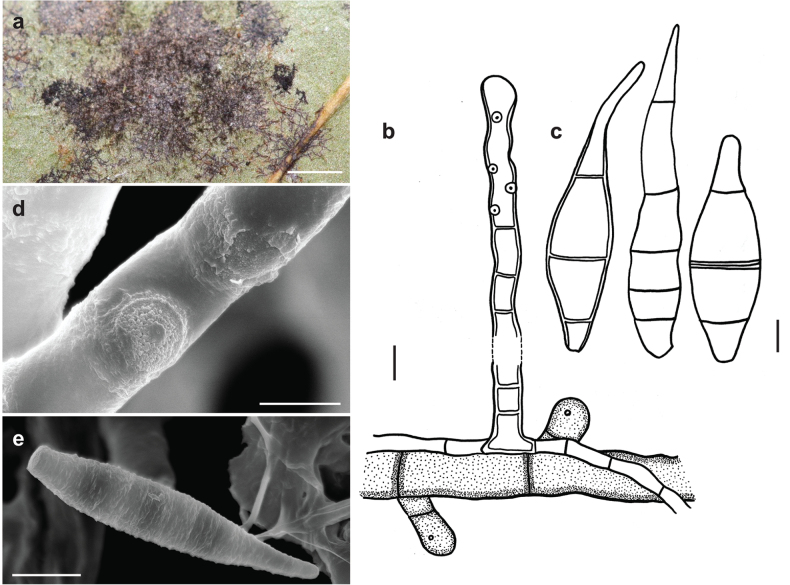
*Spiropesdorycarpus* (AK06H) **a** superficial hyphae growing on a colony of *Meliola* sp. on a leaf of *Coffeaarabica***b, c** in optical section **b** conidiophore growing on a hypha of *Meliola* sp. **c** conidia. The thickness of the wall is indicated only in the drawing on the left-hand side **d, e** As seen by SEM**d** conidiophore with a scar **e** conidium. Scale bars: 1 mm (**a**); 5 μm (**b**); 3.5 μm (**c**); 3 μm (**d**); 7 μm (**e**).

###### Notes.

*Spiropesdorycarpus* is similar to *S.effusus* and *S.helleri* by the presence of non-synnematous conidiophores and conidia mostly with three true septa. However, conidia of *S.effusus* are narrower (3–5 µm) than those of *S.helleri* (7–13 µm).

##### ﻿ Spiropes effusus

Taxon classificationFungiMeliolalesAscomycota

(Pat.) M.B. Ellis, Mycol. Pap. 114: 10, 1968

51BA9012-810A-5619-82CF-4B427971FC79

Fig. 11

≡ Podosporiumeffusum Pat., Scient. Surv. P. Rico 8(1): 103, 1926.
= Helminthosporiumdorycarpumvar.amazoniae Hughes [as ‘Helmisporium’], Mycol. Pap. 50: 24, 1953.
≡ Pleurophragmiumdorycarpumvar.amazoniae (S. Hughes) S. Hughes, Can. J. Bot. 36: 797, 1958.

###### Description.

***Colonies*** effuse, olive to brown, hairy. ***Hyphae*** superficial, branched, septate, 1–2 µm wide, yellowish, olive or pale brown, smooth. ***Conidiophores*** arising singly or in groups, as terminal and lateral branches on the hyphae, erect, straight or flexous, septate, up to 300 µm long, 3–4 µm thick, slightly reticulated when seen by SEM, with few or many small conidial scars towards the apex. ***Conidia*** solitary, narrowly obclavate to fusiform, truncate at the base, mostly 3(–5)–septate, (15–)20–36 × (3–)3.8–4.5(–5) µm, pale brown, the central cells slightly darker, verruculose. As seen by SEM, the ornamentation of the spores is distinctly reticulated, with thin networks and no ridges.

###### Specimen examined.

On meliolalean fungus on leaves of *Piper* sp., Puerto Rico, Río Piedras, 1926, Heller, 142 (IMI 130721, type of *Podosporiumeffusum*); on *Amazoniapsychotriae* on leaves of *Psychotriawarneckei*, Ghana, Togoland, 1938, F.C. Deighton M1617B (IMI 9996a).

###### Illustrations.

This species was illustrated by Ellis (1968).

###### Known hosts and distribution.

On colonies of Meliolales, especially *Amazonia* spp., on living leaves of various plants in Ghana, Puerto Rico, Sierra Leone and Venezuela. One record on *Asterina* sp. (Asterinales, Ascomycota) in Uganda (Ellis 1968).

**Figure 11.**
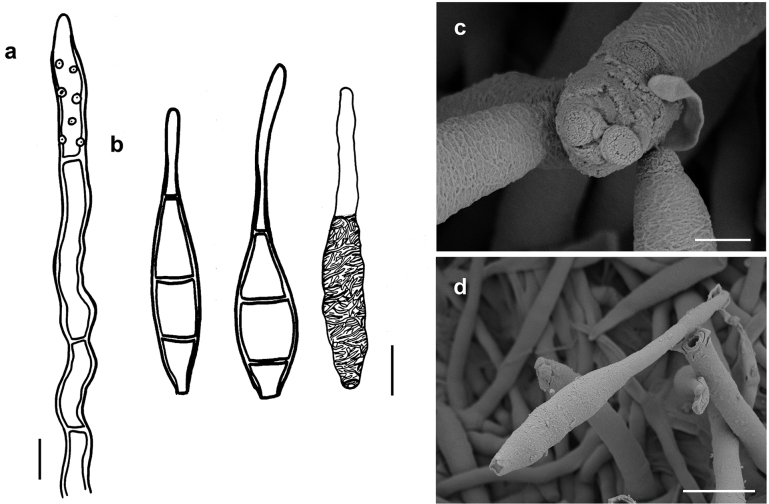
*Spiropeseffusus* (IMI 130721) **a** conidiophore shown in optical section **b** conidia. The first two drawings show spores in optical section. The right-hand drawing shows a conidium as seen by SEM**c, d** as seen by SEM**c** conidiophore with scars and conidia **d** conidium. Scale bars: 5 μm (**a**); 8 μm (**b**); 2 μm (**c**); 8 μm (**d**).

###### Notes.

*Spiropeseffusus* has conidia similar in size to those of *S.dorycarpus*. However, conidia of *S.dorycarpus* are wider (5–7 µm) than in *S.effusus*.

##### ﻿ Spiropes fumosus

Taxon classificationFungiMeliolalesAscomycota

(Ellis & Martin) M.B. Ellis, Mycol. Pap. 114: 20, 1968.

B7485883-C913-5AB8-8B5D-2B78CD468751

≡ Helminthosporiumfumosum Ellis & Martin, Am. Nat. 18: 70, 1884.
≡ Brachysporiumfumosum (Ellis & Martin) Sacc., Syll. Fung. 4: 428, 1886.

###### Type.

On *Meliola* sp. on leaves of *Perseapalustris* (Lauraceae), Florida, U.S.A, 1883, G. Martin (NY 830274. The type specimen was not available for loan by NY).

###### Species description.

This species was described by Ellis (1968).

###### Known hosts and distribution.

On colonies of *Meliola* sp. on living leaves of *Perseapalustris* in the U.S.A. (Ellis 1968).

###### Specimen examined.

On Meliolales on living leaves of *Perseapalustris*, U.S.A, Florida, Cove Springs, 1890, G. Martin, (IMI 16307).

###### Illustrations.

This species was illustrated by Ellis (1968).

###### Notes.

The specimen IMI 16307 was analysed, but no fungal cells were seen.

##### ﻿ Spiropes guareicola

Taxon classificationFungiMeliolalesAscomycota

(F. Stevens) Cif., Sydowia 9(1–6): 302, 1955

0B675A81-E0B4-557C-A908-0FF97F4B8974

Fig. 12

≡ Helminthosporiumguareicola F. Stevens [as ‘Helmisporiumguareicolum’], Bot. Gaz. 65(3): 241, 1918.
≡ Pleurophragmiumguareicola (F. Stevens) S. Hughes, Can. J. Bot. 36: 797, 1958.
= Cladosporiumelegansvar.singaporense Sacc., Bull. Orto Bot. Regia Univ. Napoli 6: 60, 1921.
= Helminthosporiumflagellatum H.S. Yates [as ‘Helmisporium’], Philipp. J. Sci. (Bot.) 13: 383, 1918.
= Helminthosporiumspirotrichum Sacc. [as ‘Helmisporium’], Boll. Orto bot. 6: 61, 1921.

###### Description.

***Colonies*** effuse, dark brown to black, hairy. ***Hyphae*** superficial, branched, septate, 2–4 µm wide, pale olivaceous-brown, smooth. ***Conidiophores*** arising singly or in groups, as lateral branches on the hyphae, erect, sterile lower part straight or flexuous, upper fertile part in zigzag shape, septate, up to 400 µm long, 6–9 µm thick, brown to dark brown, paler towards the apex, more or less smooth, with numerous well-defined, dark conidial scars. ***Conidia*** solitary, broadly fusiform, truncate at the base, with 3 to 5 pseudosepta, (25–)35–52(–60) × (7–)8–10(–13) µm, 3.5–5 µm wide at the base, pale to dark brown or olivaceous-brown, smooth as seen by SEM.

###### Specimen examined.

On leaves of *Cyrtophyllumfragrans* (Gentianaceae), Singapore, 1921, Baker (IMI 49160, type of *Helminthosporiumspirotrichum*); on *Meliola* sp. on leaves of *Danielliathurifera* (Fabaceae), Sierra Leone, 1936, F.C. Deightonii M1267 (IMI 10010).

###### Illustrations.

This species was illustrated by Ellis (1968).

###### Known hosts and distribution.

On colonies of *Asteridiella* spp., *Irenopsis* spp. and *Meliola* spp. on living leaves of various plants in Bougainville Islands, Ghana, India, Indonesia, Malaysia, Philippines, Puerto Rico, Sabah, Sierra Leone, Solomon Islands and Uganda (Ellis 1968).

**Figure 12.**
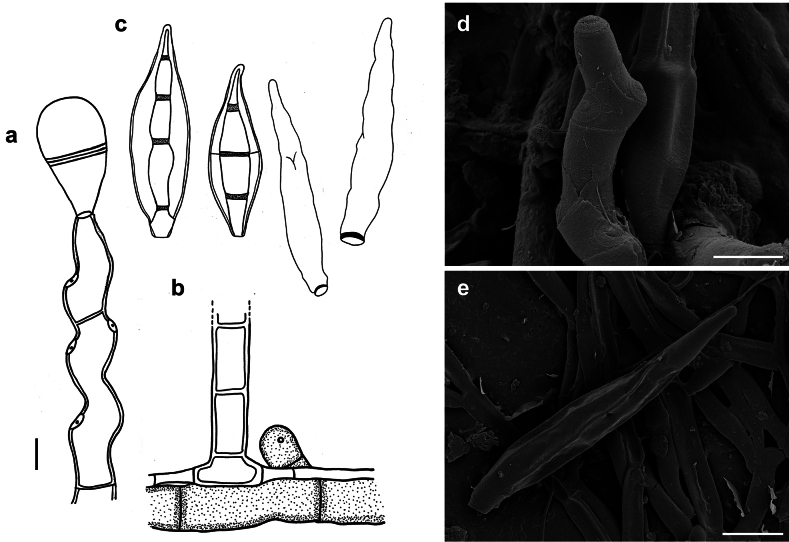
*Spiropesguareicola* (IMI 10010) **a** conidiophore with scars and a young conidium shown in optical section **b** base of a conidiophore growing on a hypha of *Meliola* sp. shown in optical section **c** conidia shown in optical section (two drawings on the left-hand side) and as seen by SEM (two drawings on the right-hand side) **d, e** as seen by SEM**d** zigzag-shaped conidiophore with scars **e** conidium. Scale bars: 5 μm (**a**–**c**); 8 μm (**d**); 10 μm (**e**).

###### Notes.

*Spiropesguareicola* is the type species of the genus *Spiropes* and it differs from other species of the genus by the presence of zigzag-shaped conidiophores in the fertile upper parts (Ellis 1968). *S.guareicola* presents smooth conidia, a feature that is only evident by SEM.

##### ﻿ Spiropes helleri

Taxon classificationFungiMeliolalesAscomycota

(F. Stevens) M.B. Ellis, Mycol. Pap. 114: 14, 1968

0054BD6E-FF41-5B49-B810-03F3D6AAFCF2

Fig. 13

≡ Helminthosporiumhelleri F. Stevens [as ‘Helmisporium], Bot. Gaz. 65(3): 242, 1918.
= Helminthosporiumleucosykes H.S. Yates [as ‘Helmisporiumleucosykeae’], Philipp. J. Sci., C, Bot. 13(6): 382, 1918.
= Helminthosporiummaculosum Sacc. [as ‘Helmisporium’], Atti Accad. Sci. Ven.-Trent.-Istr. 10: 91, 1919 [1917].
≡ Pleurophragmiummaculosum (Sacc.) S. Hughes, Can. J. Bot. 36: 797, 1958.

###### Description.

***Colonies*** effused, dark brown to black, hairy. ***Hyphae*** superficial, branched, septate, 1–3 µm wide, straw-coloured or pale brown, smooth. ***Conidiophores*** arising singly as terminal or lateral branches on the hyphae, erect, straight or flexuous, septate, up to 600 µm long, 5–8 µm wide, brown to dark brown, paler towards the apex, smooth, with scattered conidial scars. ***Conidia*** solitary, obclavate, frequently rostrate, 3(–4)–septate, (26–)36–43(–50) × (6–)7–10(–13) µm, 3–4 µm wide at the truncate base, pale brown to brown, verruculose. As seen by SEM, the ornamentation of the spores is clearly reticulated, with thin networks and no ridges.

###### Specimens examined.

On *Meliola* sp. on leaves of *Cupaniaguatemalensis* (Sapindaceae), Panama, Chiriquí Province, Botanical Garden of the Autonomous University of Chiriquí (UNACHI), 8°25'55"N, 82°27'03"W, 34 m a.s.l., 11 February 2020, M. A. Bermúdez-Cova, A. Sanjur MB92 (UCH15489, M); on *Meliola* sp. on living leaves of *Pterocarpussantalinoides* (Fabaceae), Benin, Atlantique, Attogon, Niaouli Forest, 6°44'40"N, 2°7'53"E, 72 m a.s.l., 20 September 2022, A. Krauß, A. Tabé, O. Koukol, N.S. Yorou, AK15 (M).

###### Additional specimens examined.

On Meliolales on living leaves of an undetermined plant, Gold Coast Colony, Banau, 1949, S.J. Hughes 1141 (IMI44564); on *Meliola* sp. on leaves of *Myrciadeflexa*, Puerto Rico, El Alto de la Bandera, F.L. Stevens 8268 (IMI9991, type of *Helminthosporiumhelleri*).

###### Illustrations.

This species was illustrated by Ellis (1968).

###### Known hosts and distribution.

On colonies of *Asteridiella* spp., *Irenopsis* spp. and *Meliola* spp. on living leaves of various plants in Ghana, Malaysia, New Caledonia, Philippines, Puerto Rico, Sabah, Sierra Leone and Uganda (Ellis 1968). *Spiropeshelleri* is reported here for the first time for Benin and for mainland America (Panama).

**Figure 13.**
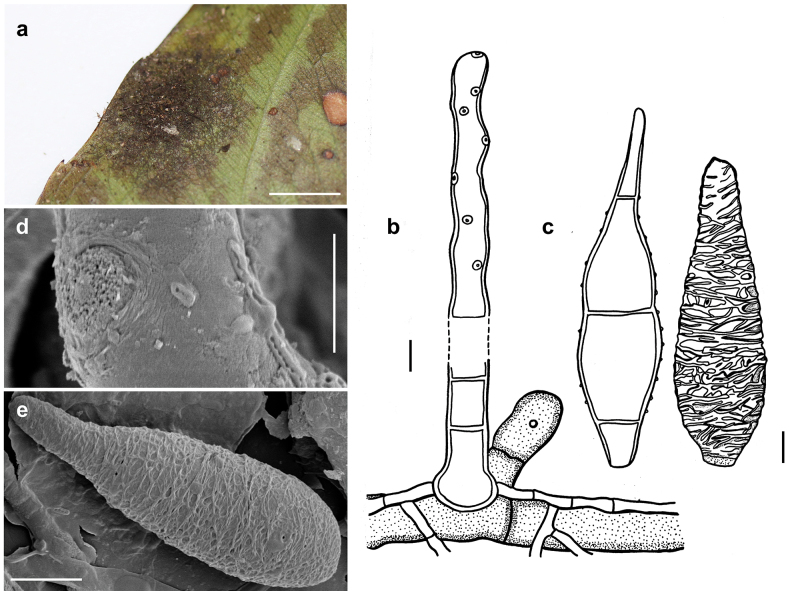
*Spiropeshelleri* (IMI130940) **a** superficial hyphae growing on a colony of *Meliola* sp. on a leaf of *Cupaniaguatemalensis***b** conidiophore growing on a hypha of *Meliola* sp. shown in optical section **c** conidia shown in optical section (drawing on the left-hand side) and as seen by SEM (drawing on the right-hand side) **d, e** as seen by SEM**d** part of a conidiophore with a scar **e** conidium. Scale bars: 1 mm (**a**); 5 μm (**b**); 6 μm (**c**); 4 μm (**b**); 5 μm (**c**).

###### Notes.

*Spiropeshelleri* is similar to *S.effusus*, *S.dorycarpus* and *S.leonensis* by the presence of obclavate to sometimes fusiform conidia, but differs from the first two by wider conidia (3.8–4.5 µm in *S.effusus* and 5–7 µm in *S.dorycarpus*) and from the last one by narrower ones (10–11µm).

##### ﻿ Spiropes intricatus

Taxon classificationFungiMeliolalesAscomycota

(Sacc.) M.B. Ellis, Mycol. Pap. 114: 9, 1968

12467E40-51F4-5DDC-98ED-6038B3B1B2D3

Fig. 14

≡ Brachysporiumintricatum Sacc., Atti Accad. scient. Veneto-trent.-istriana, Ser. 3, 10: 88, 1919.
= Spiropespirozynskii M.B. Ellis, Mycol. Pap. 114: 19, 1968. New synonym proposed in this study.

###### Description.

***Colonies*** effuse, straw-coloured, olive or olivaceous-brown, velvety or hairy. ***Hyphae*** superficial, branched, anastomosing, septate, 1–2 µm wide, pale olivaceous brown, smooth. ***Conidiophores*** arising singly or in groups, terminally or laterally from the hyphae, erect or ascending, straight or flexuous, septate, up to 900 µm long, 2–5 µm thick along most of their length, swollen to 4–9 µm towards the apex and in intercalary parts that produce conidia, pale olivaceous-brown to brown, reticulate as seen by SEM, with scattered cylindrical scars. ***Conidia*** solitary, straight or slightly curved, oblong-ellipsoid or obovate to clavate, truncate at the base, mostly 3–septate, (13–)16–23(–25) × (4.5–)6–8 µm, 1.5–3 µm wide at the base, the cells at each end of a conidium pale brown, intermediate cells brown, ornamented. As seen by SEM, the ornamentation of the spores is distinctly reticulated, with thin to thick networks that can form ridges.

###### Specimens examined.

On *Irenopsis* sp. on *Lindackeriabukobensis* (Achariaceae), Tanzania, Kigoma, 1964, K.A. Pirozynski M418 b&c (IMI 106645b-c, type of *Spiropespirozynskii*); on leaves of *Camelliadrupifera* (Theaceae), Nepal, Kathmandu, Godawari, 1986, U. Budathoki KU294 (IMI323287).

###### Illustrations.

This species was illustrated by Ellis (1968).

###### Known hosts and distribution.

On colonies of Meliolales on living leaves of various plants in Ghana, Philippines and Tanzania (Ellis 1968).

###### Notes.

**Figure 14.**
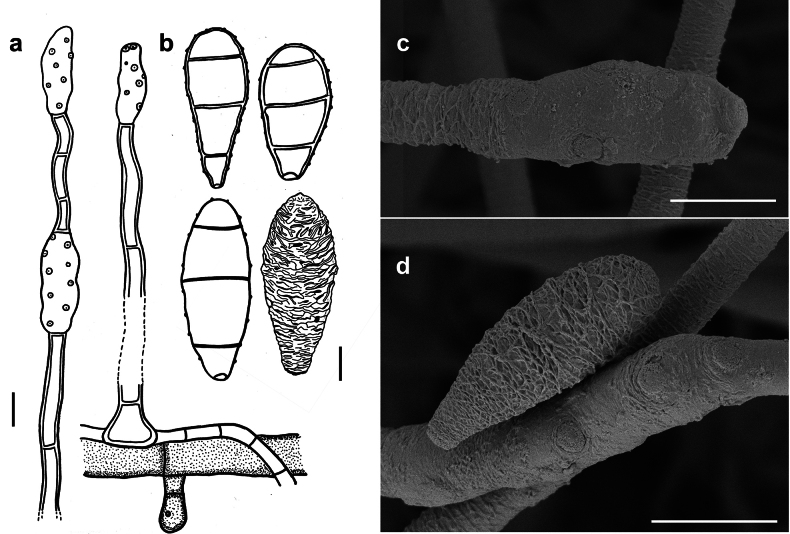
*Spiropesintricatus* (IMI 106645b-c) **a** conidiophores, growing on a hypha of *Irenopsis* sp., shown in optical section **b** conidia shown in optical section (the thickness of the wall is indicated only in the drawings on the upper row) and as seen by SEM (second row right) **c, d** as seen by SEM**c** conidiophore with scars **d** conidium. Scale bars: 5 μm (**a**); 3 μm (**b**); 7 μm (**c**); 8 μm (**c**).

**Figure 15.**
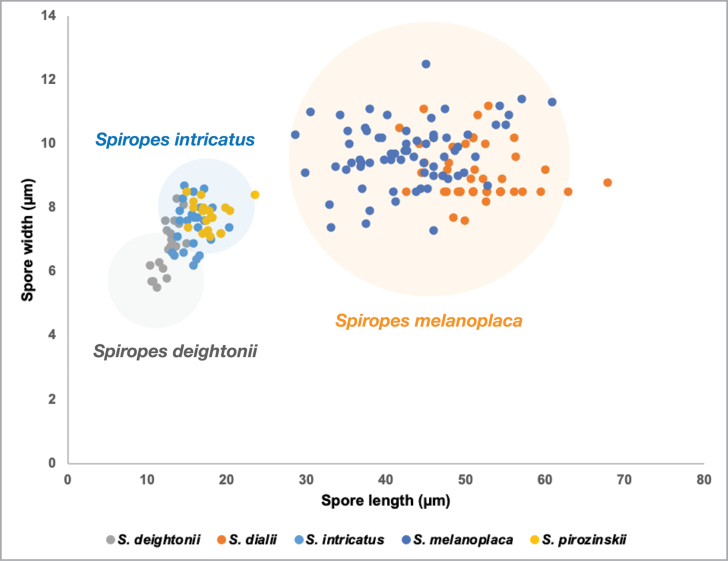
Scatter plot of spore size (width and length) of species of *Spiropes*.

*Spiropesintricatus* and *S.deightonii* are the only known species of the genus that present conidiophores that swell in the areas where conidia are formed (Figs 9, 14; Ellis (1968)). *Spiropesdeightonii* differs from *S.intricatus* by the presence of smaller conidia (12–14 µm long) that are more obovate or clavate rather than oblong-ellipsoid. The type specimen of *S.pirozynskii* (IMI 106645b-c) is morphologically similar to *S.intricatus*. Both species present oblong-ellipsoid conidia with a similar size range (Fig. 15). Therefore, we propose *S.pirozynskii* as a synonym of *S.intricatus*.

##### ﻿ Spiropes japonicus

Taxon classificationFungiMeliolalesAscomycota

(Henn.) M.B. Ellis, Mycol. Pap. 114: 22, 1968

BEECBAFF-C99C-5DDA-9066-D406E9D0B125

Fig. 16

≡ Podosporiumjaponicum Henn., Bot. Jb. 29: 152, 1900.
= Helminthosporiuminsigne Gaillard ex Sacc. [as ‘Helmisporium’], Atti Accad. Sci. Ven.-Trent.-Istr. 10: 89, 1917.

###### Description.

***Colonies*** effuse, amphigenous, sometimes dense, dark brown to black, with tightly packed hyphae that form large, erect, dark synnemata clearly visible under the stereomicroscope. ***Hyphae*** superficial, branched, septate, 1–4 µm wide, pale olivaceous-brown, smooth. ***Conidiophores*** tightly packed to form dark brown to blackish synnemata up to 1 mm high, spreading out at the apex and upper half of the synnemata; conidiophores individually flexuous or straight, thick-walled, septate, 6–8 µm thick, brown to dark brown at the base, paler towards the apex, smooth, with scattered cylindrical scars. ***Conidia*** solitary, fusiform to obclavate, with 4(–6) pseudosepta, (50–)67–80 × (7–)8–14 µm, 2–3 µm wide at the apex, 3–5 µm at the truncate base, pale brown to brown, striate.

**Figure 16.**
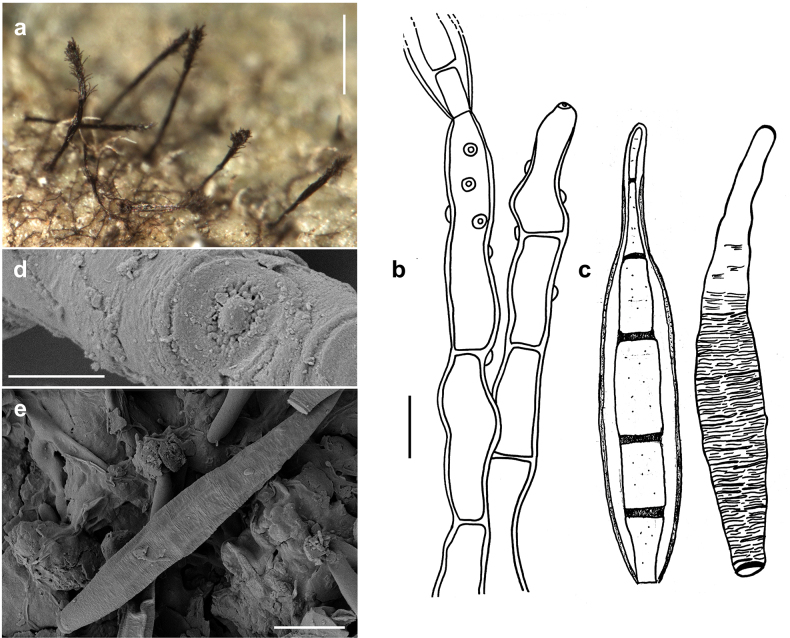
*Spiropesjaponicus* (MB120, 123) **a** synnemata growing on a colony of *Meliola* sp. **b** conidiophores with scars and a young conidium, shown in optical section **c** a conidium shown in optical section (drawing on the left) and as seen by SEM (drawing on the right) **d, e** as seen by SEM**d** conidiophore with a scar **e** conidium. Scale bars: 1 mm (**a**); 10 μm (**b, c**); 3 μm (**d**); 9 μm (**d**).

###### Specimens examined.

On *Meliola* sp. on living leaves of Asteraceae, Panama, Chiriquí Province, Boquerón District, Chuspa Hydroelectric, 8°32'20"N, 82°36'21"W, 281 m a.s.l., 6 March 2020, M. A. Bermúdez-Cova, A. Sanjur, S. Samaniego, MB120 (UCH15492); on *Meliola* sp. on living leaves of Fabaceae, Panama, Chiriquí Province, Bugaba District, area around Gariché River, 8°38'38.1"N, 82°41'19.6"W, 566 m a.s.l., 8 March 2020, M. A. Bermúdez-Cova, A. Sanjur, A. Villarreal, MB123 (UCH15493, M).

###### Additional specimens examined.

On *Ireninaentebbeensis* on *Alchorneahirtella* (Euphorbiaceae), Sierra Leone, 1939, Makump, M1774 (IMI 38813); on *Asteridiellaaucubae* on *Aucubajaponica* (Garryaceae), Japan, Ise, 1899, P. Hennings (IMI 130973, type of *Podosporiumjaponicum*).

###### Illustrations.

This species was illustrated by Ellis (1968).

###### Known hosts and distribution.

On colonies of Meliolales on living leaves of various plants in the Cook Islands, Japan, Malaysia, Papua New Guinea and Sierra Leone (Ellis 1968). *Spiropesjaponicus* is reported here for the first time for Panama.

###### Notes.

*Spiropesjaponicus* is the only known synnematous species of *Spiropes* that produces conidia with 4–6 pseudosepta, as well as synnemata that splay out at the apex and upper half (Ellis 1968).

##### ﻿ Spiropes leonensis

Taxon classificationFungiMeliolalesAscomycota

M.B. Ellis, Mycol. Pap. 114: 15, 1968

05040BF2-C3F4-5660-9D8E-1C572AE7B535

Fig. 17

###### Description.

***Colonies*** effuse, grey to dark blackish-brown, hairy. ***Hyphae*** superficial, branched, septate, 2–6 µm wide, pale brown, smooth. ***Conidiophores*** arising singly, as terminal and lateral branches on the hyphae, erect, straight or flexuous, septate, up to 700 µm long, 8–12 µm thick, sometimes swollen to 16–17 µm at the base, dark brown to dark blackish-brown, paler towards the apex, smooth, with scattered conidial scars. ***Conidia*** solitary, obclavate, rostrate, 3(–4)–septate, (38–)40–54(–63) × (8–)10–11(–13) µm, 4–6 µm wide at the truncate base, pale brown to brown, verruculose. As seen by SEM, the ornamentation of the spores is distinctly reticulated, with thin networks and no ridges. It was not possible to see the scars by SEM.

###### Specimen examined.

On *Meliolagarciniae* on leaves of *Pentadesmabutyracea* (Clusiaceae), Sierra Leone, Rokupr, 1951, F.C. Deighton M3920 (IMI 46589b, holotype); on *Meliolagarciniae* on *Pentadesmabutyracea*, Sierra Leone, near Rokupr, 1939, F.C. Deighton (IMI 9992a, type of *Spiropesleonensis*).

###### Illustrations.

This species was illustrated by Ellis (1968).

###### Known hosts and distribution.

On colonies of *Meliolagarciniae* on living leaves of *Pentadesmabutyracea* (Clusiaceae) in Sierra Leone (Ellis 1968).

**Figure 17.**
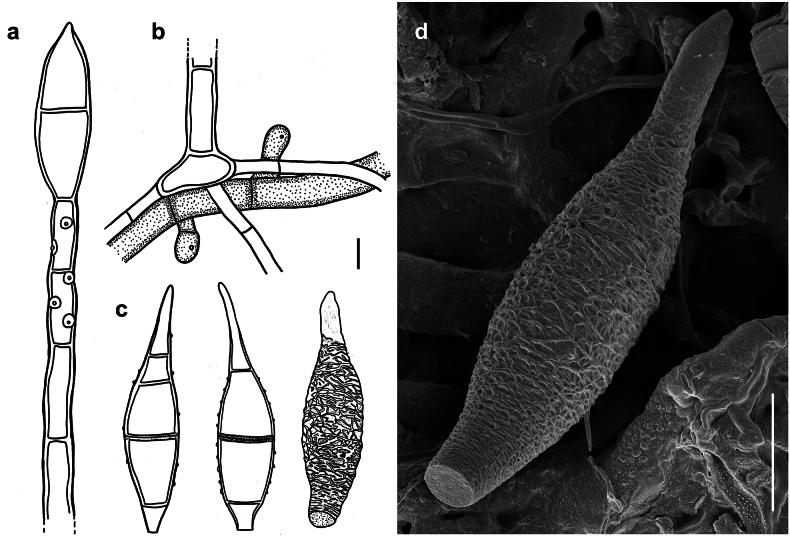
*Spiropesleonensis* (IMI 46589b) **a** conidiophore with scars and a young conidium, shown in optical section **b** part of a conidiophore growing on a hypha of *Meliola* sp., shown in optical section **c** conidia shown in optical section (first two drawings, from left to right) and as seen by SEM**d** conidium as seen by SEM. Scale bars: 8.5 μm (**a–c**); 7 μm (**d**).

###### Notes.

*Spiropesleonensis* is similar to *S.helleri* by the presence of rostrate, obclavate, 3–septate conidia (Ellis 1968). However, conidia in *S.helleri* are smaller (36–43 µm).

##### ﻿ Spiropes melanoplaca

Taxon classificationFungiMeliolalesAscomycota

(Berk. & M.A. Curtis) M.B. Ellis, Mycol. Pap. 114: 28, 1968

1F2A0DF2-79B7-5610-BB40-F8071D0D91A2

Fig. 18

= Arthrobotryummelanoplaca Berk. & M.A. Curtis, J. Linn. Soc. Bot. 10(46): 360, 1868.
≡ Podosporiummelanoplaca (Berk. & M.A. Curtis) Cif., Sydowia 9(1–6): 310, 1955.
= Podosporiumdialii Bat. [as ‘*dialiumii*’], Atas Inst. Micol. 1: 266, 1960. New synonym proposed in this study.
≡ Spiropesdialii (Bat.) M.B. Ellis, Mycol. Pap. 114: 27, 1968. New synonym proposed in this study.
= Arthrobotryumscoparium Henn., Hedwigia 43(6): 397, 1904. New synonym proposed in this study.

###### Description.

***Colonies*** effuse, dark brown to black, hairy, with tightly packed hyphae that form large, erect, dark synnemata clearly visible under the stereomicroscope. ***Hyphae*** superficial, branched, septate, 1.5–6 µm wide, pale olivaceous, smooth. ***Conidiophores*** tightly packed to form dark brown to blackish synnemata up to 1.5 mm high, spreading out at the apex, 20–80 µm thick, splaying out at the apex. Individual hyphae straight or flexuous, cylindrical, 2–6 µm thick along most of their length, 5–8 µm thick near the apex, with numerous small scars that may overlap like scales. As evident by SEM, the scales are produced by the peeling of the outer wall layers where the scars are located. ***Conidia*** straight or curved, fusiform to obclavate, 3-septate, (30–)40–52(–68) × (7–)9–11(–14) µm, with the two middle cells usually golden brown or brown, warty and the cells at each end paler. As seen by SEM, the ornamentation of the spores is distinctly reticulated, with thin to thick networks and no ridges.

###### Specimens examined.

On *Meliolamangiferae* on living leaves of *Mangiferaindica* (Anacardiaceae), Panama, Chiriquí Province, Los Algarrobos, 8°31'05"N, 82°25'25"W, 168 m a.s.l., 20 January 2020, M. A. Bermúdez-Cova, MB81; same fungal and plant host, Panama, Chiriquí Province, Universidad Autónoma de Chiriquí (UNACHI), 8°25'57"N, 82°27'02"W, 37 m a.s.l., 23 January 2020, M. A. Bermúdez-Cova, MB85 (UCH15487); same fungal and plant host, Panama, Chiriquí Province, Los Algarrobos, Majagua River Trail, 8°28'56"N, 82°24'47"W, 101 m a.s.l., 23 January 2020, M. A. Bermúdez-Cova, MB89 (UCH15488, M); same fungal and plant host, Panama, Chiriquí Province, Meseta de Chorcha, 8°24'19"N, 82°13'26"W, 94 m a.s.l., 16 February 2020, M. A. Bermúdez-Cova, A. Sanjur, MB101 (UCH); same fungal and plant host, Panama, Chiriquí Province, Boquerón District, Hidroeléctrica Chuspa, 8°33'37"N, 82°36'22"W, 331 m a.s.l., 6 March 2020, M. A. Bermúdez-Cova, A. Sanjur, S. Samaniego, MB119 (UCH15491); On *Meliola* sp. on living leaves of *Angylocalyxoligophyllus* (Fabaceae), Benin, Attogon, Niaouli, Niaouli Forest, 6°44'42"N, 2°7'50"E, 69 m a.s.l., 28 February 2022, M.A. Bermúdez-Cova, A. Tabé, I. Agonglo, O.P. Agbani, M. Piepenbring, N.S. Yorou, MB173 (M); on *Meliolamangiferae* on living leaves of *Mangiferaindica*, Benin, Attogon, Niaouli, Niaouli Forest, 6°44'44"N, 2°7'49"E, 65 m a.s.l., 28 February 2022, M.A. Bermúdez-Cova, A. Tabé, I. Agonglo, O.P. Agbani, M. Piepenbring, N.S. Yorou, MB180 (M).

###### Additional specimens examined.

On *Meliolamangiferae* on *Mangiferaindica*, Brunei, 1974, W.T.H. Peregrine (IMI189570a); on *Meliola* sp. on *Psychotria* sp. (Rubiaceae), Cuba, 1879, C. Wright (IMI 105348 and IMI 105349, syntypes of *Arthrobotryummelanoplaca*).

###### Illustrations.

This species was illustrated by Ellis (1968).

###### Known hosts and distribution.

On colonies of Meliolales, especially *Meliola* spp., on living leaves of various plants in Brazil, Cuba, China, Dominican Republic, Ghana, Guadalcanal, India, Malaysia, Peru, Philippines, Sierra Leone, Tanzania, Trinidad and Uganda (Ellis 1968; Zhao et al. 1996; Dubey and Moonnambeth 2013). *Spiropesmelanoplaca* is reported here for the first time for Benin and Panama.

**Figure 18.**
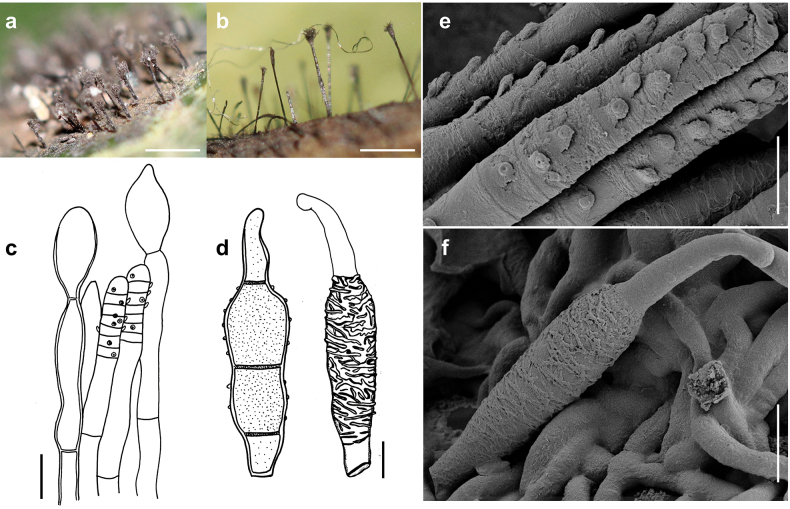
*Spiropesmelanoplaca* (MB81, MB119, IMI189570a) **a, b** synnemata growing on hyphae of *Meliolamangiferae* on living leaves of *Mangiferaindica***c** conidiophores with scars and young conidia shown in optical section. The thickness of the wall is only shown in the first conidiophore, from left to right **d** conidia, shown in optical section (left-hand drawing) and as seen by SEM (right-hand drawing) **e, f** as seen by SEM**e** parts of conidiophores with scars **f** conidium. Scale bars: 1.5 mm (**a**); b); 0.9 mm (**c**); 8 μm (**d**); 7 μm (**e**); 8 μm (**f**).

###### Notes.

According to Ellis (1968), the main difference between *Spiropesmelanoplaca* and *S.dialii* is the range of spore width, with *S.melanoplaca* having wider spores (9–14 µm wide) than *S.dialii* (7–9 µm wide). However, after revision of several specimens and herbarium material from both species, we noticed that the aspect of the colonies, morphological features (both as seen in LM and by SEM) are similar between the species and both species present conidia with a similar size range (Fig. 15). Therefore, we propose *S.dialii* as a synonym of *S.melanoplaca*.

##### ﻿ Spiropes palmetto

Taxon classificationFungiMeliolalesAscomycota

(W.R. Gerard) M.B. Ellis, Mycol. Pap. 114: 16, 1968

AABBD47C-EB40-5233-B327-BAC226D7CFBF

Fig. 19

≡ Helminthosporiumpalmetto W.R. Gerard, Grevillea 17(83): 68, 1889.
≡ Pleurophragmiumpalmetto (W.R. Gerard) S. Hughes, Can. J. Bot. 36: 778, 1958.

###### Description.

***Colonies*** effuse, dark brown to black, hairy. ***Hyphae*** superficial, branched, anastomosing, septate, 1–4 µm wide, pale olivaceous-brown, smooth. ***Conidiophores*** arising singly or in groups, as terminal and lateral branches on the hyphae, erect, straight or flexuous, septate, up to 400 µm long, 6–10 µm thick, dark brown, paler towards the apex, smooth, with scattered conidial scars. ***Conidia*** solitary, obclavate to fusiform, rostrate, with 2 septa delimiting a barrel-shaped central cell and often with an additional dark central pseudoseptum, (27–)30–46 × (7–)9–12(–15) µm, 3–5 µm wide at the truncate base, brown, middle cells pale brown, smooth as seen by LM and SEM.

###### Specimens examined.

On *Meliola* sp. on leaves of *Elaeisguineensis* (Arecaceae), Ghana, Apremodo, 1949, S.J. Hughes 534 (IMI 38617); on *Meliola* sp. on leaves of *Sabalpalmetto* (Arecaceae), U.S.A, Louisiana (IMI 10032, type of *Helminthosporiumpalmetto*).

###### Illustrations.

This species was illustrated by Ellis (1968).

###### Known hosts and distribution.

On colonies of *Irenopsis* spp. and *Meliola* spp. on living leaves of various plants in Ghana, Malaysia, New Zealand, Puerto Rico, Sierra Leone and the U.S.A. (Ellis 1968).

**Figure 19.**
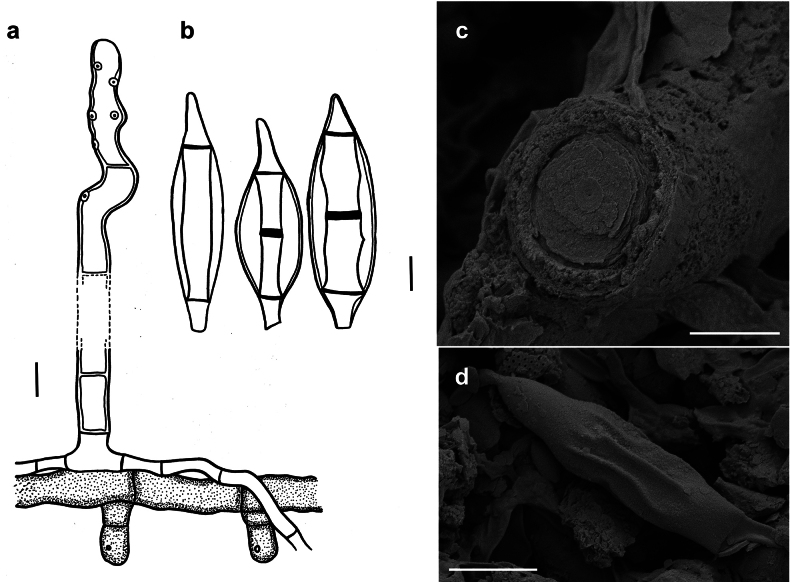
*Spiropespalmetto* (IMI 10032) **a** conidiophore growing on a hypha of *Meliola* sp., shown in optical section **b** conidia shown in optical section. The thickness of the walls is only shown in the two last drawings **c, d** as seen by SEM**c** part of a conidiophore with a scar **d** conidium. Scale bars: 7 μm (**a**); 5 μm (**b**); 6 μm (**c**); 7 μm (**d**).

###### Notes.

*Spiropespalmetto* can be easily recognised by the presence of conidia with two septa that delimit a barrel-shaped central cell and with a dark central pseudoseptum (Ellis 1968).

##### ﻿ Spiropes penicillium

Taxon classificationFungiMeliolalesAscomycota

(Speg.) M.B. Ellis, Mycol. Pap. 114: 23, 1968

225E7953-1A32-506E-9CDE-60A0E2F603A3

Fig. 20

≡ Podosporiumpenicillium Speg., Boln. Acad. nac. Cienc. Córdoba 11: 618, 1889.
≡ Arthrobotryumpenicillium (Speg.) F. Stevens, Bot. Gaz. 65: 238, 1918.
= Arthrobotryumstrychni Henn., Hedwigia 43: 397, 1904.
≡ Podosporiumstrychni (Henn.) Cif., Sydowia 9: 311, 1955.
= Arthrobotryumglabroides F. Stevens, Bot. Gaz. 65: 237, 1918.
≡ Podosporiumglabroides (F. Stevens) Cif., Sydowia 9: 309, 1955.

###### Description.

***Colonies*** effuse, yellowish to dark olivaceous-brown, velvety, with tightly packed hyphae that form large, erect, dark synnemata clearly visible under the stereomicroscope. A bright yellow pigment diffuses out when colonies are mounted in lactic acid or lacto-phenol. ***Hyphae*** superficial, branched, septate, 1–2 µm wide, yellowish, pale olive, smooth. ***Conidiophores*** tightly packed to form dark brown to blackish synnemata up to 650 µm long, 10–40 µm thick, often splaying out to a width of 100 µm at the apex. Individual hyphae straight or flexuous, cylindrical, 1–2 µm thick near the base, 2–3.5 µm thick near the apex, pale olivaceous-brown, smooth, with numerous small conidial scars. ***Conidia*** solitary, fusiform or occasionally almost cylindrical, mostly 3(–5)–septate, 16–23(–37) × (3–)3.5–5(–7) µm, tapering to about 1 µm at the apex and base, middle cells pale brown, the cells at each end paler, surface wrinkled or verruculose. As seen by SEM, the ornamentation of the spores is distinctly reticulated, with thin to thick networks that can form ridges-like structures.

###### Specimen examined.

On *Meliolacalva* on leaves of Lauraceae, Brasil, S. Paulo, Apiahy, 1881, J. Puiggari 1483 (IMI 131184, type of *Podosporiumpenicillium*); on *Meliola* sp. on leaves of *Oxyanthus* sp. (Rubiaceae), Sierra Leone, 1951, D.S. Rennis (IMI 51664).

###### Illustrations.

This species was illustrated by Ellis (1968).

###### Known hosts and distribution.

On colonies of *Asteridiella* spp. and *Meliola* spp. on living leaves of various plants in Brazil, Congo, Costa Rica, Ghana, Ivory Coast, Nigeria, Sierra Leone and Uganda (Ellis 1968).

**Figure 20.**
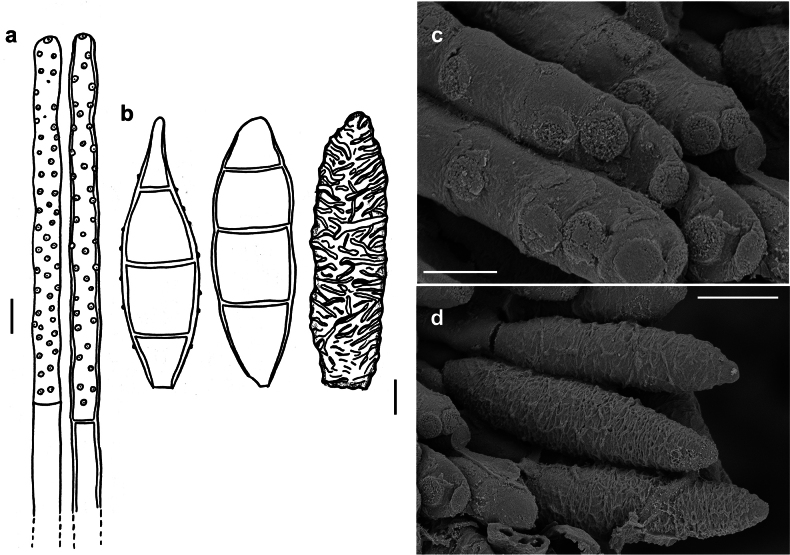
*Spiropespenicillium* (IMI 51664) **a** conidiophores with scars (the thickness of the wall is shown on the right-handed drawing) **b** conidia shown in optical section (first two left-hand drawings) and as seen by SEM**c, d** as seen by SEM**c** tips of conidiophores with scars **d** conidia. Scale bars: 5 μm (**a**); 2.5 μm (**b**); 3 μm (**c**); 5 μm (**d**).

###### Notes.

*Spiropespenicillium* is easily distinguishable from other known synnematous species of the genus *Spiropes* by the presence of fusiform to cylindrical conidia without rostra. In addition, a bright yellow pigment diffuses out of the cells when colonies are mounted in lactic acid or lacto-phenol (Ellis 1968).

### ﻿Key to species of *Atractilina* and *Spiropes* hyperparasitic on Meliolales

**Table:** 

1	Conidiophores synnematous	**2**
–	Conidiophores single or in groups	**7**
2	Synnemata straw-coloured to pale olivaceous; conidiophores with denticulate conidiogenous loci; pale multiseptate conidia	***A.parasitica***
–	Synnemata dark brown to black; conidiophores with cicatrised conidiogenous loci; conidia pigmented and multiseptate	**3**
3	Synnemata up to 400 μm long; conidia mostly crescent shape	***S.croissantiformis***
–	Synnemata longer, from 700 μm to 1.5 mm long; conidia fusiform to obclavate, occasionally cylindrical	**4**
4	Conidia fusiform to almost cylindrical; a yellow pigment diffuses out when colonies are mounted in lactic acid or lacto-phenol	***S.penicillium***
–	Conidia fusiform to obclavate; no yellow pigment	**5**
5	Conidia always 4–6 septate	***S.japonicus***
–	Conidia always 3–septate	**6**
6	Conidia 17–25 × 5–6.5 μm	***S.clavatus***
–	Conidia 40–52 × 9–11 μm	***S.melanoplaca***
7	Conidia with 3–6 pseudosepta	**8**
–	Conidia 1–3–septate	**10**
8	Conidiophores in larger groups; conidia with 3–6 (usually 4 or 5) pseudosepta	***S.capensis***
–	Conidiophores single or in small groups; conidia with 3–5 pseudosepta	**9**
9	Conidiophores with zigzag shape; conidia with 3–5 pseudosepta, fusiform to obclavate	***S.guareicola***
–	Conidiophores without zigzag shape; conidia with 3–4 pseudosepta, obovate	***S.fumosus***
10	Conidia 1–septate	**11**
–	Conidia 3–septate	**12**
11	Conidia obpyriform, verrucose	***S.armatellae***
–	Conidia obpyriform, smooth	***S.armatellicola***
12	Conidia oblong-ellipsoid	***S.intricatus***
–	Conidia of various shapes, not oblong-ellipsoid	**13**
13	Conidia obovate to clavate; conidiophores swollen towards the apex or in areas where conidia are produced	***S.deightonii***
–	Conidia ovate or fusiform to obclavate; conidiophores not swollen towards the apex or in areas where conidia are produced	**14**
14	Conidia obclavate; central cells barrel-shaped	**15**
–	Conidia ovate or fusiform to obclavate; without central barrel-shaped cells	**16**
15	Conidia with 3 true septa	***S.caribensis***
–	Conidia with 2 septa and a dark central pseudoseptum	***S.palmetto***
16	Conidia ovate	***S.carpolobiae***
–	Conidia fusiform to obclavate	**17**
17	Conidia 3–4.5 μm wide	***S.effusus***
–	Conidia wider	**18**
18	Conidia 17–25 μm long	***S.angylocalycis***
–	Conidia longer	**19**
19	Conidia 20–35 μm long	***S.dorycarpus***
–	Conidia longer	**20**
20	Conidia 36–48 μm long	***S.helleri***
–	Conidia 40–54 μm long	***S.leonensis***

**Figure 21.**
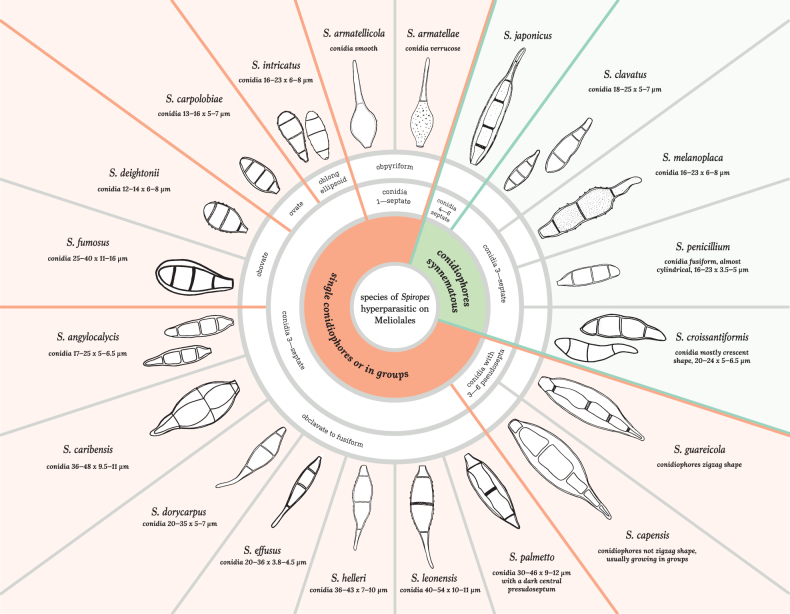
Visual key to known species of *Spiropes* hyperparasitic on Meliolales.

In Fig. 21, we propose a visual key to the known species of *Spiropes* hyperparasitic on Meliolales.

### ﻿Molecular position of species of *Atractilina* and *Spiropes*

In order to know the systematic positions of species of *Atractilina* and *Spiropes* hyperparasitic on Meliolales, new sequences of recently-collected specimens were obtained.

**Figure 22.**
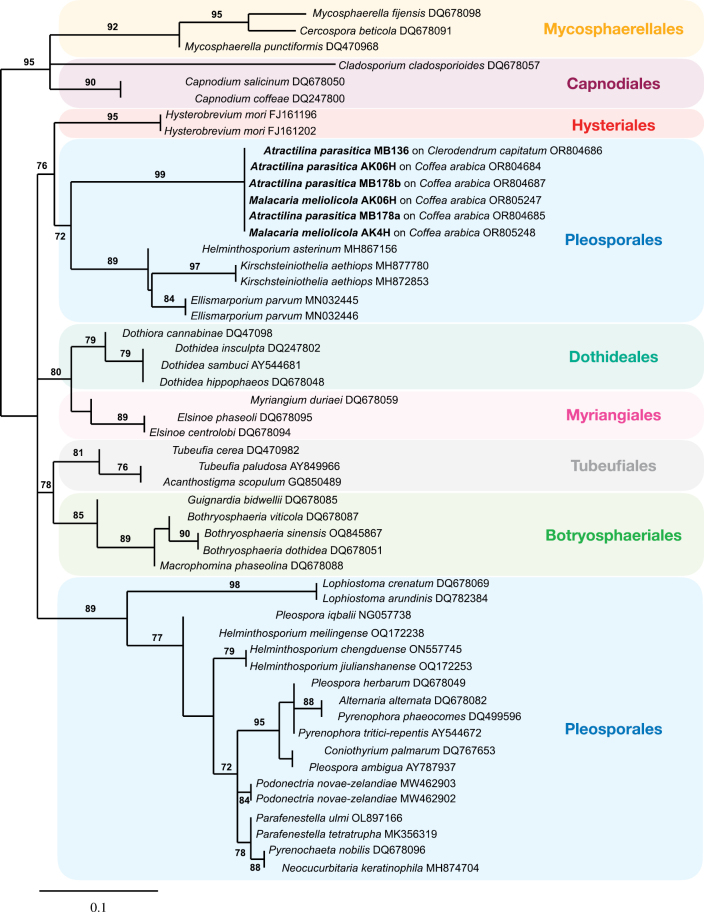
Phylogenetic tree inferred from a Maximum Likelihood analysis of nuc LSU rDNA sequences of members of the Dothideomycetes, including new sequences of *Atractilinaparasitica* and *Malacariameliolicola* (written with bold letters). The tree is rooted with sequences of species of the orders Capnodiales and Mycosphaerellales. Bootstrap values are indicated above the branches. Sequences downloaded from GenBank are given with accession numbers.

The BLAST query revealed that the nrLSU sequences of *Atractilinaparasitica* (specimens MB136 and MB178) show approximately 82% similarity with sequences of species of the Dothideomycetes, such as *Botryosphaeria* spp., *Helminthosporiumasterinum* Cooke, *Hysterobreviummori* (Schwein.) E. Boehm & C.L. Schoch and *Neoheleiosalincangensis* Mortimer, amongst others. In the tree inferred from the analysis of LSU sequences of 45 specimens of several orders of Dothideomycetes (Fig. 22), the sequences of *A.parasitica* are located in a well-supported clade that comprises species of Pleosporales, such as *Ellismarsporiumparvum* R.F. Castañeda & W.B. Kendr., *Kirschsteiniotheliaaethiops* (Sacc.) D. Hawksw. and *Helminthosporiumasterinum*. In addition, the sequences of *A.parasitica* cluster together in a strongly-supported clade with two DNA sequences we obtained from *Malacariameliolicola* (specimens AK4H and AK06H), a hyperparasitic perithecioid fungus that usually grows amongst the synnemata of *A.parasitica* on coffee leaves (see Bermúdez-Cova et al. (2023b) for the updated species description of *M.meliolicola*).

**Figure 23.**
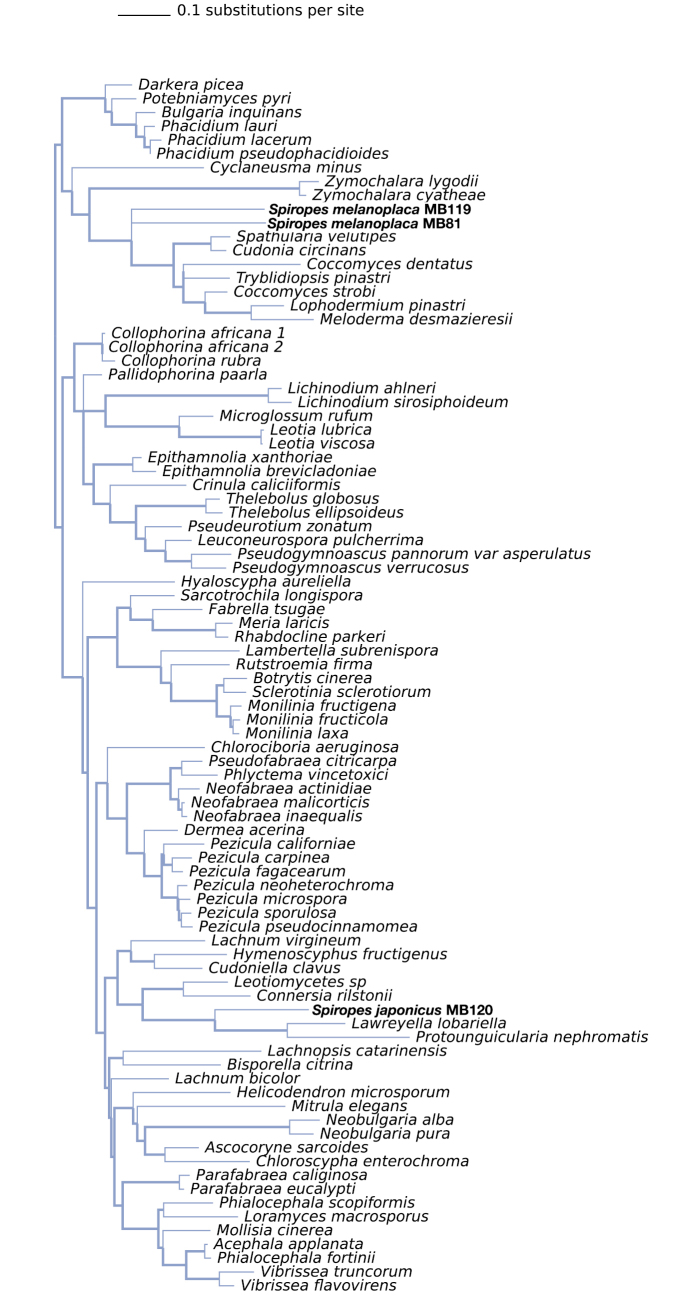
Placement of *Spiropesjaponicus* and *S.melanoplaca* on to Pezizomycotina reference tree version 2 in T-Bas. Only the Leotiomycetes clade is shown. The tree is the result of RAxML analysis of nuc ITS rDNA with 500 bootstraps replicates. For each node, the Maximum Likelihood bootstrap (≥ 70%) is presented as thick branches. Names of *Spiropes* species with newly-generated sequence data are written in bold.

As for species of *Spiropes*, the BLAST query revealed that the nrITS sequences of *Spiropesmelanoplaca* (specimens MB81 and MB119) and *Spiropesjaponicus* (specimen MB 120) are not closely related to each other (60% similarity) and show between 88 and 90% similarity with species of the Leotiomycetes, such as *Lophodermiumactinothyrium* Fuckel and *Hypoderma* spp., amongst others. Placement on to the Pezizomycotina tree version 2 in T-BAS confirmed that the newly-generated ITS sequences for the two species of *Spiropes* are placed in the Leotiomycetes (Fig. 23).

## ﻿Discussion

### ﻿*Atractilina* and *Spiropes*, two genera with heterogeneous species

Morphology-based identification of a species can be very difficult, especially amongst asexual or non-sporulating fungi (Jeewon et al. 2002; Promputtha et al. 2005, 2007). However, it continues to be an essential tool, especially for understudied groups of fungi and when DNA sequences are not available or scarce (Raja et al. 2017). The morphological analyses and the literature review of specimens of *Atractilina* and *Spiropes* revealed that both genera include highly heterogeneous species that are not necessarily congeneric with the type species of each genus.

The type species of *Atractilina*, *Atractilinacallicarpae* Dearn. & Barthol. (= *Atractilinaparasitica* (G. Winter) Deighton & Piroz.), has consistently true synnematous conidiophores, denticulate conidiogenous loci, pale pluriseptate (phragmoseptate) conidia and a hyperparasitic lifestyle (Deighton and Pirozynski 1972; Mel’nik and Braun 2013). Based on these characteristics, only three species of the genus are congeneric with *A.parasitica*, namely *A.alinae* Melnik & U. Braun, *A.biseptata* R.F. Castañeda and *A.calycini* T.K. Jana, S.N. Ghosh & A.K. Das (Castañeda-Ruiz 1986; Jana et al. 2006; Mel’nik and Braun 2013). The remaining two species present non-synnematous conidiophores and are probably not congeneric. *Atractilinaasterinae* (Hansf.) Deighton & Piroz. is a species hyperparasitic on Asterinales and presents single conidiophores and distoseptate conidia (Deighton and Pirozynski 1972). *Atractilinahymenaeae* Bat. & J.L. Bezerra (introduced as *Atractinahymenaeae* by the authors) is hyperparasitic on Meliolales, but also with non-synnematous conidiophores and conidia with a variable number of septa (Batista and Bezerra 1961). Therefore, we believe that both species have been incorrectly assigned to the genus *Atractilina*.

The description of *A.parasitica* introduced by Deighton and Pirozynski (1972) is very broad. As a result, specimens with significant morphological variations are grouped into a single species concept. For example, Chen and Tzean (2007) described a parasitic fungus from Taiwan growing on decaying leaves of *Liquidambar* sp. (Altingiaceae), with conidia that resemble those of *A.parasitica*. However, conidiophores of this fungus are non-synnematous and very short (less than 15 μm long), a feature that has never been reported before for *A.parasitica*. It is necessary to re-evaluate this and other identifications, to narrow the species concept of *A.parasitica*, as well as to complement it with DNA sequence data.

The DNA molecular analyses of the nrLSU rDNA region of the specimens of *A.parasitica* from Benin revealed that this species belongs to the Dothideomycetes. The Dothideomycetes are the largest and most diverse class of fungi and comprise species that exhibit a broad range of lifestyles, including saprotrophs, plant pathogens, mycoparasites and hyperparasites, as well as lichenised and lichenicolous fungi (Pem et al. 2021). They typically produce flask-like structures called pseudothecia, though apothecial, hysterothecial and cleistothecioid ascomata also exist (Hessen and Jahns 1973; Valenzuela-Lopez et al. 2019). Bitunicate asci are one of the diagnostic characters for Dothideomycetes taxonomy (Von Arx and Müller 1975; Pem et al. 2021). Asexual stages are frequent amongst pathogenic genera in the families *Cladosporiaceae*, *Mycopsphaerellaceae*, *Pleosporaceae* and *Tubeufiaceae*, amongst others (Hyde et al. 2013; Wanasinghe et al. 2018; Hongsanan et al. 2020). Conidiophores in these anamorphic species are usually solitary or in groups forming synnemata (Thambugala et al. 2017). The sequences of *A.parasitica* showed 98% similarity with sequences of *Malacariameliolicola* (Dothideomycetes, Ascomycota), a pseudothecioid hyperparasite that was found repeatedly amongst the synnemata of *A.parasitica* (Bermúdez-Cova et al. 2023b). The pseudothecia of *M.meliolicola* were also found to be growing without the presence of synnemata of *A.parasitica*. These colonies were used to extract the DNA of *M.meliolicola*. Therefore, the systematic position of *A.parasitica* in the Dothideomycetes and the anamorph-teleomorph connection between these two species are confirmed. This connection has been proposed in the past for these fungi on leaves of *Coffeaarabica* (Hansford 1941, 1946; Bermúdez-Cova et al. 2023b). Here, a DNA sequence from a specimen of *A.parasitica* on *Meliola* sp. on leaves of *Clerodendrumcapitatum* clustered with the aforementioned sequences in a highly-supported clade. The phylogenetic analysis of the nrLSU DNA locus showed that sequences of *A.parasitica* are located in a well-supported subclade together with other species of Pleosporales s.l., such as *Ellismarsporiumparvum* (Zhang et al. 2020). Many species of the Dothideomycetes, especially the asexual genera, are known to be polyphyletic (Schoch et al. 2009). To confirm the systematic hypothesis and to determine the placement of *A.parasitica* at family level, the use of multi-loci phylogenies is necessary in the future.

As for the genus *Spiropes*, the generic diagnosis given by Ellis (1968, 1971) allows us to include in this genus all species with cicatrised conidiogenous cells and conspicuous, flat and numerous scars, as well as pigmented, mostly obclavate phragmoconidia with 1–9 septa or pseudosepta. Seifert and Hughes (2000) proposed an amendment of this generic concept to also include species with dictyoconidia. As a result, *S.dictyosporus* is the only known species of the genus with muriform conidia. However, this morphological diagnosis allows for species with a wide range of types of conidiophores, conidiogenesis and conidia to be included in *Spiropes* (McTaggart et al. 2007). For example, the type species of the genus, *Spiropesguareicola* (F. Stevens) Cif., has distinctly sympodial-geniculate (zigzag-shaped) conidiophores, a character that is not present in any other known species of the genus (Ellis 1968). This species, in addition, presents distoseptate conidia, i.e. conidia with pseudosepta, a morphological feature that is present only in four species, namely *S.capensis*, *S.fumosus*, *S.guareicola* and *S.japonicus*. The remaining species of the genus present euseptate conidia (Ellis 1968, 1971). It is also possible to find a wide range of conidial shapes, such as obpyriform, obovate, ovate and oblong ellipsoid, to obclavate and fusiform (see the visual key to species of *Spiropes* in Fig. 21). Therefore, *Spiropes* is currently a genus with morphologically highly heterogeneous species and probably polyphyletic.

Identifying species of *Spiropes*, based on morphology alone, is not always easy. The most comprehensive key to species of the genus was proposed by Ellis (1968). However, this key is mainly based on the differences in the size range of the conidia of the species and, in some cases, these size differences are very subtle. Particular attention should be paid to herbarium specimens, as they may include immature or not well-preserved spores that can affect measurement results (Ordynets et al. 2021). We believe that other morphological characteristics that are not visible using standard light microscopy techniques should be considered when identifying species of *Spiropes* (e.g. Lutzoni et al. (2004)). Scanning electron Microscopy (SEM), for example, allowed us to observe for the first time the surface of the conidia of species of *Spiropes*. *Spiropesdialii* and *S.melanoplaca* were considered as different species by Ellis (1968). However, both species have overlapping spore-size ranges and the morphological analysis by SEM revealed that these species also have similar conidiogenesis and ornamentation patterns on conidia. This situation is similar for *S.intricatus* and *S.pirozynskii*. Therefore, we propose both groups of species as synonyms.

As for the molecular-based identification of species of *Spiropes*, there are currently no DNA sequences available in publicly-accessible databases. Species of the genus remain “incertae sedis” for many taxonomic ranks and it is difficult to assign new DNA sequences to species concepts (Bermúdez-Cova et al. 2022, 2023a). The DNA sequences generated for the first time in the context of this study suggest that species of *Spiropes* hyperparasitic on Meliolales may be polyphyletic in the Leotiomycetes. Fungi in the class Leotiomycetes are ecologically diverse and have been described as aquatic hyphomycetes, ectomycorrhizal parasites, endophytes, fungal parasites, mycorrhizal fungi, nematode-trapping fungi and plant-pathogens, amongst others (Wang et al. 2006a; Johnston et al. 2019). Many fungi have been suggested to belong to this class without any clear teleomorphic connection (Wang et al. 2006b). Up to date, no sexual stages have been linked to any species of *Spiropes* (Bermúdez-Cova et al. 2022). There is one genus with species morphologically similar to species of *Spiropes*, namely *Pseudospiropes* M.B. Ellis (Helotiales, Leotiomycetes; Ellis (1971)). Species of this genus differ from species of *Spiropes* by broadly enlarged, thickened, protuberant, strongly melanised conidiogenous loci and distoseptate conidia only (Castañeda-Ruiz et al. 2001; McTaggart et al. 2007). Species of *Pseudospiropes* have *Strossmayeria* Schulzer (Helotiales, Leotiomycetes) teleomorphs (Iturriaga and Korf 1984, 1990; Castañeda-Ruiz et al. 2001). Thus, there is a possibility that species of the genus *Spiropes* also belong to the Leotiomycetes. It is necessary to continue generating new DNA sequences from the different species of the genus in order to confirm this hypothesis, especially from those species that form part of mixed infections.

It is difficult to obtain molecular sequence data from hyperparasites especially because of the fact that they develop intermingled with the primary parasite and many other organisms and, as a result, no specific set of molecular methods has been developed to study hyperparasites (Bermúdez-Cova et al. 2022; Bermúdez-Cova et al. 2023a). As a consequence, isolating and sequencing hyperparasitic fungi is a challenging task. There is also a lack of sequences of hyperparasitic fungi in public. Therefore, the sequences obtained can be related to existing species concepts only based on morphology databases (Bermúdez-Cova et al. 2023b). For hyperparasitic fungi on Meliolales, for example, it is advised to obtain the same or very similar DNA sequences repeatedly from a given morphospecies in order to be sure to have the correct DNA sequence of that morphospecies. Despite many attempts, it was not possible to obtain DNA sequences from some of the species included in this study. However, this research provides valuable information that lays the foundation for future research on hyperparasites in Meliolales, highlighting the importance of field work paired with molecular for the study of challenging fungal groups. Further methodologies, such as metabarcoding, could represent another way to try to isolate the DNA of these organisms.

### ﻿The need for re-evaluation, resampling and epitypification

Applications of names based on morphological characteristics without DNA data is a challenge, resulting in the description of an excessive number of species or, in contrast, in the overlooking of cryptic species that can only be detected through molecular analyses (Hibbett et al. 2007; Crous et al. 2014; Jayasiri et al. 2015). The knowledge of morphological characteristics, however, is important to understand the evolution of fungal diversity (Raja et al. 2017). Instead of describing new species as part of *Atractilina* and *Spiropes*, a re-evaluation of the natural concepts of both genera is needed. Here we propose a list of actions that are necessary to carry out such a re-evaluation:

- Restudy the type species of each genus. When the type specimens of the type species are not in good condition or there is no more fungal material available for examination, it is necessary to recollect them. Epitypes and neotypes should be designated in these cases.
- After redefining the type species, all species belonging to the two genera need to be recollected, re-analysed morphologically and compared to the type species.
- The DNA of all existing species should be extracted, amplified and sequenced, in order to confirm or propose new concepts of genera and species. Multi-loci phylogenetic analyses are necessary to validate or propose new systematic hypotheses.

*Atractilina* and *Spiropes* are currently two repository genera of highly heterogeneous species and they may be split in the future, once species and genus concepts are validated respectively by morphology and molecular methods.

## Supplementary Material

XML Treatment for Atractilina parasitica

XML Treatment for Spiropes angylocalycis

XML Treatment for Spiropes armatellae

XML Treatment for Spiropes armatellicola

XML Treatment for Spiropes capensis

XML Treatment for Spiropes caribensis

XML Treatment for Spiropes carpolobiae

XML Treatment for Spiropes clavatus

XML Treatment for Spiropes croissantiformis

XML Treatment for Spiropes deightonii

XML Treatment for Spiropes dorycarpus

XML Treatment for Spiropes effusus

XML Treatment for Spiropes fumosus

XML Treatment for Spiropes guareicola

XML Treatment for Spiropes helleri

XML Treatment for Spiropes intricatus

XML Treatment for Spiropes japonicus

XML Treatment for Spiropes leonensis

XML Treatment for Spiropes melanoplaca

XML Treatment for Spiropes palmetto

XML Treatment for Spiropes penicillium

## ﻿Additional information

### Author ORCIDs

Miguel A. Bermúdez-Cova https://orcid.org/0000-0002-7712-7347

Tina A. Hofmann https://orcid.org/0000-0003-1124-402X

Nourou S. Yorou https://orcid.org/0000-0001-6997-811X

Meike Piepenbring https://orcid.org/0000-0002-7043-5769

### Data availability

All of the data that support the findings of this study are available in the main text or Supplementary Information.

## Supplementary materials

Supplementary material 1 Alignments and tree generated during the analysis of the DNA sequences of *Atractilinaparasitica*, *Malacariameliolicola* and other members of the Dothideomycetes Data type docx Explanation note Alignment is shown in NEXUS format. The tree is shown in Newick format. https://binary.pensoft.net/file/1024025This dataset is made available under the Open Database License (http://opendatacommons.org/licenses/odbl/1.0/). The Open Database License (ODbL) is a license agreement intended to allow users to freely share, modify, and use this Dataset while maintaining this same freedom for others, provided that the original source and author(s) are credited.Miguel A. Bermúdez-Cova, Tina A. Hofmann, Nourou S. Yorou, Meike Piepenbring
