# Supplementary material for: ﻿Systematic revision of species of Atractilina and Spiropes hyperparasitic on Meliolales (Ascomycota) in the tropics
Source: MycoKeys. 2024 Apr 11;103:167–213. doi: 10.3897/mycokeys.103.115799 (PMC11031638; doi:10.3897/mycokeys.103.115799)
Supplement: Supplementary material 1 — Alignments and tree generated during the analysis of the DNA sequences of Atractilinaparasitica, Malacariameliolicola and other members of the Dothideomycetes [file mycokeys-103-167-s001.docx]

**Supplementary materials**

**Supplementary material 1** Alignments and tree generated during the analysis of the DNA sequences of *Atractilina parasitica*, *Malacaria meliolicola* and other members of the Dothideomycetes. Alignment is shown in NEXUS format. The tree is shown in Newick format.

**Alignment**

#NEXUS

BEGIN TAXA;

DIMENSIONS ntax=52;

TAXLABELS AtM36_Cler At.para.AK At.para.MB Mal.fl.AK0 AtM7b_Coff Mal.fl.AK4 Cl.cl.isol K.ae.MH877 K.ae.MH872 He.as.MH86 El.parv.MN MN032446.1 Ca.sal.DQ6 Ca.co.DQ24 Myc.fi.DQ6 Myc.pu.DQ4 Ce.be.DQ67 T.palu.AY8 T.cer.DQ47 Ac.sc.GQ85 Do.ca.DQ47 Dd.in.DQ24 Dd.sam.AY5 Dd.hi.DQ67 Myr.du.DQ6 Es.phs.DQ6 Es.cen.DQ6 G.bi.DQ678 B.v.DQ6780 Mac.phsn.D B.si.OQ845 B.do.DQ678 Hy.mo.FJ16 Hy196 He.me.OQ17 He.ch.ON55 He.j.OQ172 L.cr.DQ678 L.ar.DQ782 Pl.iq.NG_0 Po2.1 Po.nv.zela Co.palm.DQ Pyc.nb.DQ6 N.k.MH8747 Pa.u.isola Pa.te.MK35 Pl.am.AY78 Al.al.DQ67 Pl.he.DQ67 Pyp.tr.rep Pyp.phe.DQ;

END;

BEGIN CHARACTERS;

DIMENSIONS nchar=2447;

FORMAT datatype=DNA gap=-;

MATRIX

AtM36_Cler --------------------------------------------------------------------------------------------------------------------------------------------------------------------------------------------------------------------------------------------------------------------------------------------------------------------------------------------------------------------------------------------------------------------------------------------------------------------------------------------------------------------------------------------------------------------------------------------------------------------------------------------------------------------------------------------------------------------------------------------------------------------------------------------------------------------------------------------------------------------------------------------------A-GAATCCCGT--GGAGGCGCG-GGCCGGACGCGTGTTAGGCTCCTTCGACGAGTCGAGTTGTTTGGGAATGCAGCTCTAAGCGTGAGGTAAATTCCTTCCAAGGCTAWATATCKGCTGGAGACCGATAGCGCRCAAGYAGAGTGATCGAAAGATGAAAAGCACTTTG-AAAAGAGAGTCAAACAGCACGTGAAATTGTTGAAAGGGAAGCGCTGGCGACCAGACGYGYGC-CGCCAGATCACCCAGCAGTC--TG-CTGGGGTACTCTTGGCGGCACAGGCCCGCATCGSTTGGGGCGGCTGGACAAAGGCCGTGGGAATGTAGCTCTTC--------GGAGTGTTATAGCCCGCGGTGTAATGCGGCCAGCCTCGACCGAGGTCTGCG-AT-TC--GTCTCGGATGCGGGCGTAATGGTCGTCAGCGGCCCGTCTTGAAACAC-GGATTGGTAAGTCCTCCAGAGTAAAAGAGAGAATGAGCTAACGAAAACRGAGGAGTCTAACATCTACGCGAGTGTTTGGGCGTCAAACCCGAGCGCGAAA--TGAAAGTG-AACGCAGGCGAGAS-CATT------GCGCACCGCCG-ACCGATCCGG--AAGTTTACGGAT-GGATTTGAGTACG-AGCGTGG-CTGTTGGGA-CCCGAAAGATGGTGAACTATGCGTGAATAGGGGGAAGCCARGGG-AAACCTTGGTGGAGGCTCGCAGCGG--------------------------------------------------------------TTCTGAC-GGGCA--------------------------------------------------------------------------------AATCGATCGTCAA--------------ATTTGCGTATA-GGGGCGAAAGACTAATC----------------------------------------------------------------------------------------------------------GAACCAT--------------------------------CTAGTAGCTGGTTCCTGCCGAAGTTCYCCC------------------------------------------------------------------------------------------------------------------------------------------------------------------------------------------------------------------------------------------------------------------------------------------------------------------------------------------------------------------------------------------------------------------------------------------------------------------------------------------

At.para.AK ----------------------------------------------------------------------------------------------------------------------------------------------------------------------------------------------------------------------------------------------------------------------------------------------------------------------------------------------------------------------------------------------------------------------------------------------------------------------------------------------------------------------------------------------------------------------------------------------------------------------------------------------------------------------------------------------------------------------------------------AACAGGGCTTGCCCTAGTAAC--GGCGAGTGAAGCGGCAAGAGCTCAGATTTGAAAGCGGCAGMCW--------KGTCGCGTTGTAATCTGCAGAGGATGCCTAGCGGAAGA-CCGCGCCTCAAGTCTCCTGGAACGGAGCGTCATGGAGGGTGAGGAATCGCCGTGGTAGGCGCG-CGCCGGACGCGTGTTAGGCTCCTTCGACGAGTCGAGTTGTTTGGGAATGCAGCTCTAAGCGGGAGGTAAATTCCTTCCAAGKCTAAMTATCGGCTGGAGACCGATAGCGCACAAGYAGAGTGATCGAAAGATGAAAAGCACTTTG-AAAAGAGAGTCAAACAGCACGTGAAATTGTTGAAAGGGAAGCGCTGGCGACCAGACGTGYGC-CGCCAGATCACCCAGCAGTC--TG-CTGGGGTACTCTTGGCGGCGCAGGCCCGCATCGGTTGGGGCGGCTGGACAAAGGCCGTGGGAATGTAGCTCCTC--------GGAGYGTTATAGCCCGCGGTGCAATGCGGCCAGCCTCGACCGAGGTCTGCG-AT-TC--GTCTCGGATGCGGGCGTAATGGTCGTCAGCGGCCCGTCTTGAAACAC-GG--------------------------------------------ATTGAGGAGTCTAACATCTACGCGAGTGTTTGGGCGTCAAACCCGAGCGCGAAAATGAAAAGTGAAACGCAGGCGAGAMSCATT------GCGCACCGCCGAACCGATCCCGGAARGTTTACGGATGGGATTTGAGTACGAAGCGTGGSYTGTTGGGAMCCCGAAAGATGGTGAACTATGCGKGAATAGGGTGAAGCCAAGGGAAAACCTTGGTGGAGGCTCGCAGCGG--------------------------------------------------------------TTCTGACGGKGCA--------------------------------------------------------------------------------AATCGATCGTCAA--------------ATTTGCGTATAGGGGGCGAAAGACTAATC----------------------------------------------------------------------------------------------------------GAACCAT--------------------------------CTAGTAGCTGGTTCCTGCCGAGTY------------------------------------------------------------------------------------------------------------------------------------------------------------------------------------------------------------------------------------------------------------------------------------------------------------------------------------------------------------------------------------------------------------------------------------------------------------------------------------------------

At.para.MB -------------------------------------------------------------------------------------------------------------------------------------------------------------------------------------------------------------------------------------------------------------------------------------------------------------------------------------------------------------------------------------------------------------------------------------------------------------------------------------------------------------------------------------------------------------------------------------------------------------------------------------------------------------------------------------------------------------------------------------------------------------------------------------------------------------------------------------GCGGACG--------------------------------------------------------------------------------------CGTSTTAGGCTCCTTCGACGAGTCGAGTTGTTTGGGAATGCAGCTCTMAGCGGGAGGTAAATTCCTTCCAAGGCTAAATATCGGCTGGAGACCGATAGCGCACAAGYAGAGTGATCGAAAGATGAAAAGCACTTTG-AAAAGAGAGTCAAACAGCACGTGAAATTGTTGAAAGGGAAGCGCTGGCGACCAGACGTGTGC-CGCCAGATCACCCAGCAGTC--TG-CTGGGSTACTCTTGKCGGCGCAGGCCCGCATCGGTTGGGGCGGCTGGACAAAGGCCGTGGGAATGTAGCTCCTC--------GGAGYGTTATAGCCCGCGGTGCAATGCGGCCAGCCTCGACCGAGGTCTGCG-AT-TC--GTCTCGGATGCGGGCGTAATGGTCGTCAGCGGCCCGTCTTGAAACAC-GG--------------------------------------------ATTGAGGAGTCTAACATCTACGCGAGTGTTTGGGCGTCAAACCCGAGCGCGAAA--TGAAAGTG-AACGCAGGCGAGAG-CATT------GCGCACCGCCG-ACCGATCCGG--AAGTTTACGGAT-GGATTTGAGTACG-AGCGTGG-CTGTTGGGA-CCCGAAAGATGGTGAACTATGCGTGAATAGGGTGAAGCCAAGGG-AAACCTTGATGGAGGCTCGCAGCGG--------------------------------------------------------------TTCTGAC-GTGCA--------------------------------------------------------------------------------AATCGATCGTCAA--------------ATTTGCGTATA-GGGGCGAAAGACTAATC----------------------------------------------------------------------------------------------------------GAACCAT--------------------------------CTAGTAGCTGGTTCCTGCCGAAGTSYCCCTCAGRAAATG---------------------------------------------------------------------------------------------------------------------------------------------------------------------------------------------------------------------------------------------------------------------------------------------------------------------------------------------------------------------------------------------------------------------------------------------------------------------------------

Mal.fl.AK0 ----------------------------------------------------------------------------------------------------------------------------------------------------------------------------------------------------------------------------------------------------------------------------------------------------------------------------------------------------------------------------------------------------------------------------------------------------------------------------------------------------------------------------------------------------------------------------------------------------------------------------------------------------------------------------------------------------------------------------------------------------------------------------------------------------------------AWKGTCGCGTTGTAATCTGCAGAGGATGCCTAGCGGAAGA-CCGCGCCTCAAGTCTCCTGGAACGGAGCGTCATGGAGGGTGA-GAATCCCGT--GGAGGCGCG-GGCCGGACGCGTGTTAGGCTCCTTCGACGAGTCGAGTTGTTTGGGAATGCAGCTCTAAGCGGGAGGTAAATTCCTTCCAAGGCTAAATATCGGCTGGAGACCGATAGCGCACAAGTAGAGTGATCGAAAGATGAAAAGCACTTTG-AAAAGAGAGYCAAACAGCACGTGAAATTGTTGAAAGGGAAGCGCTGGCGACCAGACGTGTGC-CGCCAGATCACCCAGCAGTC--TG-CTGGGGTACTCTTGGCGGCGCAGGCCCGCATCGGTTGGGGCGGCTGGACAAAGGCCGTGGGAATGTAGCTCCTC--------GGAGTGTTATAGCCCGCGGTGCAATGCGGCCAGCCTCGACCGAGGTCTGCG-AT-TC--GTCTCGGATGCGGGCGTAATGGTCGTCAGCGGCCCGTCTTGAAACAC-GG--------------------------------------------ATTGAGGAGTCTAACATCTACGCGAGTGTTTGGGCGTCAAACCCGAGCGCGAAA--TGAAAGTG-AACGCAGGCGAGAS-CATT------GCGCACCGCCG-ACCGATCCGG--AAGTTTACGGAT-GGATTTGAGTACG-AGCGTGG-CTGTTGGGA-CCCGAAAGATGGWGAACTATGCGTGAATAGGGTGAAGCCAAGGG-AAACCTTGGTGGAGGCTCGCAGCGS--------------------------------------------------------------TTCTGAC-GTGCA--------------------------------------------------------------------------------AATCGATCGTCAA--------------ATTTGCGTATA-GGKGCGAAAGACTAATC----------------------------------------------------------------------------------------------------------GAACCAT--------------------------------CTAGTAGCTGGTTCCTGCCGAKTT------------------------------------------------------------------------------------------------------------------------------------------------------------------------------------------------------------------------------------------------------------------------------------------------------------------------------------------------------------------------------------------------------------------------------------------------------------------------------------------------

AtM7b_Coff -----------------------------------------------------------------------------------------------------------------------------------------------------------------------------------------------------------------------------------------------------------------------------------------------------------------------------------------------------------------------------------------------------------------------------------------------------------------------------------------------------------------------------------------------------------------------------------------------------------------------------------------------------------------------------------------------------------------------------------------------------------------------------------------------------------------------CGTTGTAATCTGCAGAGGATGCCTAGCGGAAGA-CCGCGCCTCAAGTCTCCTGGAACGGAGCGTCATGGAGGGTGA-GAATCCCGT--GGAGGCGCG-GGCCGGACGCGTGTTAGGCTCCTTCGACGAGTCGAGTTGTTTGGGAATGCAGCTCTAAGCGGGAGGTAAATTCCTTCCAAGGCTAAATATCGGCTGGAGACCGATAGCGCACAAGTAGAGTGATCGAAAGATGAAAAGCACTTTG-AAAAGAGAGTCAAACAGCACGTGAAATTGTTGAAAGGGAAGCGCTGGCGACCAGACGTGTGC-CGCCAGATCACCCAGCAGTC--TG-CTGGGGTACTCTTGGCGGCGCAGGCCCGCATCGGTTGGGGCGGCTGGACAAAGGCCGTGGGAATGTAGCTCCTC--------GGAGTGTTATAGCCCGCGGTGCAATGCGGCCAGCCTCGACCGAGGTCTGCG-AT-TC--GTCTCGGATGCGGGCGTAATGGTCGTCAGCGGCCCGTCTTGAAACAC-GG--------------------------------------------ATTGAGGAGTCTAACATCTACGCGAGTGTTTGGGCGTCAAACCCGAGCGCGAAA--TGAAAGTG-AACGCAGGCGAGAG-CATT------GCGCACCGCCG-ACCGATCCGG--AAGTTTACGGAT-GGATTTGAGTACG-AGCGTGG-CTGTTGGGA-CCCGAAAGATGGTGAACTATGCGTGAATAGGGTGAAGCCAAGGG-AAACCTTGGTGGAGGCTCGCAGCGG--------------------------------------------------------------TTCTGAC-GTGCA--------------------------------------------------------------------------------AATCGATCGTCAA--------------ATTTGCGTATA-GGGGCGAAAGACTAATC----------------------------------------------------------------------------------------------------------GAACCAT--------------------------------CTAGTAGCTGGTTCCTGCCGAGTCCCCC--------------------------------------------------------------------------------------------------------------------------------------------------------------------------------------------------------------------------------------------------------------------------------------------------------------------------------------------------------------------------------------------------------------------------------------------------------------------------------------------

Mal.fl.AK4 -------------------------------------------------------------------------------------------------------------------------------------------------------------------------------------------------------------------------------------------------------------------------------------------------------------------------------------------------------------------------------------------------------------------------------------------------------------------------------------------------------------------------------------------------------------------------------------------------------------------------------------------------------------------------------------------------------------------------------------------------------------------------------------------MGSAA------------------WGTCGCGTTGTAATCTGCAGAGGATGCCTAGCGGAAGA-CCGCGCCTCAAGTCTCCTGGAACGGAGCGTCATGGAGGGTGA-GAATCCCGT--GGAGGCGCG-GGCCGGACGCGTGTTAGGCTCCTTCGACGAGTCGAGTTGTTTGGGAATGCAGCTCTAAGCGGGAGGTAAATTCCTTCCAAGGCTAAATATCGGCTGGAGACCGATAGCGCACAAGTAGAGTGATCGAAAGATGAAAAGCACTTTG-AAAAGAGAGTCAAACAGCACGTGAAATTGTTGAAAGGGAAGCGCTGGCGACCAGACGTGTGC-CGCCAGATCACCCAGCAGTC--TG-CTGGGGTACTCTTGGCGGCGCAGGCCCGCATCGGTTGGGGCGGCTGGACAAAGGCCGTGGGAATGTAGCTCCTC--------GGAGTGTTATAGCCCGCGGTGCAATGCGGCCAGCCTCGACCGAGGTCTGCG-AT-TC--GTCTCGGATGCGGGCGTAATGGTCGTCAGCGGCCCGTCTTGAAACAC-GG--------------------------------------------ATTGAGGAGTCTAACATCTACGCGAGTGTTTGGGCGTCAAACCCGAGCGCGAAA--TGAAAGTG-AACGCAGGCGAGAG-CATT------GCGCACCGCCG-ACCGATCCGG--AAGTTTACGGAT-GGATTTGAGTACG-AGCGTGG-CTGTTGGGA-CCCGAAAGATGGTGAACTATGCGTGAATAGGGTGAAGCCAAGGG-AAACCTTGGTGGAGGCTCGCAGCGG--------------------------------------------------------------TTCTGAC-GTGCA--------------------------------------------------------------------------------AATCGATCGTCAA--------------ATTTGCGTATA-GGGGCGAAAGACTAATC----------------------------------------------------------------------------------------------------------GAACCAT--------------------------------CTAGTAGCTGGTTCCTGCCGAGTCT-----------------------------------------------------------------------------------------------------------------------------------------------------------------------------------------------------------------------------------------------------------------------------------------------------------------------------------------------------------------------------------------------------------------------------------------------------------------------------------------------

Cl.cl.isol -----------------------------------------------------------------------------------------------------------------------------------------------------------------------------------------------------------------------------------------------------------------------------------------------------------------------------------------------------------------------------------------------------------------------------------------------------------CTTCGTCACGGGCCTCCACGCCTGCCTACTCGCTAGGGCATCGTTTCTACCCTAGCGGCGAGGTATTGGTAGTACGCTTGAGCGCCATCCATTTTCAGGGCTAGTTCATTCGGCAGGTGAGTTGTTACACACTCCTTAGCGGATTCCGACTTCCATGGCCACCGTCCTGCTGTCTAGATGAACTAACACCTTTTGTGGTGTCTGATAAGCGTACATTCCGGCACCTTAACCTCGCGTTCGGTTCATCCCGCATCGCCAGTTCTGCTTACCAAAGGGATTGCTCTAGTAAC--GGCGAGTGAAGCAGCAATAGCTCAAATTTGAAATCTGGCGTCTTCGAC---GTCCGAGTTGTAATTTGTAGAGGATGCTTCTGAGTGGC-CACCGACCTAAGTTCCTNGGAACAGGACGTCATAGAGGGTGA-GANTCCCGTATGCGGTCGGA-ANGNCGCTCTATACGTAGCTCCTTCGNCGAGTCGAGTTGTTNGGGACCCCAGCTCTAAATGNNAGGTAAATTTCTTCTAAAGCTAAATNNNNGCCAGAGACCGNNNNCGCACAAGTAGAGTGATCGAAAGATGAAAAGCACTTTG-GAAAGAGAGTTAANANGCACGTGAAATTGTTAAAAGGGAAGNGATNGCAACCAGACTTNCTCGCGG-TGTTNCGCCGNTCTTC--TGACCGGTCTACTNNNCGCGTTGCAGGCCNGCATCGTCTGGTGNCGCTGGATAA-GACTTGAGGAATGTAGCTCCCTC------GGGAGTGNTATAGCCTCTTGTG--ACGCAGCGAGCNCCGGNCGAGNTCCGCG-CT-TC--GCCTAGCATGCCGGCGTAATGNTCGTANTCCGCCCGTATTGAAACAC-GG--------------------------------------------ACCAAGGAGTCTAACATTTATGCGAGTGTGCGGGTGTCAAACCTCTACGCGTAA--TGAGCGTG-AACGGAGGTGAGAACCCCA----AGGGGCATCATCG-ACCGATCCTG--ATGTCATCGCAT-GGATTTGAGTAAG-AGCATAG-CTGTTTGGA-CCCGAAAGATGGTGAACTATGCCTGAATAGGGTGAAGCCAGAGG-AAACCTATGTGGAGGCTCGCATTAG--------------------------------------------------------------TTCTGAC-GTGCA--------------------------------------------------------------------------------AATCGATCGTCCC--------------ATTTGGGTATA-GNNNCGAAAGACTAATC----------------------------------------------------------------------------------------------------------GAACCAT--------------------------------CTAGTAGCTGGTTCCTGCCGAACTTTCCCTCAGGATAGCAGTTACGTTTTCAGTTTTATGAGGTAAAGCGAATGATTAGAGGCCTTGGGGTTGAAACAACCTTAACCTATTCTCAAACTTTAAATATGTAAGAAGTCC-TTGTTACTTAGTTGAACGTGGACATTTGAATGTATCGTTACTAGTGGGCCATC------------------------------------------------------------------------------------------------------------------------------------------------------------------------------------------------------------------------------------------------------------------------------------------------------------------------

K.ae.MH877 ---------------------------------------------------------------------------------------------------------------------------------------------------------------------------------------------------------------------------------------------------------------------------------------------------------------------------------------------------------------------------------------------------------------------------------------------------------------------------------------------------------------------------------------------------------------------------------------------------------------------------------------------------------------GCATATCAATAAGCGGAGGAAAAGAAA--CCAAC------------------------------------------AGGGATTGCCCTAGTAAC--GGCGAGTGAAGCGGCAACAGCCCAAATTTGAAATCTGGCCCCCTGGG----GTCCGAGTTGTAATTTGGAGAGGATGCCTCGGTGGAGG-CCCCGGCCGAAGTGCCTTTGAACGGGCCGTCAGAGAGGGTGA-GAACCCCGTCCGCGGTCGCC-GGCCCCCACCGTGTGAGGCTCCTTCGACGAGTCGAGTTGTTTGGGAATGCAGCTCTAAATGGGAGGTAAATTCCTTCCAAGGCTAAATATCCGCCAGAGACCGATAGCGCACAAGTAGAGTGATCGAAAGGTGAAAAGCACTTTG-AAAAGAGGGTGAAACAGCACGTGAAATTGTTGAAAGGGAAGCGCTTGCAACCAGAGTCGTCCGCGGGGGCTCCACCGACCCTC-GGGCCCGGTGTACTCCCCCGCGGCCGAGCCAGCATCAGCTCGGCCGGTCGGATAAAGGCCGTGGGAATGTGACACCCTC------GGGTGTGTTATAGCCCGCGGCACAATACGGCCTGGCCGGGCTGAGGTCAGCGCCT-TT--GGCACGGATGCTGGCGTAATGGTTGTCAGCGGCCCGTCTTGAAACAC-GG--------------------------------------------ACCAAGGAGTCTAACATCTATGCGAGTGTTCGGGTGTCAAACCCGTGAGCGGAA--TGAAAGTG-AACGGAGGTGGGAG-CCCTC---GGGCGCACCATCG-ACCGATCCGG--ATGTCTTCGGAT-GGATTTGAGTATG-AGCATAG-CTGTTGGGA-CCCGAAAGATGGTGAACTATGCGTGAATAGGGTGAAGCCAGAGG-AAACTCTGGTGGAGGCTCGCAGCGG--------------------------------------------------------------TTCTGAC-GTGCA--------------------------------------------------------------------------------AATCGATCGTCAA--------------ATTTGCGCATA-GGGGCGAAAGACTTATC----------------------------------------------------------------------------------------------------------GAACCAT--------------------------------CTAGTAGCTGGTTCCTGCG-----------------------------------------------------------------------------------------------------------------------------------------------------------------------------------------------------------------------------------------------------------------------------------------------------------------------------------------------------------------------------------------------------------------------------------------------------------------------------------------------------

K.ae.MH872 ---------------------------------------------------------------------------------------------------------------------------------------------------------------------------------------------------------------------------------------------------------------------------------------------------------------------------------------------------------------------------------------------------------------------------------------------------------------------------------------------------------------------------------------------------------------------------------------------------------------------------------------------------------------GCATATC-ATAAGCGGAGGGAAAAGAAACCCAAC-----------------------------------------AGGG-ATTGCCCTAGTAAC--GGCGAGTGAAGCGGCAACAGCCCAAATTTGAAATTCTGACCCCCTGGG---GTCCGAGTTGTAATTTGGAGAGGATGCCTCGGTGGAGG-CCCCGGCCGAAGTGCCTTTGAACGGGCCGTCAGAGAGGGTGA-GAACCCCGTCCGCGGTCGCCGGCCCCCCACCGTGTGAGGCTCCTTCGACGAGTCGAGTTGTTTGGGAATGCAGCTCTAAATGGGAGGTAAATTCCTTCCAAGGCTAAATATCCGCCAGAGACCGATAGCGCACAAGTAGAGTGATCGAAAGGTGAAAAGCACTTTGAAAAAGAGGGTGAAACAGCACGTGAAATTGTTGAAAGGGAAGCGCTTGCAACCAGAGTCGTCCGCGGGGGCTCCACCGACCCTC-GGGCCCGGTGTACTCCCCCGCGGTCGAGCCAGCATCAGCTCGGCCGGTCGGATAAAGGCCGTGGGAATGTGACACCCTC------GGGTGTGTTATAGCCCGCGGCACAATACGGCCTGGCCGGGCTGAGGTCAGCGCCT-TT--GGCACGGATGCTGGCGTAATGGTTGTCAGCGGCCCGTCTTGAAACAC-GG--------------------------------------------ACCAAGGAGTCTAACATCTATGCGAGTGTTCGGGTGTCAAACCCGTGAGCGGAA--TGAAAGTG-AACGGAGGTGGGAG-CCCTC---GGGCGCACCATCG-ACCGATCCGG--ATGTCTTCGGAT-GGATTTGAGTATG-AGCATAG-CTGTTGGGA-CCCGAAAGATGGTGAACTATGCGTGAATAGGGTGAAGCCAGAGG-AAACTCTGGTGGAGGCTCGCAGCGG--------------------------------------------------------------TTCTGAC-GTGCA--------------------------------------------------------------------------------AATCGATCGTCAA--------------ATTTGCGCATA-GGGGCGAAAGACTTATC----------------------------------------------------------------------------------------------------------GAA-CAT--------------------------------CTAGTAGCTGGT--CTGCG-----------------------------------------------------------------------------------------------------------------------------------------------------------------------------------------------------------------------------------------------------------------------------------------------------------------------------------------------------------------------------------------------------------------------------------------------------------------------------------------------------

He.as.MH86 ---------------------------------------------------------------------------------------------------------------------------------------------------------------------------------------------------------------------------------------------------------------------------------------------------------------------------------------------------------------------------------------------------------------------------------------------------------------------------------------------------------------------------------------------------------------------------------------------------------------------------------------------------------------GCATATCAATAAGCGGAGGAAAAGAAA--CCAAC-----------------------------------------AGGGCATTGCCCCAGTAAC--GGCGAGTGAAGCGGCAATAGCTCAAATTTGAAATCCGGCCCTCGG------GTCTGAGTTGTAATTTGAAGAGGATGCCTCGGTGCGGGCCCCCGATCGAAGTTCCTTGGAACAGGACGTCATAGAGGGTGA-GAATCCCGTCTGTGGTCGGT-GGCCCCTACCGTGTGAGGCTCCTTCGACGAGTCGAGTTGTTTGGGAATGCAGCTCTAAATGGGAGGTAAATTTCTTCCAAGGCTAAATATCAGCCAGAGACCGATAGCGCACAAGTAGAGTGATCGAAAGGTGAAAAGCACTTTG-AAAAGAGGGTTAAAAAGCACGTGAAATTGTTGAAAGGGAAGCGCTTGCCACCAGAGTTGTCCG-GGGGGTTCAGCCGACCTTC-TGGCTCGGTGTACTTCCTCTCGGGCAGGCCCGCATCAGTTCGGCCGGCCGTGTAAAGGCCGCGGGAATGTAGCACCCTC------GGGTGTGTTATAGCCCGCGGTGCAATACGGCCTGGCTGGACTGAGGTCCGCG-CT-TC--GGCAAGGATGCGGGCGTAATGGTGGTCAGCGGCCCGTCTTGAAACAC-GG--------------------------------------------ACCAAGGAGTCTAACATCTGTGCGAGTGTTCGGGTGTCAAACCCGTGAGCGTAA--TGAAAGTG-AACGGAGGTGGGAG-CCCTC---GGGCGCACCATCG-ACCGATCCTG--ATGTCCTCGGAT-GGATTTGAGTATG-AGCATAG-CTGTTGGGA-CCCGAAAGATGGTGAACTATGCGTGAATAGGGTGAAGCCAGAGG-AAACTCTGGTGGAGGCTCGCAGCGG--------------------------------------------------------------TTCTGAC-GTGCA--------------------------------------------------------------------------------AATCGATCGTCAA--------------ATTTGCGCATA-GGGGCGAAAGACTAATC----------------------------------------------------------------------------------------------------------GAACCGT--------------------------------CTAGTAGCTGGTTCCTGCCG----------------------------------------------------------------------------------------------------------------------------------------------------------------------------------------------------------------------------------------------------------------------------------------------------------------------------------------------------------------------------------------------------------------------------------------------------------------------------------------------------

El.parv.MN ----------------------------------------------------------------------------------------------------------------------------------------------------------------------------------------------------------------------------------------------------------------------------------------------------------------------------------------------------------------------------------------------------------------------------------------------------------------------------------------------------------------------------------------------------------------------------------------------------------------------------------------------------------------------------------------------------------------------------------------------------------------------------------------------------------------------------------------------------------------------------------------------------------------------------------------------------------------------------------------------------------------------------------------------------------------------TTTG-AAAAGAGAGTTAAAAAGCACGTGAAATTGTTGAAAGGGAAGCGCTTGCAACCAGAGCGGTTTGGAGGGGTTCAGCC-----------TTTGGCGCACTCCCCTCCAGTCAGGCTAGCATCAGTTCGGCCGGTCGGATAAAGGTCGTGGGAATGTGGCACCCTC------GGGTGTGTTATAGCCCATGGCACAATACGGCCTGGCTGGACTGAGGTCCACG-CT-TC--GGCAAGGATGCTGGCGTAATGGTTGTCAGCGGCCCGTCTTGAAACAC-GG--------------------------------------------ACCAAGGAGTCTAACATCTGTGCGAGTGTTCGGGTGTCAAACCCGTGAGCGTAA--TGAAAGTG-AACGGAGGTGGGAG-CCCTC---GGGTGCACCATCG-ACCGATCCTG--ATGTCTTCGGAT-GGATTTGAGTATG-AGCATAG-CTGTTGGGA-CCCGAAAGATGGTGAACTATGCGCGGATAGGGTGAAGCCAGAGG-AAACTCTGGTGGAGGCTCGCAGCGG--------------------------------------------------------------TTCTGAC-GTGCA--------------------------------------------------------------------------------AATCGATCGTCAA--------------ATCTACGCATA-GGGGCGAAAGACTAATC----------------------------------------------------------------------------------------------------------GAACCAT--------------------------------CTAGTAGCTGGTTCCTGCCGAAGTTTCCCTCAGGATA-----------------------------------------------------------------------------------------------------------------------------------------------------------------------------------------------------------------------------------------------------------------------------------------------------------------------------------------------------------------------------------------------------------------------------------------------------------------------------------

MN032446.1 ----------------------------------------------------------------------------------------------------------------------------------------------------------------------------------------------------------------------------------------------------------------------------------------------------------------------------------------------------------------------------------------------------------------------------------------------------------------------------------------------------------------------------------------------------------------------------------------------------------------------------------------------------------------------------------------------------------------------------------------------------------------------------------------------------------------------------------------------------------------------------------------------------------------------------------------------------------------------------------------------------------------------------------------------------------------------TTTG-AAAAGAGAGTTAAAAAGCACGTGAAATTGTTGAAAGGGAAGCGCTTGCAACCAGAGCGGTTTGGAGGGGTTCAGCC-----------TTTGGCGCACTCCCCTCCAGTCAGGCTAGCATCAGTTCGGCCGGTCGGATAAAGGTCGTGGGAATGTGGCACCCTC------GGGTGTGTTATAGCCCATGGCACAATACGGCCTGGCTGGACTGAGGTCCACG-CT-TC--GGCAAGGATGCTGGCGTAATGGTTGTCAGCGGCCCGTCTTGAAACAC-GG--------------------------------------------ACCAAGGAGTCTAACATCTGTGCGAGTGTTCGGGTGTCAAACCCGTGAGCGTAA--TGAAAGTG-AACGGAGGTGGGAG-CCCTC---GGGTGCACCATCG-ACCGATCCTG--ATGTCTTCGGAT-GGATTTGAGTATG-AGCATAG-CTGTTGGGA-CCCGAAAGATGGTGAACTATGCGCGGATAGGGTGAAGCCAGAGG-AAACTCTGGTGGAGGCTCGCAGCGG--------------------------------------------------------------TTCTGAC-GTGCA--------------------------------------------------------------------------------AATCGATCGTCAA--------------ATCTACGCATA-GGGGCGAAAGACTAATC----------------------------------------------------------------------------------------------------------GAACCAT--------------------------------CTAGTAGCTGGTTCCTGCCGAAGT------------------------------------------------------------------------------------------------------------------------------------------------------------------------------------------------------------------------------------------------------------------------------------------------------------------------------------------------------------------------------------------------------------------------------------------------------------------------------------------------

Ca.sal.DQ6 ----------------------------------------------------------------------------------------------------------------------------------------------------------------------------------------------------------------------------------------------------------------------------------------------------------------------------------------------------------------------------------------------------------------------------------------------------------------------------------------------------------------------------------------------------------------------------------------------------------------------------------------------------------------------------------------------------------------------------------------------------------------------------GCAACAGCTCAAATTTGAAATCTGGCGTCTCCGGC---GTCCGAGTTGTAATTTGTAGAGGATGCTTCTGGGCAGC-CACCGGTCTAAGTTCCTTGGAACAGGACGTCATAGAGGGTGA-GAATCCCGTATGCGACCGGA-AGGGCACCCTCCACGTAGCTCCTTCGACGAGTCGAGTTGTTTGGGAATGCAGCTCTAAATGGGAGGTAAATTTCTTCTAAAGCTAAATACCGGCCAGAGACCGATAGCGCACAAGTAGAGTGATCGAAAGATGAAAAGCACTTTG-GAAAGAGAGTTAAAAAGCACGTGAAATTGTTGAAAGGGAAGCGCTTGCAACCAGACTTGCTGGCGG-TGTTCCGCCGGTCTTC--TGACCGGTTCATTCG-CCGTCTGCAGGCCAGCATCATCTGGGGCCGCCGGATAAAAACGGTGGGAATGTGGCTCCCTC------GGGAGTGTTATAGCCCTCCGTGTAATACGGCGTGCCTCGGGTGAGGTCCGCG-CT-TC--GGCTTGGATGCTGGCATAATGGTTGTAAGCGGCCCGTCTTGAAACAC-GG--------------------------------------------ACCAAGGAGTCTAACATCTATGCGAGTGTTCGGGTGTCAAACCCCTACGCGTAA--TGAAAGTG-AACGGAGGTGGGAA-CTTT----TTGTGCACCATCG-ACCGATCCTG--ATGTCTTCNGAT-GGATTTGAGTAAG-ANNATAG-CTGTTGGGA-CCCNAAANATGGTGAACTATGCCTGAATANGGTGAAGCCAGANG-AAACTCTGGTGGAGGNTCGCAGCGGGTNNNNNNNNNNNNNNNNNNNNNNNNNNNNNNNNNNNNNNNNNNNNNNNNNNNNNNNNNNCTTCCTGAG-AGCCAGTGCTACTAAGCGGCATCCCGAAAGGGGGCTGTGGCCGGGGTAATGACCTAGGGTATAGTAACAACGCACTGGATTGGATAATCCGCAGCCAAGTCCTAAACTCAACGTCTTTGTGTA-AAAGCAAAGGGGTAGTATGGATGCAGTTCAACGACTAGAAGGCAGTGGGTCGCATTGTTTCTACATTGAATCAATCGCGGCTTAAGATATAGTCTAGTCCTTACTCGAAAGGGTAGGGTGGAGGAACCGTAATGTCTCTCCCTGAGAGATCATTATTAAATGCTAGTAGCTGGTTCCTGCCGAAGTTTCCCTCAGGATAGCAGTAACGTTTTCAGTTTTATGAGGTAAAGCGAATGATTAGAGGCCTTGGGGTTGAAACAACCTTAACCTATTCTCAAACTTTAAATATGTAAGAAGTCC-TTGTTACTTAGTTGAACGTGGACATTTGAATGTATCGTTACTAGTGGGCCATTTTTGGTAAGCAGAACTGGCGATGCGGGATGAACCGAACGCGGAGTTAAGGTGCCGGAATGCACGCTCATCAGACACCACAAAAGGTGTTAGTTCATCTAGACAGCAGGACGGTGGCCATGGAAGTCGGAATCCGCTAAGGAGTGTGTAACAACTCACCTGCCGAATGAACTAGCCCTGAAAATGGATGGCGCTCAAGCGTGCTACCCATACTCCGCCGCCAGGGTAGAAACGATGCCCT-------------------------------------------------------------------------

Ca.co.DQ24 -----------------------------------------------------------------------------------------------------------------------------------------------------------------------------------------------------------------------------------------------------------------------------------------------------------------------------------------------------------------------------------------------------------------------------------------------------------------------------------------------------------------------------------------------------------------------------------------------------------------------------------------------------------------------------------------------------------------------------------------------ATTGCCCTAGTAAC--GGCGAGTGAAGCGGCAATAGCTCAGATTTGAAATCTGGCGTCTTCGGC---GTCCGAGTTGTAATCTGTAGAGGATGCCTTTGGGTAGC-CACCGGTCTAAGTCCCCTGGAACGGGGCGTCACAGAGGGTGA-GAATCCCGTATGTGACCGGA-AGGGCACCCTCCACAAGGCTCCTTCGACGAGTCGAGTTGTTTGGGAATGCAGCTCTAAATGGGAGGTAAATTCCTTCTAAAGCTAAATACCGGCCAGAGACCGATAGCGCACAAGTAGAGTGATCGAAAGATGAAAAGCACTTTG-GAAAGAGAGTTAAAAAGCACGTGAAATTGTTGAAAGGGAAGCGCTTGCAACCAGACTTGTTGGCGG-TGTTCCGCCGGTCTTC--TGACCGGTCCACTCA-CCGTCTGCAGGCCAGCATCATCTGGGGCCGCCGGATAAAAGCGAGGGGAACGTGGCTCCCTC------GGGAGTGTTATAGCCCCTCGTGCAATACGGCGAGCCTCGGGTGAGGTCCGCG-CT-TC--GGCTAGGATGCTGGCGTAATGGTCGTAAGCGGCCCGTCTTGAAACAC-GG--------------------------------------------ACCAAGGAGTCTAACATCTATGCGAGTGTTCGGGTGTCAAACCCCTACGCGTAA--TGAAAGTG-AACGGAGGTGGGAG-CCTTA---GGGTGCACCATCG-ACCGATCCTG--ATGTCTTCGGAT-GGATTTGAGTAAG-AGCATAG-CTGTTGGGA-CCCGAAAGATGGTGAACTATGCCTGGATAGGGTGAAGCCAGAGG-AAACTCTGGTGGAGGCTCGCAGCGG--------------------------------------------------------------TTCTGAC-GTGCA--------------------------------------------------------------------------------AATCGATCGTCAA--------------ATTTGGGTATA-GGGGCGAAAGACTAATC----------------------------------------------------------------------------------------------------------GAACCAT--------------------------------CTAGTAGCTGGTTCCTGCCNAAGTTTCCCTCAGGATAGCAGTAACGTTTTCAGTTTTATGAGGTAAAGCGAATGATTA------------------------------------------------------------------------------------------------------------------------------------------------------------------------------------------------------------------------------------------------------------------------------------------------------------------------------------------------------------------------------------------------------------------------------------------

Myc.fi.DQ6 ------------------------------------------------------------------------------------------------------------------------------------------------------------------------------------------------------------------------------------------------------------------------------------------------------------------------------------------------------------------------------------------------------------------------------------------------------------------------------------------------------------------------------------------------------------------------------------------------------------------------------------------------------------TAAGCATATCAATAAGCGGAGGAAAAGAAA--CCAAC------------------------------------------AGGGATTGCCCTAGTAAC--GGCGAGTGAAGCGGCAACAGCTCAAATTTGAAATCTGGCGTAA--------GCCCGAGTTGTAATTTGTAGAGGATGCTTCGGGGTAGC-GGCCGGTCTAAGTTCCTTAGAACAGGACGTCATAGAGGGTGA-GAATCCCGTACGTGACTGGCTTGCACCCTCCACGT--AGCTCCTTCGACGAGTCGAGTTGTTTGGGAATGCAGCTCTAAATGGGAGGTAAATTTCTTCTAAAGCTAAATACCGNCCAGAGACCGATAGCGCACAAGTAGAGTGATCGAAAGATGAAAAGCACTTTG-GAAAGAGAGTTAAAAAGCACGTGAAATTGTTGAAAGGGAAGCGCCCGCAACCAGACTTTGCGGCGG-TGTTCGGCCGGTCTTC--TGACCGGTTTACTCG-CCGCCGTGAGGCCATCATCGTCTGGGACCGCTGGATAA-GACCCGAGGAATGTAGCTCCCTT----CGGGGTGTGTTATAGCCTCGGGTG--ATGCAGCGCGTCCCGGGCGAGGTCCGCG-CT-TC--GGCAAGGATGATGGCGTAATGGTTGTCGGCGGCCCGTCTTGAAACAC-GG--------------------------------------------ACCAAGGAGTCTAACATCTATGCGAGTGTTCGGGTGTCAAACCCCTACGCGTAA--TGAAAGTG-AACGGAGGTGGGAA-CTTT----TGGTGCACCATCG-ACCGATCCTG--ATGTCCTCGGAT-GGATTTGAGTAAG-AGCATAG-CTGTTGGGA-CCCGAAAGATGGTGAACTATGCCTGAATAGGGTGAAGCCAGAGG-AAACTCTGGTGGAGGCTCGCAGCGG--------------------------------------------------------------TTCTGAC-GTGCA--------------------------------------------------------------------------------AATCGATCGTCAA--------------ATTTGGGTATA-GGGGCGAAAGACTAATC----------------------------------------------------------------------------------------------------------GAACCAT--------------------------------CTAGTAGCTGGTTCCTGCCGAAGTTTCCCTCAGGATAGCAGTAACGTTTTCAGTTTTATGAGGTAAAGCGAATGATTAGAGGCCTTGGGGTTGAAACAACCTTAACCTATTCTCAAACTTTAAATATGTAAGAAGTCC-TTGTTACTTGGTTGAACGTGGACATTTGAATGTACCGTTACTAGTGGGCCATTTTTGGTAAGCAGAACTGGCGATGCGGGATGAACCGAACGCGAGGTTAAGGTGCCGGAATATACGCTCATCAGACACCACAAAAGGTGTTAGTTCATCTAGACAGCAGGACGGTGGCCATGGAAGTCGGAATCCGCTAAGGAGTGTGTAACAACTCACCTGCCGAATGAACTAGCCCTGAAAATGGATGGCGCTTAAGCGTATTACCCATACCTCGCCGCCAGGGTAGAAACGATGCCCTGGCGAGTA-----------------------------------------------------------------

Myc.pu.DQ4 ------------------------------------------------------------------------------------------------------------------------------------------------------------------------------------------------------------------------------------------------------------------------------------------------------------------------------------------------------------------------------------------------------------------------------------------------------------------------------------------------------------------------------------------------------------------------------------------------------------------------------------------------------------------------------------------------------------------------------------------CAGGGATTGCCCTAGTAAC--GGCGAGTGAAGCGGCAACAGCTCAAATTTGAAATCTGGCGCAA--------GCCCGAGTTGTAATTTGTAGAGGATGCTTCTAGGTAGC-GACCGGTCTAAGTTCCTTGGAACAGGACGTCATAGAGGGTGA-GAATCCCGTATGTGACTGGCCCGCACCTTCTACGT--AGCTCCTTCGACGAGTCGAGTTGTTTGGGAATGCAGCTCTAAATGGGAGGTAAATTTCTTCTAAAGCTAAATACCGGCCAGAGACCGATAGCGCACAAGTAGAGTGATCGAAAGATGAAAAGCACTTTG-GAAAGAGAGTTAAAAAGCACGTGAAATTGTTGAAAGGGAAGCGCTCACAACCAGACTTCTAGGCAG-TGTTCCGCCGGTCTTT--TGACCGGTCTACTCT-CTGTCTCGAGGCCAACATCATCTGGGACCGCCGGATAA-GACCTTAGGAATGTAGCTCCCCT----CGGGGAGTGTTATAGCCTCTGGTG--ATGCGGCGCGTCTCGGGTGAGGTCCGCG-CT-TC--GGCAAGGATGTTGGCGTAATGGTTGTCAGCGGCCCGTCTTGAAACAC-GG--------------------------------------------ACCAAGGAGTCTAACATCTATGCGAGTGTTCGGGTGTCAAACCCCTACGCGTAA--TGAAAGTG-AACGGAGGTGGGAAGCGTA----AGCTGCACCATCG-ACCGATCCTG--ATGTCCTCGGAT-GGATTTGAGTAAG-AGCATAG-CTGTTGGGA-CCCGAAAGATGGTGAACTATGCCTGAATAGGGTGAAGCCAGAGG-AAACTCTGGTGGAGGCTCGCAGCGG--------------------------------------------------------------TTCTGAC-GTGCA--------------------------------------------------------------------------------AATCGATCGTCAA--------------ATTTGGGTATA-GGGGCGAAAGACTAATC----------------------------------------------------------------------------------------------------------GAACCAT--------------------------------CTAGTAGCTGGNTSCTGCCGAAGTTTCCCTCANGATAGCAGTAACGTTTTCAGTTTTATGAGGTAAAGCGAATGATTAGAGGCCTTGGGGTTGAAACAACCTTAACCTATTCTCAAACTTTAAATATGTAAGAAGTCC-TTGTTACTTAATTGAACGTGGACATTTGAATGTATCGTTACTAGTGGGCCATTTTTGGTAAGCAGAACTGGCGATGCGGGATGAACCGAACGCGAGGTTAAGGTGCCGGAATGTACGCTCATCAGACACCACAAAAGGTGTTAGTTCATCTAGACAGCAGGACGGTGGCCATGGAAGTCGGAATCCGCTAAGGAGTGTGTAACAACTCACCTGCCGAATGAACTAGCCCTGAAAATGGATGGCGCTTAAGCGTGCTACCCATACCTCGCCGCCAGGGTAGAAACTATGCCCTGGCGAGTAGGCAGGCGTGGAGGTCCGTG---------------------------------------------

Ce.be.DQ67 ----------------------------------------------------------------------------------------------------------------------------------------------------------------------------------------------------------------------------------------------------------------------------------------------------------------------------------------------------------------------------------------------------------------------------------------------------------------------------------------------------------------------------------------------------------------------------------------------------------------------------------------------------------------------------------------------------------------------------------------------------------------GCGAGTGAAGCGGCAACAGCTCAAATTTGAAATCTGGCGCAA--------GCCCGAGTTGTAATTTGTAGAGGATGCTTCTGGGTAGC-GACCGATCTAAGTTCCTTGGAACAGGACGTCATAGAGGGTGA-GAATCCCGTATGTGACCGGCCCGCACCCTTTACGT--AGCTCCTTCGACGAGTCGAGTTGTTTGGGAATGCAGCTCTAAATGGGAGGTAAATTTCTTCTAAAGCTAAATACCGGCCAGAGACCGATAGCGCACAAGTAGAGTGATCGAAAGATGAAAAGTACTTTG-GAAAGAGAGTTAAAAAGCACGTGAAATTGTTGAAAGGGAAGCGCCTGCAACCAGACTTCGCGGCAG-TGTTCCGCCGGTCTTC--TGACCGGTTCATTCT-CTGTCGCGAGGCCATCATCGTCTGGGCCCGCCGGATAA-GACCTGAGGAATGTGGCTCCCCC--TCGGGGGAGTGTTATAGCCTCTGGTG--ATGCGGCGTGGCTCGGGCGAGGTCCGCG-CT-TC--GGCAAGGATGATGGCGTAATGGTTGTCGGCGGCCCGTCTTGAAACAC-GG--------------------------------------------ACCAAGGAGTCTAACATCTATGCGAGTGTTCGGGTGTCAAACCCCTACGCGTAA--TGAAAGTG-AACGGAGGTGGGAACCGCA----AGGTGCACCATCG-ACCGATCCTG--ATGTCCTCGGAT-GGATTTGAGTAAG-AGCATAG-CTGTTGGGA-CCCGAAAGATGGTGAACTATGCCTGAATAGGGTGAAGCCAGAGG-AAACTCTGGTGGAGGCTCGCAGCGG--------------------------------------------------------------TTCTGAC-GTGCA--------------------------------------------------------------------------------AATCGATCGTCAA--------------ATTTGGGTATA-GGGGCGAAAGACTAATC----------------------------------------------------------------------------------------------------------GAACCAT--------------------------------CTAGTAGCTGGTTCCTGCCGAAGTTTCCCTCAGGATAGCAGTAACGTTTTCAGTTTTATGAGGTAAAGCGAATGATTAGAGGCCTTGGGGTTGAAACAACCTTAACCTATTCTCAAACTTTAAATATGTAAGAAGTCC-TTGTTACTTAGTTGAACGTGGACATTTGAATGTACCGTTACTAGTGGGCCATTTTTGGTAAGCAGAACTGGCGATGCGGGATGAACCGAACGCGAGGTTAAGGTGCCGGAATATACGCTCATCAGACACCACAAAAGGTGTTAGTTCATCTAGACAGCAGGACGGTGGCCATGGAAGTCGGAATCCGCTAAGGAGTGTGTAACAACTCACCTGCCGAATGAACTAGCCCTGAAAATGGATGGCGCTTAAGCGTATTACCCATACCTCGCCGCCAGGGTAGAAACGATGCCCTGGCGAGTAGGCAGGCGTGGAGGCCCGTGACGAA----------------------------------------

T.palu.AY8 -----------------------------------------------------------------------------------------------------------------------------------------------------------------------------------------------------------------------------------------------------------------------------------------------------------------------------------------------------------------------------------------------------------------------------------------------------------------------------------------------------------------------------------------------------------------------------------------------------------------------------------------------------------------------------------------------------------------------------------------------ATTGCCTTAGTAAC--GGCGAGTGAAGCGGCAATAGCTCAAATTTGAAATCTGGCTCTTTCAGA---GTCCGAGTTGTAATTTGTAGAAGATGCTTCGGCTTACG-TATCGGTCTAAGTTCCTTGGAACAGGACGTCACAGAGGGTGA-GAATCCCGTTTGTGGCCGGT--ACTTCCGCCATGTGAAGCTCCTTCGACGAGTCGAGTTGTTTGGGAATGCAGCTCTAAATGGGAGGTAAATTTCTTCTAAAGCTAAATATTGGCCAGAGACCGATAGCGCACAAGTAGAGTGATCGAAAGATGAAAAGCACTTTG-GAAAGAGAGTTAAAAAGTACGTGAAATTGTTGAAAGGGAAGCGCTTGCGACCAGACTTGCCTTTAGTTGCTCCGCCGGTCCTT--CGACCGGTGTATTCTTCTATTGTCAGGCCAGCATCAGTTTGGGTGGTTGGATAAAGGCATTGGGAATGTGACTCTCTT---CGGGGAG-TGTTATAGCCCTTTGTGCAATACAGCCTACCTAGACTGAGGACCGCG-CT-TC--GGCTAGGATGCTGGCGTAATGGTCGTAAGCGGCCCGTCTTGAAACAC-GG--------------------------------------------ACCAAGGAGTCTAACATCTATGCGAGTGTTTGGGTGTCAAACCCGTGCGCGAAA--TGAAAGTG-AACGGAGGTGGGAA-CCTT--A-GGGTGCACCATCG-ACCGATCCTG--ATGTCTTCGGAT-GGATTTGAGTAAG-AGCATAG-CTGTTGGGA-CCCGAAAGATGGTGAACTATGCCTGAATAGGGTGAAGCCAGAGG-AAACTCTGGTGGAGGCTCGCAGCGG--------------------------------------------------------------TTCTGAC-GTGCA--------------------------------------------------------------------------------AATCGATCGTCAA--------------ATTTGGGTATA-GGGGCGAAAGACTAATC----------------------------------------------------------------------------------------------------------GAACCAT--------------------------------TAAACAACTTATT------------------------------------------------------------------------------------------------------AACTTTAGTTA------------------------------------------------------------------------------------------------------------------------------------------------------------------CTAGGTGGTTAAGAGGCGGCTAAAA-----------------------------------------------------------------------------------------------------------------------------------------------------------------------------------------------

T.cer.DQ47 -------------------------------------------------------------------------------------------------------------------------------------------------------------------------------------------------------------------------------------------------------------------------------------------------------------------------------------------------------------------------------------------------------------------------------------------------------------------------------------------------------------------------------------------------------------------------------------------------------------------------------------------------------------------------------------------------------------------------------------------------------------------------------------------------------------------CCCGAATTGTAATTTGTAGAGGATGTTTCNGCTCACG-CCCCGGTCTAAGTTCCTTGGAACAGGACGTCACAGAGGGTGA-GAACCCCGTTTGTGGCCGGC-GGCTTACGCCATGTGAAACTCCTTCGACGAGTCGAGTTGTTTGGGAATGCAGCTCTAAATGGGAGGTAAATTTCTTCTAAAGCTAAATACTGGCCAGAGACCGATAGCGCACAAGTAGAGTGATCGAAAGATGAAAAGCACTTTG-GAAAGAGAGTTAAAAAGTACGTGAAATTGTTGAAAGGGAAGCGCTTGCGACCAGACTTGCCCGCGGATGCTCAGCCGGTCCTT--CGGCCGGTGTACTCTTCCGCGGTCAGGCCAGCATCAGTTCGGGCGGTCGGATAAAGGCCTTGGGAATGTAACTCACCT---CGGTGAG-TGTNATAGCCCTCGGTACAATACGGCCCGCCTGGACTGAGGAACGCG-CT-TC--GGCTAGGATGCTGGCGTAATGGTCGTAAGCGGCCCGTCTTGAAACAC-GG--------------------------------------------ACCAAGGAGTCTAACATCTATGCGAGTGTTTGGGTGTCAAACCCGTGCGCGAAA--TGAAAGTG-AACGGAGGTGGGAG-NCCTCAC-GGGTGCACCATCG-ACCGATCCTG--ATGTCTTCGGAT-GGATTTGAGTAAG-AGCATAG-CTGTTGGGA-CCCGAAAGATGGTGAACTATGCCTGAATAGGGTGAAGCCAGAGG-AAACTCTGGTGGAGGCTCGCAGCGG--------------------------------------------------------------TTCTGAC-GTGCA--------------------------------------------------------------------------------AATCGATCGTCAA--------------ATTTGGGTATA-GGGGCGAAAGACTAATC----------------------------------------------------------------------------------------------------------GAACCAT--------------------------------CTAGTAGCTGGTTCCTGCCGAAGTTTCCCTCAGGATAGCAGTGACGTTTTCAGTTTTATGAGGTAAAGCGAATGATTAGAGGACTGGGGGAAGTAACTTCCTTCACCTATTCTCAAACTTTAAATATGTAAGAAGTCC-TTGTTGCTTAATTGAACGTGGACATTTGAATGTACCGTCACTAGTGGGCCATTTTTGGTAAGCAGAACTGGCGATGCGGGATGAACCGAACGCGAGGTTAAGGTGCCGGAATGCACGCTCATCAGACACCACAAAAGGTGTTAGTTCATCTGGACAGTCGGACGGTGGCCATGGAAGTCGGAATCCGCTAAGGAGTGTGTAACAACTCACCGACCGAATGAACTAGCCCTGAAAATGGATGGCGCTCAAGCGTGCTACCCATACCTCGCCGCCAGGGTAGATACGATGCCCTGGCGAGTAGGCAGGCGTGGAGGTCAGTG---------------------------------------------

Ac.sc.GQ85 --------------------------------------------------------------------------------------------------------------------------------------------------------------------------------------------------------------------------------------------------------------------------------------------------------------------------------------------------------------------------------------------------------------------------------------------------------------------------------------------------------------------------------------------------------------------------------------------------------------------------------------------------------------------------------------------------------------------------------------------------GCCTCAGTAAC--GGCGAGTGAAGCGGCAATAGCTCAAATTTGAAATCTGACTCTTTCAGG---GTCCGAGTTGTAATTTGTAGAGGATGTTTCGGCTTACG-CCCCGGTCTAAGTTCCTTGGAACAGGACGTCACAGAGGGTGA-GAATCCCGTTTGTGGCCGGC-GGCTTCCGCCATGTGAAACTCCTTCGACGAGTCGAGTTGTTTGGGAATGCAGCTCTAAATGGGAGGTAAATTTCTTCTAAAGCTAAATACTGGCCAGAGACCGATAGCGCACAAGTAGAGTGATCGAAAGATGAAAAGCACTTTG-GAAAGAGAGTTAAAAAGTACGTGAAATTGTTGAAAGGGAAGCGCTTGCGACCAGACTTGCCCGCGGATGCTCAACCGGTCCTT--CGGCCGGTGCACTCTTCCGCGGTCAGGCCAGCATCAGTTCGGACGGTTGGATAAAGGCCTTGGGAATGTGGCTCTCTT---CGGGGAG-TGTTATAGCCCTTGGTGCAATACAGCCTGCCTGGACTGAGGCCCGCG-CT-TC--GGCTAGGATGCTGGCGTAATGGTCGTAAGCGGCCCGTCTTGAAA-----------------------------------------------------------------------------------------------------------------------------------------------------------------------------------------------------------------------------------------------------------------------------------------------------------------------------------------------------------------------------------------------------------------------------------------------------------------------------------------------------------------------------------------------------------------------------------------------------------------------------------------------------------------------------------------------------------------------------------------------------------------------------------------------------------------------------------------------------------------------------------------------------------------------------------------------------------------------------------------------------------------------------------------------------------------------------------------------------------------------------------------------------------------------------------

Do.ca.DQ47 ------------------------------------------------------------------------------------------------------------------------------------------------------------------------------------------------------------------------------------------------------------------------------------------------------------------------------------------------------------------------------------------------------------------------------------------------------------------------------------------------------------------------------------------------------------------------------------------------------------------------------------------------------------------------------------------------------------------------------------------AGGG-ATTGCCCTAGTAAC--GGCGAGTGAAGCGGCAATAGCTCAAATTTGAAAGCTGGCCTTCT---G---GTCCGCATTGTAATTTGTAGAGGATGCTTTTAGGCAGC-CGCCGGTCTAAGTTCCTTGGAACAGGACGTCATAGAGGGTGA-GAATCCCGTATGTGACCGGCTCTGGCACCTTATGTAAAGCTCCTTCGACGAGTCGAGTTGTTTGGGAATGCAGCTCTAAATGGGAGGTAAATTTCTTCTAAAGCTAAATACTGGCGAGAGACCGATAGCGCACAAGTAGAGTGATCGAAAGATGAAAAGCACTTTG-GAAAGAGAGTTAAAAAGCACGTGAAATTGTTGAAAGGGAAGCGCTTGCAATCAGACTTGGACTTGGCTGTTCAACCGGTCTTC--TGACCGGCCTACTCAGTCTTGTCCAGGCCAGCATCAGTTTCGGCGGCCGGATAAAGGCCCTGGGAATGTAGCTGTCTCTTCGGGGACAGTGTTATAGCCCAGGGTGTAATACGGCCAGCTGGGACTGAGGTCCGCG-CT-TC--GGCTAGGATGCTGGCGTAATGGTTGTAAGCGGCCCGTCTTGAAACAC-GG--------------------------------------------ACCAAGGAGTCTAACATCTATGCGAGTGTTAGGGTGTCAAACCCTTACGCGTAA--TGAAAGTG-AACGGAGGTGGGAA-CCCGCAA-GGGTGCACCATCG-ACCGATCCTG--ATGTCTTCGGAT-GGATTTGAGTAAG-AGCATAG-CTGTTGGGA-CCCGAAAGATGGTGAACTATGCCTGAATAGGGTGAAGCCAGAGG-AAACTCTGGTGGAGGCTCGCAGCGG--------------------------------------------------------------TTCTGAC-GTGCA--------------------------------------------------------------------------------AATCGATCGTCAA--------------ATTTGGGTATA-GGGGCGAAAGACTAATC----------------------------------------------------------------------------------------------------------GAACCAT--------------------------------CTAGTAGCTGGTTCCTGCCGAAGTTTCCCTCAGGATAGCAGTAACGTTTTCAGTTTTATGAGGTAAAGCGAATGATTAGAGGCCTTGGGGATGAAACATCCTTAACCTATTCTCAAACTTTAAATATGTAAGAAGTCC-TTGTTACTTAGTTGAACGTGGACATTTGAATGCACCGTTACTAGTGGGCCATTTTTGGTAAGCAGAACTGGCGATGCGGGATGAACCGAACGCGAGGTTAAGGTGCCGGAATACACGCTCATCAGACACCACAAAAGGTGTTAGTTCATCTAGACAGCAGGACGGTGGCCATGGAAGTCGGAATCCGCTAAGGAGTGTGTAACAACTCACCTGCCGAATGAACTAGCCCTGAAAATGGATGGCGCTCAAGCGTGTTACCCATACCTCGCCGCCAT------------------------------------------------------------------------------------------

Dd.in.DQ24 ------------------------------------------------------------------------------------------------------------------------------------------------------------------------------------------------------------------------------------------------------------------------------------------------------------------------------------------------------------------------------------------------------------------------------------------------------------------------------------------------------------------------------------------------------------------------------------------------------------------------------------------------------------------------------------------------------------------------------------------CAGGGATTGCCCTAGTAAC--GGCGAGTGAAGCGGCAATAGCTCAAATTTGAAAGCTGGCTTATG-------GCCCGCATTGTAATTTGTAGAGGATGCTTTTAGGCAGC-CGCCGGTCTAAGTTCCTTGGAACAGGACGTCATAGAGGGTGA-GAATCCCGTATGTGACCGGCTCTGGCACCTTATGTAAAGCTCCTTCGACGAGTCGAGTTGTTTGGGAATGCAGCTCTAAATGGGAGGTAAATTTCTTCTAAAGCTAAATACTGGCGAGAGACCGATAGCGCACAAGTAGAGTGATCGAAAGATGAAAAGCACTTTG-GAAAGAGAGTTAAAAAGCACGTGAAATTGTTGAAAGGGAAGCGCTTGCAATCAGACTTGGACTTGGCTGTTCAACAGGTCTTC--TGACCTGCCTATTCAGTCTTGTCCAGGCCAGCATCAGTTTCGGCGGCCGGATAAAGGCCCTGGGAATGTGGCTTCTCCTTCGGGGGAAGTGTTATAGCCCAGGGTGTAATACGGCCAGCTGGGACTGAGGTCCGCG-CT-TC--GGCTAGGATGCTGGCGTAATGGTTGTAAGCGGCCCGTCTTGAAACAC-GG--------------------------------------------ACCAAGGAGTCTAACATCTATGCGAGTGTTAGGGTGTCAAACCCTTACGCGTAA--TGAAAGTG-AACGGAGGTGAGAA-CCCGCAA-GGGTGCATCATCG-ACCGATCCTG--ATGTCTTCGGAT-GGATTTGAGTAAG-AGCATAG-CTGTTGGGA-CCCGAAAGATGGTGAACTATGCCTGAATAGGGTGAAGCCAGAGG-AAACTCTGGTGGAGGCTCGCAGCGG--------------------------------------------------------------TTCTGAC-GTGCA--------------------------------------------------------------------------------AATCGATCGTCAA--------------ATTTGGGTATA-GGGGCGAAAGACTAATC----------------------------------------------------------------------------------------------------------GAACCAT--------------------------------CTAGTAGCTGGTTCCTNCCGAAGTTTCCCTCAGGATNGCAGTAACGTTTTCAGTTTTATGAGGTAAAGCGAATGATTAGAGGCCTTGGGGATGAAACATCCTTAACCTATTCTCAAACTTTAAATATGTAAGAAGTCC-TTGTTTCTTAGTTGAACGTGGACATTTGAATGCACCGTTACTAGTGGGCCATTTTTGGTAAGCAGAACTGGCGATGCGGGATGAACCGAACGCGAGGTTAAGGTGCCGGAATACACGCTCATCAGACACCACAAAAGGTGTTAGTNCATCTAGACAGCNGGACGGTGGCCATGGAAGTCGGAATCCGCTAAGGANTGTGTAACAACTCACCTGCCGAATGAACTAGCCCTGAAAATGGATGGCGCTCAAGCGTGTTACCCATACCTCGCCGCCATGGTAGATTCGAAGCCATGGCGAGTAGGCAGGCGTGGAGGTCA------------------------------------------------

Dd.sam.AY5 ------------------------------------------------------------------------------------------------------------------------------------------------------------------------------------------------------------------------------------------------------------------------------------------------------------------------------------------------------------------------------------------------------------------------------------------------------------------------------------------------------------------------------------------------------------------------------------------------------------------------------------------------------------TAAGCATATCAATAAGCGGAGGAAAAGAAA--CCAAC------------------------------------------AGGGATTGCCCTATTAAC--GGCGAGTGAAGCGGCAATAGCTCAAATTTGAAAGCTGGCTTATG-------GCCCGCATTGTAATTTGTAGAGGATGCTTTTAGGCAGC-CGCCGGTCTAAGTTCCTTGGAACAGGACGTCATAGAGGGTGA-GAATCCCGTATGTGACCGGCTCTGGCACCTTATGTAAAGCTCCTTCGACGAGTCGAGTTGTTTGGGAATGCAGCTCTAAATGGGAGGTAAATTTCTTCTAAAGCTAAATACTGGCGAGAGACCGATAGCGCACAAGTAGAGTGATCGAAAGATGAAAAGCACTTTG-GAAAGAGAGTTAAAAAGCACGTGAAATTGTTGAAAGGGAAGCGCTTGCAATCAGACTTGGACTTGGCTGTTCAACAGGTCTTC--TGACCTGCCTATTCAGTCTTGTCCAGGCCAGCATCAGTTTCGGCGGCCGGATAAAGGCTCTGGGAATGTGGCTTTCCCTTCGGGGGAAGTGTTATAGCCCAGGGTGTAATACGGCCAGCTGGGACTGAGGTCCGCG-CT-TC--GGCTAGGATGCTGGCGTAATGGTTGTAAGCGGCCCGTCTTGAAACAC-GG--------------------------------------------ACCAAGGAGTCTAACATCTATGCGAGTGTTAGGGTGTCAAACCCTTACGCGTAA--TGAAAGTG-AACGGAGGTGAGAA-CCCGCAA-GGGTGCATCATCG-ACCGATCCTG--ATGTCTTCGGAT-GGATTTGAGTAAG-AGCATAG-CTGTTGGGA-CCCGAAAGATGGTGAACTATGCCTGAATAGGGTGAAGCCAGAGG-AAACTCTGGTGGAGGCTCGCAGCGG--------------------------------------------------------------TTCTGAC-GTGCA--------------------------------------------------------------------------------AATCGATCGTCAA--------------ATTTGGGTATA-GGGGCGAAAGACTAATC----------------------------------------------------------------------------------------------------------GAACCAT--------------------------------CTAGTAGCTGGTTCCTGCCGAAGTTTCCCTCAGGATAGCAGTAACGTTTTCAGTTTTATGAGGTAAAGCGAATGATTAGAGGCCTTGGGGATGAAACATCCTTAACCTATTCTCAAACTTTAAATATGTAAGAAGTCC-TTGTTACTTAATTGAACGTGGACATTTGAATGCACCGTTACTAGTGGGCCATTTTTGGTAAGCAGAACTGGCGATGCGGGATGAACCGAACGCGAGGTTAAGGTGCCGGAATACACGCTCATCAGACACCACAAAAGGTGTTAGTTCATCTAGACAGCAGGACGGTGGCCATGGAAGTCGGAATCCGCTAAGGAGTGTGTAACAACTCACCTGCCGAATGAACTAGCCCTGAAAATGGATGGCGCTCAAGCGTGTTACCCATACCTCGCCGCCATGGTAGATTCGAAGCCATGGCGAGTAGGCAGGCGTGGAGGTCAGTGACGAAGCCTTCGGGGTGACCGGGGGTAGAACGACCTCTAGTGCAG

Dd.hi.DQ67 -----------------------------------------------------------------------------------------------------------------------------------------------------------------------------------------------------------------------------------------------------------------------------------------------------------------------------------------------------------------------------------------------------------------------------------------------------------------------------------------------------------------------------------------------------------------------------------------------------------------------------------------------------------------------------------------------------------------------------------------------------------------------------------------------------------------------------------------------------------------------------------------------------------------------------------------------------------------------------------------------------------------------------------------------------------------------------------GTTAAAAAGCACGTGAAATTGTTGAAAGGGAAGCGCTTGCAATCAGACTTGGACTTGACTGTTCAACAGGTC-TC--TGACCTGCCTATTCAGTC-TGTCCAGGCCAGCATCAGTTTCGGCGGCCGGATAAAGGCCCTAGGAATGTGGCTTTCCCTTCGGGGGAAGTGTTATAGCCTAGGGTGTAATACGGCCAGCTGGGACTGAGGTCCGCG-CT-TC--GGCTAGGATGCTGGCGTAATGGTTGTAAGCGGCCCGTCTTGAAACAC-GG--------------------------------------------ACCAAGGAGTCTAACATCTATGCGAGTGTTAGGGTGTCAAACCCTTACGCGTAA--TGAAAGTG-AACGGAGGTGAGAA-CCCGCAA-GGGTGCATCATCG-ACCGATCCTG--ATGTCTTCGGAT-GGATTTGAGTAAG-AGCATAG-CTGTTGGGA-CCCGAAAGATGGTGAACTATGCCTGAATAGGGTGAAGCCAGAGG-AAACTCTGGTGGAGGCTCGCAGCGG--------------------------------------------------------------TTCTGAC-GTGCA--------------------------------------------------------------------------------AATCGATCGTCAA--------------ATTTGGGTATA-GGGGCGAAAGACTAATC----------------------------------------------------------------------------------------------------------GAACCAT--------------------------------CTAGTAGCTGGTTCCTGCCGAAGTTTCCCTCAGGATAGCAGTAACGTTTTCAGTTTTATGAGGTAAAGCGAATGATTAGAGGCCTTGGGGATGAAACATCCTTAACCTATTCTCAAACTTTAAATATGTAAGAAGTCC-TTGTTACTTAGTTGAACGTGGACATTTGAATGCACCGTTACTAGTGGGCCATTTTTGGTAAGCAGAACTGGCGATGCGGGATGAACCGAACGCGAGGTTAAGGTGCCGGAATACACGCTCATCAGACACCACAAAAGGTGTTAGTTCATCTAGACAGCAGGACGGTGGCCATGGAAGTCGGAATCCGCTAAGGAGTGTGTAACAACTCACCTGCCGAATGAACTAGCCCTGAAAATGGATGGCGCTCAAGCGTGTTACCCATACCTCGCCGCCATGGTAGATTCGAAGCCATGGCGAGTAGGCAGGCGTGGAGGTCAGT----------------------------------------------

Myr.du.DQ6 ----------------------------------------------------------------------------------------------------------------------------------------------------------------------------------------------------------------------------------------------------------------------------------------------------------------------------------------------------------------------------------------------------------------------------------------------------------------------------------------------------------------------------------------------------------------------------------------------------------------------------------------------------------------------------------------------------------------------------------------------------------------------------GCAACAGCTCAAATTTGAAATCTGGCCTCTTTGGG---GTCCGAATTGTAATTTGGAGAGGATGTTTTTGGGTGTC-CGCCGGCCTAAGTCCCTTGGAACAGGGCGTCATAGAGGGTGA-GAATCCCGTATGTGGCCGGA-AAGGTACCCTCCGTAAAACTCCTTCGACGAGTCGGGTTGTTTGGGAATGCAGCCCTAAATGGGAGGTAAATTTCTTCTAAAGCTAAATACCGGCCAGAGACCGATAGCGCACAAGTAGAGTGATCGAAAGATGAAAAGCACTTTG-GAAAGAGAGTTAAACAGCACGTGAAATTGTTGAAAGGGAAGCGCTTGCGATCAGTCTCGACGGCGGCCGTTCGGCCTCTCTTC--TGAGTGGTTTATTCGGTCGCCGCCGGGCCAGCATCAGTTTCGGCGGTTGGATAAAGGTTGTGGGAATGTGGCCCCCTC------GGGGGTGTTATAGCCCACTTCGTAATACAACCAGCTGGGACTGAGGTCCGCG-CT-TC--GGCTAGGATGCTGGCAAAATGGTCGTAAGCGGCCCGTCTTGAAACAC-GG--------------------------------------------ACCAAGGAGTCTAACATCTACGCGAGTGTTCGGGTGTCAAACCCGTGCGCGCAA--TGAAAGTG-AACGGAGGTGGGAT-CCCGCAA-GGGCGCACCATCG-ACCGATCCTG--ATGTCTTCGGAT-GGATTTGAGTAAG-AGCGTAG-CTGTTGGGA-CCCGAAAGATGGTGAACTATGCCTGAATAGGGTGAAGCCAGAGG-AAACTCTGGTGGAGGCTCGCAGCGG--------------------------------------------------------------TTCTGAC-GTGCA--------------------------------------------------------------------------------AATCGATCGTCAA--------------ATTTGGGTATA-GGGGCGAAAGACTAATC----------------------------------------------------------------------------------------------------------GAACCAT--------------------------------CTAGTAGCTGGTTCCTGCCGAAGTTTCCCTCAGGATAGCAGTAACGTTTTCAGTTTTATGAGGTAAAGCGAATGATTAGAGGCCTTGGGGTTGAAACAACCTTAACCTATTCTCAAACTTTAAATATGTAAGAAGCCC-TTGTTACTTAGTTGAACGTGGGCGTTAGAATGTATCGTTACTAGTGGGCCATTTTTGGTAAGCAGAACTGGCGATGCGGGATGAACCGAACGCGAGGTTAAGGTGCCGGAATGCACGCTCATCAGACACCACAAAAGGTGTTAGTTCATCTAGACAGCAGGACGGTGGCCATGGAAGTCGGAATCCGCTAAGGAGTGTGTAACAACTCACCTGCCGAATGAACTAGCCCTGAAAATGGATGGCGCTCAAGCGTGTTACCCATACCTCGCCGCCAGGGCAGATGCTATGCCCTGGCGAGTAGGCAGGCGTGGAGGCCCGT----------------------------------------------

Es.phs.DQ6 ------------------------------------------------------------------------------------------------------------------------------------------------------------------------------------------------------------------------------------------------------------------------------------------------------------------------------------------------------------------------------------------------------------------------------------------------------------------------------------------------------------------------------------------------------------------------------------------------------------------------------------------------------------------------------------------------------------------------------------------------------------------------------------------------TCTGGCGCCCCCGGC---GTCCGAGTTGTAATTTGTAGAGGATGCTATTGGGTTGC-CACCGGTCTAAGTTCCTTGGAACAGGACGTCACAGAGGGTGA-GAATCCCGTATGTGACCGGC-CAGGCGCCTTCTGTATAGCTCCTTCGACGAGTCGGGTTGTTTGGGAATGCAGCCCTAAATGGGAGGTAAATTTCTTCTAAAGCTAAATACCGGCCAGAGACCGATAGCGCACAAGTAGAGTGATCGAAAGATGAAAAGCACTTTG-GAAAGAGAGTTAAAAAGCACGTGAAATTGTTGAAAGGGAAGCGCTTGCAATCAGTCTCGACGGCGGCTGTTCGGCCTCTCTTC--TGAGTGGTTTATTCAGTCGCCGCCGGGCCAGCATCAGTTTTGGCGGTCGGATAAAGGCGCAGGGAATGTAGCTCCCCC------GGGAGTGTTATAGCCCCGCGTGCAATACGGCCCGCCGGGACTGAGGTCCGCG-CT-TC--GGCTAGGATGCTGGCGAAATGGTTGTAAGCGGCCCGTCTTGAAACAC-GG--------------------------------------------ACCAAGGAGTCTAACATCTACGCGAGTGTTTGGGTGTCAAACCCGTGCGCGCAA--TGAAAGTG-AACGGAGGTGGGAACCGCA----AGGTGCACCATCG-ACCGATCCTG--ATGTCTTCGGAT-GGATTTGAGTAAG-AGCGTAG-CTGTTGGGA-CCCGAAAGATGGTGAACTATGCCTGAATAGGGTGAAGCCAGAGG-AAACTCTGGTGGAGGCTCGCAGCGG--------------------------------------------------------------TTCTGAC-GTGCA--------------------------------------------------------------------------------AATCGATCGTCAA--------------ATTTGGGTATA-GGGGCGAAAGACTAATC----------------------------------------------------------------------------------------------------------GAACCAT--------------------------------CTAGTAGCTGGTTCCTGCCGAAGTTTCCCTCAGGATAGCAGTAACGTTTTCAGTTTTATGAGGTAAAGCGAATGATTAGAGGCCTTGGGGTTGAAACAACCTTAACCTATTCTCAAACTTTAAATATGTAAGAAGCCC-TTGTTACTTAGTTGAACGTGGGCATTTGAATGTATCGTTACTAGTGGGCCATTTTTGGTAAGCAGAACTGGCGATGCGGGATGAACCGAACGCGAGGTTAAGGTGCCGGAATGCACGCTCATCAGACACCACAAAAGGTGTTAGTTCATCTAGACAGCAGGACGGTGGCCATGGAAGTCGGAATCCGCTAAGGAGTGTGTAACAACTCACCTGCCGAATGAACTAGCCCTGAAAATGGATGGCGCTCAAGCGTGCTACCCATACCTCGCCGCCGGGGCAGATGCAACGCCCCGGCGAGTAGGCAGGC----------------------------------------------------------

Es.cen.DQ6 -------------------------------------------------------------------------------------------------------------------------------------------------------------------------------------------------------------------------------------------------------------------------------------------------------------------------------------------------------------------------------------------------------------------------------------------------------------------------------------------------------------------------------------------------------------------------------------------------------------------------------------------------------------------------------------------------------------------------------------------------------------------------------ACAGCTCANATTTGANNTCTGGCGCCTCCGGC---GTCCGAGTTGTAATTTGTAGAGGATGCTATTGGGTTGC-CACCGGTCTAAGTTCCTTGGAACAGGACGTCACAGAGGGTGA-GAATCCCGTACGTGACCGGC-CAGGCGCTCTCTGTATAGCTCCTTCGACGAGTCGGGTTGTTTGGGAATGCAGCCCTAAATGGGAGGTAAATTTCTTCTAAAGCTAAATACCGGCCAGAGACCGATAGCGCACAAGTAGAGTGATCGAAAGATGAAAAGCACTTTG-GAAAGAGAGTTAAAAAGCACGTGAAATTGTTGAAAGGGAAGCGCTTGCAATCAGTCTCGACGGCGGCTGTTCGGCCTCTCTTC--TGAGTGGTTTATTCAGTCGCCGCCGGGCCAGCATCAGTTTTGGCGGCCGGATAAAGGCGCGGGGAATGTAGCTCCCTC------GGGAGTGTTATAGCCCCGTGTGCAATACGGCCCGCCGGGACTGAGGTCCGCG-CT-TC--GGCTAGGATGCTGGCGAAATGGTTGTAAGCGGCCCGTCTTGAAACAC-GG--------------------------------------------ACCAAGGAGTCTAACATCTACGCGAGTGTTTGGGTGTCAAACCCGTGCGCGCAA--TGAAAGTG-AACGGAGGTGGGAACCGCA----AGGTGCACCATCG-ACCGATCCTG--ATGTCTTCGGAT-GGATTTGAGTAAG-AGCGTAG-CTGTTGGGA-CCCGAAAGATGGTGAACTATGCCTGAATAGGGTGAAGCCAGAGG-AAACTCTGGTGGAGGCTCGCAGCGG--------------------------------------------------------------TTCTGAC-GTGCA--------------------------------------------------------------------------------AATCGATCGTCAA--------------ATTTGGGTATA-GGGGCGAAAGACTAATC----------------------------------------------------------------------------------------------------------GAACCAT--------------------------------CTAGTAGCTGGTTCCTGCCGAAGTTTCCCTCAGGATAGCAGTAACGTTTTCAGTTTTATGAGGTAAAGCGAATGATTAGAGGCCTTGGGGTTGAAACAACCTTAACCTATTCTCAAACTTTAAATATGTAAGAAGCCC-TTGTTACTTAGTTGAACGTGGGCATTTGAATGTATCGTTACTAGTGGGCCATTTTTGGTAAGCAGAACTGGCGATGCGGGATGAACCGAACGCGAGGTTAAGGTGCCGGAATGCACGCTCATCAGACACCACAAAAGGTGTTAGTTCATCTAGACAGCAGGACGGTGGCCATGGAAGTCGGAATCCGCTAAGGAGTGTGTAACAACTCACCTGCCGAATGAACTAGCCCTGAAAATGGATGGCGCTCAAGCGTGCTACCCATACCTCGCCGCCGGGGCAGANGCAACGCCCCGGCGAGTAGGCAGGCGTGGAGGCCC------------------------------------------------

G.bi.DQ678 -----------------------------------------------------------------------------------------------------------------------------------------------------------------------------------------------------------------------------------------------------------------------------------------------------------------------------------------------------------------------------------------------------------------------------------------------------------------------------------------------------------------------------------------------------------------------------------------------------------------------------------------------------------TTAAGCATATCAATAAGCGGAGGAAAAGAAA--CCAAC-----------------------------------------AGGG-ATTGCCTCAGTAAC--GGCGAGTGAAGCGGCAATAGCTCAAATTTGAAAGCTGGCGTCTTCGAC---GTCCGCGTTGTAATTTGTAGAGGATGCTTCGGCGAGGA-CTCCTGCCTAAGTCCCCTGGAACGGGGCGTCACAGAGGGTGA-GAATCCCGTACGTGGCGGGC-GGTCCGAGCCATGTGAAGCTCCTTCGACGAGTCGAGTTGTTTGGGAATGCAGCTCTAAATGGGAGGTAAATTTCTTCTAAAGCTAAATACTGGCCAGAGACCGATAGCGCACAAGTAGAGTGATCGAAAGATGAAAAGCACTTTG-GAAAGAGAGTTAAAAAGTACGTGAAATTGTTGAAAGGGAAGCGCTTGCAACCAGACTCGCTCGCAGTTGCTCAGCCGGCCTCT--TGGCCGGTGCACTCTTCTGCGATCGGGCCAGCATCAGTTCGGGCGGCCGGATAAAGGCGTCGGGAATGTAGCACCCTT---CGGGGTG-TGTTATAGCCCGGCGCGGAATGCGGCCAGCCTGGACTGAGGATCTCG-CT-TC--GGCAAGGATGCTGGCGTAATGGTTGCAAGCGGCCCGTCTTGAAACAC-GG--------------------------------------------ACCAAGGAGTCTAACATCTATGCGAGTGTTTGGGTGTCAAACCCATGCGCGTAA--TGAAAGTG-AACGGAGGTGGGAACCCCTCGCGGGGCGCACCATCG-ACCGATCCTG--ATGTCTTCGGAT-GGATTTGAGTAAG-AGCATAG-CTGTTGGGA-CCCGAAAGATGGTGAACTATGCCTGAATAGGGTGAAGCCAGAGG-AAACTCTGGTGGAGGCTCGCAGCGG--------------------------------------------------------------TTCTGAC-GTGCA--------------------------------------------------------------------------------AATCGATCGTCAA--------------ATTTGGGTATA-GGGGCGAAAGACTAATC----------------------------------------------------------------------------------------------------------GAACCAT--------------------------------CTAGTAGCTGGTTCCTGCCGAAGTTTCCCTCAGGATAGCAGTAACGAATTCAGTTTTATGAGGTAAAGCGAATGATTAGAGGCCTTGGGGTTGAAACAACCTTAACCTATTCTCAAACTTTAAATATGTAAGAAGTCC-TTGTTACTTAGTTGAACGTGGACATTTGAATGTACCGTTACTAGTGGGCCATTTTTGGTAAGCAGAACTGGCGATGCGGGATGAACCGAACGCGATGTTAAGGTGCCGGAATGCACGCTCATCAGACACCACAAAAGGTGTTAGTTCATCTAGACAGCAGGACGGTGGCCATGGAAGTCGGAATCCGCTAAGGAGTGTGTAACAACTCACCTGCCGAATGAACTAGCCCTGAAAATGGATGGCGCTCAAGCGTGCTACCCATACATCGCCGCCAGGGTAGAT-----------------------------------------------------------------------------------

B.v.DQ6780 -------------------------------------------------------------------------------------------------------------------------------------------------------------------------------------------------------------------------------------------------------------------------------------------------------------------------------------------------------------------------------------------------------------------------------------------------------------------------------------------------------------------------------------------------------------------------------------------------------------------------------------------------------------------------------------------------------------------------------------------------------------------AGTGAAGCGGCAATAGCTCAAATTTGAAAGCTGGCCCCTTTGGG---GTCCGCGTTGTAATTTGTAGAGGATGATTCGGCGAGGG-CTCCCGTCTAAGTCCCCTGGAACGGGGCGTCATAGAGGGTGA-GAATCCCGTATGTGATGGGT-TGCCTTAGCCATGTGAATCTCCTTCGACGAGTCGAGTTGTTTGGGAATGCAGCTCTAAATGGGAGGTAAATTTCTTCTAAAGCTAAATACCGGCCAGAGACCGATAGCGCACAAGTAGAGTGATCGAAAGATGAAAAGCACTTTG-GAAAGAGAGTTAAAAAGTACGTGAAATTGTTGAAAGGGAAGCGCTTGCAGCCAGACTTGTCCGCAGTTGCTCAGCCGGTCTCC--TGACCGGCGTACTCTTCTGCGGCCAGGCCAGCATCAGTTCGGGCGGTCGGATAAAGACCCTAGGAATGTAGCTCCTCT---CGGGGAG-TGTTATAGCCTGGGGTGGAATGCGGCCAGCCTGGACTGAGGATCTCG-CT-TC--GGCTAGGATGCTGGCGTAATGGCTGTAAGCGGCCCGTCTTGAAACAC-GG--------------------------------------------ACCAAGGAGTCTAACATCTATGCGAGTGTTTGGGTGTCAAACCCATACGCGTAA--TGAAAGTG-AACGGAGGTGGGAA-CCCTCAC-GGGTGCACCATCG-ACCGATCCTG--ATGTCTTCGGAT-GGATTTGAGTAAG-AGCATAG-CTGTTGGGA-CCCGAAAGATGGTGAACTATGCCTGAATAGGGTGAAGCCAGAGG-AAACTCTGGTGGAGGCTCGCAGCGG--------------------------------------------------------------TTCTGAC-GTGCA--------------------------------------------------------------------------------AATCGATCGTCAA--------------ATTTGGGTATA-GGGGCGAAAGACTAATC----------------------------------------------------------------------------------------------------------GAACCAT--------------------------------CTAGTAGCTGGTTCCTGCCGAAGTTTCCCTCAGGATAGCAGTAACGAATTCAGTTTTATGAGGTAAAGCGAATGATTAGAGGCCTTGGGGTTGAAACAACCTTAACCTATTCTCAAACTTTAAATATGTAAGAAGTCC-TTGTTACTTAGTTGAACGTGGACACTTGAATGTACCGTTACTAGTGGGCCATTTTTGGTAAGCAGAACTGGCGATGCGGGATGAACCGAACGCGATGTTAAGGTGCCGGAATGCACGCTCATCAGACACCACAAAAGGTGTTAGTTCATCTAGACAGCAGGACGGTGGCCATGGAAGTCGGAATCCGCTAAGGAGTGTGTAACAACTCACCTGCCGAATGAACTAGCCCTGAAAATGGATGGCGCTCAAGCGTGCTACCCATACATCGCCGCCAGGGTAGATACGATGCCCTGGCGAGTAGGCAGGCGTGGAGGCCCGT----------------------------------------------

Mac.phsn.D ----------------------------------------------------------------------------------------------------------------------------------------------------------------------------------------------------------------------------------------------------------------------------------------------------------------------------------------------------------------------------------------------------------------------------------------------------------------------------------------------------------------------------------------------------------------------------------------------------------------------------------------------------------------------------------------------------------------------------------------------------------------------------GCAACAGCTCAAATTTGAAAGCTGGCTCCTTCGGA---GTCCGCGTTGTAATTTGTAGAGGATGATTCGGCGAGGG-CTCCCGCCTAAGTCCCCTGGAACGGGGCGTCATAGAGGGTGA-GAATCCCGTATGCGGTGGGT-TGCCTTAGCCATGTGAATCTCCTTCGACGAGTCGAGTTGTTTGGGAATGCAGCTCTAAATGGGAGGTAAATTTCTTCTAAAGCTAAATACCGGCCAGAGACCGATAGCGCACAAGTAGAGTGATCGAAAGATGAAAAGCACTTTG-GAAAGAGAGTTAAAAAGTACGTGAAATTGTTGAAAGGGAAGCGCTTGCAGCCAGACTTGTCCGCAGTTGCTCAACCGGTCTCC--TGACCGGTGTACTCTTCTGCGGCCAGGCCAGCATCAGTTCGGGCGGTCGGATAAAGGCCCTGGGAATGTAGCTCCTCT---CGGGGAG-TGTTATAGCCCAGGGTGGAATGCGGCCAGCCTGGACTGAGGATCTCG-CT-TC--GGCTAGGATGCTGGCGTAATGGCTGTAAGCGGCCCGTCTTGAAACAC-GG--------------------------------------------ACCAAGGAGTCTAACATCTATGCGAGTGTTTGGGTGTCAAACCCATGCGCGTAA--TGAAAGTG-AACGGAGGTGGGAA-CCCTCAC-GGGTGCACCATCG-ACCGATCCTG--ATGTCTTCGGAT-GGATTTGAGTAAG-AGCATAG-CTGTTGGGA-CCCGAAAGATGGTGAACTATGCCTGAATAGGGTGAAGCCAGAGG-AAACTCTGGTGGAGGCTCGCAGCGG--------------------------------------------------------------TTCTGAC-GTGCA--------------------------------------------------------------------------------AATCGATCGTCAA--------------ATTTGGGTATA-GGGGCGAAAGACTAATC----------------------------------------------------------------------------------------------------------GAACCAT--------------------------------CTAGTAGCTGGTTCCTGCCGAAGTTTCCCTCAGGATAGCAGTAACGTATTCAGTTTTATGAGGTAAAGCGAATGATTAGAGGCCTTGGGGTTGAAACAACCTTAACCTATTCTCAAACTTTAAATATGTAAGAAGTCC-TTGTTACTTAGTTGAACGTGGACACTTGAATGTACCGTTACTAGTGGGCCATTTTTGGTAAGCAGAACTGGCGATGCGGGATGAACCGAACGCGATGTTAAGGTGCCGGAATGCACGCTCATCAGACACCACAAAAGGTGTTAGTTCATCTAGACAGCAGGACGGTGGCCATGGAAGTCGGAATCCGCTAAGGAGTGTGTAACAACTCACCTGCCGAATGAACTAGCCCTGAAAATGGATGGCGCTCAAGCGTGCTACCCATACATCGCCGCCAGGGTAGATACGATGCCCTGGCGAGTAGGCAGGCGTGGAGGCCCGT----------------------------------------------

B.si.OQ845 --------------------------------------------------------------------------------------------------------------------------------------------------------------------------------------------------------------------------------------------------------------------------------------------------------------------------------------------------------------------------------------------------------------------------------------------------------------------------------------------------------------------------------------------------------------------------------------------------------------------------------------------------------------------------------GGGTAGAAGA--GCAAC-----------------------------------------AGGG-ATTGCCTTAGTAAC--GGCGAGTGAAGCGGCAACAGCTCAAATTTGAAAGCTGGCTCCTTTGGA---GTCCGCGTTGTAATTTGTAGAGGATGATTCGGCAAGGG-CTCCCGCCTAAGTCTCCTGGAACGGAGCGTCATAGAGGGTGA-GAATCCCGTATGCGGTGGGC-TGCCTAAGCCATGTGAATCTCCTTCGACGAGTCGAGTTGTTTGGGAATGCAGCTCTAAATGGGAGGTAAATTTCTTCTAAAGCTAAATACCGGCCAGAGACCGATAGCGCACAAGTAGAGTGATCGAAAGATGAAAAGCACTTTG-GAAAGAGAGTTAAAAAGTACGTGAAATTGTTGAAAGGGAAGCGCTTGCAGCCAGACTTGTCCGCAGTTGCTCAGCCGGTCTCC--TGACCGGTGTACTCTTCTGCGGCCAGGCCAGCATCAGTTCGGGCGGTCGGATAAAGACCTCGGGAATGTAGCTCCTCT---CGGGGAG-TGTTATAGCCCGGGGTGGAATGCGGCCAGCCTGGACTGAGGATCTCG-CT-TC--GGCTAGGATGCTGGCGTAATGGCTGTAAGCGGCCCGTCTTGAAACAC-GG--------------------------------------------ACCAAGGAGTCTAACATCTATGCGAGTGTTTGGGTGTCAAACCCATGCGCGTAA--TGAAAGTG-AACGGAGGTGGGAA-CCCTCAC-GGGTGCACCATCG-ACCGATCCTG--ATGTCTTCGGAT-GGATTTGAGTAAG-AGCATAG-CTGTTGGGA-CCCGAAAGATGGTGAACTATGCCTGAATAGGGTGAAGCCAGAGG-AAACTCTGGTGGAGGCTCGCAGCGG--------------------------------------------------------------TTCTGAC-GTGCA--------------------------------------------------------------------------------AATCGATCGTCAA--------------ATTTGGGTATA-GGGGCGAAAGACTAATC----------------------------------------------------------------------------------------------------------GAACCAT--------------------------------CTAGTAGCTGGTTCCTGCCGA---------------------------------------------------------------------------------------------------------------------------------------------------------------------------------------------------------------------------------------------------------------------------------------------------------------------------------------------------------------------------------------------------------------------------------------------------GT----------------------------------------------

B.do.DQ678 -------------------------------------------------------------------------------------------------------------------------------------------------------------------------------------------------------------------------------------------------------------------------------------------------------------------------------------------------------------------------------------------------------------------------------------------------------------------------------------------------------------------------------------------------------------------------------------------------------------------------------------------------CCCGCTGAACTTAAGCATATCAATAAGCGGAGGAAAAGAAA--CCAAC-----------------------------------------AGGG-ATTGCCTTAGTAAC--GGCGAGTGAAGCGGCAACAGCTCAAATTTGAAAGCTGGCTCCTTTGGA---GTCCGCGTTGTAATTTGTAGAGGATGATTCGGCAAGGG-CTCCCGCCTAAGTCTCCTGGAACGGAGCGTCATAGAGGGTGA-GAATCCCGTATGCGGTGGGC-TGCCTAAGCCATGTGAATCTCCTTCGACGAGTCGAGTTGTTTGGGAATGCAGCTCTAAATGGGAGGTAAATTTCTTCTAAAGCTAAATACCGGCCAGAGACCGATAGCGCACAAGTAGAGTGATCGAAAGATGAAAAGCACTTTG-GAAAGAGAGTTAAAAAGTACGTGAAATTGTTGAAAGGGAAGCGCTTGCAGCCAGACTTGTCCGCAGTTGCTCAGCCGGTCTCC--TGACCGGTGTACTCTTCTGCGGCCAGGCCAGCATCAGTTCGGGCGGTCGGATAAAGACCTCGGGAATGTAGCTCCTCT---CGGGGAG-TGTTATAGCCCGGGGTGGAATGCGGCCAGCCTGGACTGAGGATCTCG-CT-TC--GGCTAGGATGCTGGCGTAATGGCTGTAAGCGGCCCGTCTTGAAACAC-GG--------------------------------------------ACCAAGGAGTCTAACATCTATGCGAGTGTTTGGGTGTCAAACCCATGCGCGTAA--TGAAAGTG-AACGGAGGTGGGAA-CCCTCAC-GGGTGCACCATCG-ACCGATCCTG--ATGTCTTCGGAT-GGATTTGAGTAAG-AGCATAG-CTGTTGGGA-CCCGAAAGATGGTGAACTATGCCTGAATAGGGTGAAGCCAGAGG-AAACTCTGGTGGAGGCTCGCAGCGG--------------------------------------------------------------TTCTGAC-GTGCA--------------------------------------------------------------------------------AATCGATCGTCAA--------------ATTTGGGTATA-GGGGCGAAAGACTAATC----------------------------------------------------------------------------------------------------------GAACCAT--------------------------------CTAGTAGCTGGTTCCTGCCGAAGTTTCCCTCAGGATAGCAGTAACGTATTCAGTTTTATGAGGTAAAGCGAATGATTAGAGGCCTTGGGGTTGAAACAACCTTAACCTATTCTCAAACTTTAAATATGTAAGAAGTCC-TTGTTACTTAGTTGAACGTGGACACTTGAATGTACCGTTACTAGTGGGCCATTTTTGGTAAGCAGAACTGGCGATGCGGGATGAACCGAACGCGATGTTAAGGTGCCGGAATGCACGCTCATCAGACACCACAAAAGGTGTTAGTTCATCTAGACAGCAGGACGGTGGCCATGGAAGTCGGAATCCGCTAAGGAGTGTGTAACAACTCACCTGCCGAATGAACTAGCCCTGAAAATGGATGGCGCTCAAGCGTGCTACCCATACATCGCCGCCAGGGTAGATACGATGCCCTGGCGAGTAGGCAGGCGTGGAGGCCCGT----------------------------------------------

Hy.mo.FJ16 -----------------------------------------------------------------------------------------------------------------------------------------------------------------------------------------------------------------------------------------------------------------------------------------------------------------------------------------------------------------------------------------------------------------------------------------------------------------------------------------------------------------------------------------------------------------------------------------------------------------------------------------------------------T--AACATATCAATAAGCGGAGGAAAAGAAA--CCAAC-----------------------------------------AGGG-ATTGCCCTAGTAAC--GGCGAGTGAAGCGGCAACAGCTCAAATTTGAAATCTGGC-CTTTCAG----GTCCGAGTTGTAATTTGTAGAGGATGCTTCGGCGTTGG-ACCCGACCTAAGTTCCTTGGAACAGGACGTCACAGAGGGTGA-GAATCCCGTACGTGGCCGGT-GTCCCTCGCCATGTGAAGCTCCTTCGACGAGTCGAGTTGTTTGGGAATGCAGCTCTAAATGGGAGGTAAATTTCTTCTAAAGCTAAATACTGGCCAGAGACCGATAGCGCACAAGTAGAGTGATCGAAAGATGAAAAGCACTTTG-GAAAGAGAGTTAAAAAGTACGTGAAATTGTTGAAAGGGAAGCGCTTGCAACCAGACCTGCCCGCGGTCGCTCATCCAGGCTTC--TGCCTGGTGCACTCTTCCGCGGTCAGGCCAGCATCGGTTCGGGCGGTCGGACAAAGGCGGCGGGAATGTGGCTCCCTT---CGGGGAG-TGTTATAGCCCGCCGTGCAATGCGGCCAGTCCGGACCGAGGTCCGCG-CT-TC--GGCTAGGATGCTGGCGTAATGGTTGTAAGCGGCCCGTCTTGAAACAC-GG--------------------------------------------ACCAAGGAGTCTAACATCTGTGCGAGTGTTTGGGTGTCAAGCCCGGACGCGTAA--TGAAAGTG-AACGGAGGCGGGAA-CCTTT---GGGTGCACCGTCG-ACCGATCCTG--ATGTCTTCGGAT-GGATTTGAGTAAG-AGCATAG-CTGTTGGGA-CCCGAAAGATGGTGAACTATGCCTGAATAGGGTGAAGCCAGAGG-AAACTCTGGTGGAGGCTCGCAGCGG--------------------------------------------------------------TTCTGAC-GTGCA--------------------------------------------------------------------------------AATCGATCGTCAA--------------ATTTGGGCATA-GGGGCGAAAGACTAATC----------------------------------------------------------------------------------------------------------GAACTAT--------------------------------CTAGTAGCTGGTTCCTGCCGAAGTTTCCCTCAGGATAGCAGTAACGTTTTCAGTTTTATGAGGTAAAGCGAATGATTAGAGGCCTGGGGGTTGAAACAACCTTCACCTATTCTCAAACTTTAAATATGTAAGAAGTCC-TTGTTACTTAGTTGAACGTGGACATTTGAATGTACCGTTACTAGTGGGCCATTTTTGGTAAGCAGAACTGGCGATGCGGGATGAACCGAACGCGGGGTTAAGGTGCCGGAATGCACGCTCATCAGACACCACAAAAGGTGTTAGTTCATCTAGACAGCAGGACGGTGGCCATGGAAGTCGGAATCCGCTAAGGAGTGTGTAACAACTCACCTGCCGAATGAACTAGCCCTGAAAATGGATGGCGCTCAAGCGTGCTACCCATACCCCGCCGCCGGGGCAAAAGTTACGCCCCGGCGAGTAGGCAGGCGTGGAGGCCCGTGACGAAGCCTTGGGGGTGACCCCGGGTAGAACGGCCTCTA------

Hy196 -----------------------------------------------------------------------------------------------------------------------------------------------------------------------------------------------------------------------------------------------------------------------------------------------------------------------------------------------------------------------------------------------------------------------------------------------------------------------------------------------------------------------------------------------------------------------------------------------------------------------------------------------------------TTAAGCATATCAATAAGCGGAGGAAAAGAAA--CCAAC-----------------------------------------AGGG-ATTGCCCTAGTAAC--GGCGAGTGAAGCGGCAACAGCTCAAATTTGAAATCTGGC-CTTTCAG----GTCCGAGTTGTAATTTGTAGAGGATGCTTCGGCGTTGG-ACCCGACCTAAGTTCCTTGGAACAGGACGTCACAGAGGGTGA-GAATCCCGTACGTGGCCGGT-GTCCCTCGCCATGTGAAGCTCCTTCGACGAGTCGAGTTGTTTGGGAATGCAGCTCTAAATGGGAGGTAAATTTCTTCTAAAGCTAAATACTGGCCAGAGACCGATAGCGCACAAGTAGAGTGATCGAAAGATGAAAAGCACTTTG-GAAAGAGAGTTAAAAAGTACGTGAAATTGTTGAAAGGGAAGCGCTTGCAACCAGACCTGCCCGCGGTCGCTCATCCAGGCTTC--TGCCTGGTGCACTCTTCCGCGGTCAGGCCAGCATCGGTTCGGGCGGTCGGACAAAGGCGGCGGGAATGTGGCTCCCTT---CGGGGAG-TGTTATAGCCCGCCGTGCAATGCGGCCAGTCCGGACCGAGGTCCGCG-CT-TC--GGCTAGGATGCTGGCGTAATGGTTGTAAGCGGCCCGTCTTGAAACAC-GG--------------------------------------------ACCAAGGAGTCTAACATCTGTGCGAGTGTTTGGGTGTCAAGCCCGGACGCGTAA--TGAAAGTG-AACGGAGGCGGGAA-CCTTT---GGGTGCACCGTCG-ACCGATCCTG--ATGTCTTCGGAT-GGATTTGAGTAAG-AGCATAG-CTGTTGGGA-CCCGAAAGATGGTGAACTATGCCTGAATAGGGTGAAGCCAGAGG-AAACTCTGGTGGAGGCTCGCAGCGG--------------------------------------------------------------TTCTGAC-GTGCA--------------------------------------------------------------------------------AATCGATCGTCAA--------------ATTTGGGCATA-GGGGCGAAAGACTAATC----------------------------------------------------------------------------------------------------------GAACTAT--------------------------------CTAGTAGCTGGTTCCTGCCGAAGTTTCCCTCAGGATAGCAGTAACGTTTTCAGTTTTATGAGGTAAAGCGAATGATTAGAGGCCTGGGGGTTGAAACAACCTTCACCTATTCTCAAACTTTAAATATGTAAGAAGTCC-TTGTTACTTAGTTGAACGTGGACATTTGAATGTACCGTTACTAGTGGGCCATTTTTGGTAAGCAGAACTGGCGATGCGGGATGAACCGAACGCGGGGTTAAGGTGCCGGAATGCACGCTCATCAGACACCACAAAAGGTGTTAGTTCATCTAGACAGCAGGACGGTGGCCATGGAAGTCGGAATCCGCTAAGGAGTGTGTAACAACTCACCTGCCGAATGAACTAGCCCTGAAAATGGATGGCGCTCAAGCGTGCTACCCATACCCCGCCGCCGGGGCAAAAGTTACGCCCCGGCGAGTAGGCAGGCGTGGAGGCCCGTGACGAAGCCTTGGGGGTGACCCCGGGTAGAACGGCCTCTAGTG---

He.me.OQ17 -------------------------------------------------------------------------------------------------------------------------------------------------------------------------------------------------------------------------------------------------------------------------------------------------------------------------------------------------------------------------------------------------------------------------------------------------------------------------------------------------------------------------------------------------------------------------------------------------------------------------------------------------------------------------------------------------------------------------------------------------------AGTAACCGGGCGAGTGAAGCGGCAACAGCTCAAATTTGAAATCTGGCTCCCTTTGGGG-GTCCGAGTTGTAATTTGCAGAGGGTGCTTTGGCGTTGG-TGGCGGTCTAAGTTCCTTGGAACAGGACATCGCAGAGGGTGA-GAATCCCGTTTGTGGTCGCC-TGCCTTCGCCGTGTAAAGCCCCTTCGACGAGTCGAGTTGTTTGGGAATGCAGCTCTAAATGGGAGGTAAATTTCTTCTAAAGCTAAATACTGGCCAGAGACCGATAGCGCACAAGTAGAGTGATCGAAAGATGAAAAGCACTTTG-GAAAGAGAGTCAAACAGCACGTGAAATTGTTGAAAGGGAAGCGCTTGCCGCCAGACTTGCCCGTAGTTGCTCACCCAGCCTTT--TGGCTGGTGCATTCTTCTGCGGGCAGGCCAGCATCAGTTTGGGCGGTCGGATAAAGGCCTCTGGCATGTTCCTTCCTT---CGGGTTGGCCATATAG-GGGAGGTGCAATGCGACCAGCCCGGACTGAGGTCCGCG-CT-TT--TGCTAGGATGCTGGCGTAATGGCTGTAAGCGGCCCGTCTTGAAACACGGA--------------------------------------------CCCAAGGA-------------------------------------------------------------------------------------------------------------------------------------------------------------------------------------------------------------------------------------------------------------------------------------------------------------------------------------------------------------------------------------------------------------------------------------------------------------------------------------------------------------------------------------------------------------------------------------------------------------------------------------------------------------------------------------------------------------------------------------------------------------------------------------------------------------------------------------------------------------------------------------------------------------------------------------------------------------------------------------------------------------------------------------------------------------------------------------------------------------------------

He.ch.ON55 ----------------------------------------------------------------------------------------------------------------------------------------------------------------------------------------------------------------------------------------------------------------------------------------------------------------------------------------------------------------------------------------------------------------------------------------------------------------------------------------------------------------------------------------------------------------------------------------------------------------------------------------------------------------------------------------------------------------------------------------------------------------GCGAGTGAAGCGGCAACAGCTCAAATTTGAAATCCGTCTCCCTCTGGGGAGTCAGAGTTGTAATTTGCAGAGGGTGCTTTGGCGTTGG-TGGCGGTCTAAGTTCCTTGGAACAGGACATCGCAGAGGGTGA-GAATCCCGTTTGTGGTCGCT-TGCCTTCGCCGTGTAAAGCCCCTTCGACGAGTCGAGTTGTTTGGGAATGCAGCTCTAAATGGGAGGTAAATTTCTTCTAAAGCTAAATACTGGCCAGAGACCGATAGCGCACAAGTAGAGTGATCGAAAGATGAAAAGCACTTTG-GAAAGAGAGTCAAACAGCACGTGAAATTGTTGAAAGGGAAGCGCTTGCAGCCAGACTTGCCCGTAGTTGCTCACCCAGGCTTT--TGCCTGGGGCATTCTTCTGCGGGCAGGCCAGCATCAGTTTGGGCGGTCGGATAAAGGCCTCTGTCACGTATCTTCCTT---CGGGATGACCTTATAG-GGGAGGCGTCATGCGACCAGCCCGGACTGAGGTCCGCG-CT-TC--TGCTAGGATGCTGGCGTAATGGCTGTAAGCGGCCCGTCTTGAAACAC-GG--------------------------------------------ACCAAGGAGTCTAACATCTATGCGAGTGTTTGGGTGTCAAGCCCGAGCGCGTAA--TGAAAGTG-AACGGAGGTGGGAA-CCCTC---GGGTGCACCATCG-ACCGATCCTG--AAGTCTTCGGAT-GGATTTGAGTAAG-AGCATAG-CTGTTGGGA-CCCGAAAGATGGTGAACTATGCTTGAATAGGGTGAAGCCAGAGG-AAACTCTGGTGGAGGCTCGCAGCGG--------------------------------------------------------------TTCTGAC-GTGCA--------------------------------------------------------------------------------AATCGATCGTCAA--------------ATTTGGGCATA-GGGGCGAA-GAC------------------------------------------------------------------------------------------------------------------------------------------------------------------------------------------------------------------------------------------------------------------------------------------------------------------------------------------------------------------------------------------------------------------------------------------------------------------------------------------------------------------------------------------------------------------------------------------------------------------------------------------------------------------------------

He.j.OQ172 ----------------------------------------------------------------------------------------------------------------------------------------------------------------------------------------------------------------------------------------------------------------------------------------------------------------------------------------------------------------------------------------------------------------------------------------------------------------------------------------------------------------------------------------------------------------------------------------------------------------------------------------------------------------------------------------------ACC-----------------------------------------ACTA-AAAATTTTAGTAACCGGGCGA-TGAAGCGGCAATAGCTCAAATTTGAAATCTGGCTCCCTCTGGGG-GTCCGAGTTGTAATTTGCAGAGGGTGCTTTGGCGTTGG-TGGCGGTCTAAGTTCCTTGGAACAGGACATCGCAGAGGGTGA-GAATCCCGTTTGTGGTCGCA-TGCCTTCGCCGTGTAAAGCCCCTTCGACGAGTCGAGTTGTTTGGGAATGCAGCTCTAAATGGGAGGTAAATTTCTTCTAAAGCTAAATACTGGCCAGAGACCGATAGCGCACAAGTAGAGTGATCGAAAGATGAAAAGCACTTTG-GAAAGAGAGTCAAACAGCACGTGAAATTGTTGAAAGGGAAGCGCTTGCAGCCAGACTTGCCCGTAGTTGCTCACCCAGGCTTT--TGCCCGGGGCATTCTTCTGCGGGCAGGCCAGCATCAGTTTGGGCGGTCGGATAAAGGCCTCTGTCACGTATCTTCCTT---CGGGATGACCTTATAG-GGGAGGCGACATGCGACCAGCCCGGACTGAGGTCCGCG-CT-TC--TGCTAGGATGCTGGCGTAATGGCTGTAAGCGGCCCGTCTTGAAACACGAC--------------------------------------------CCCAAGGAGTAAAACAC-------------------------------------------------------------------------------------------------------------------------------------------------------------------------------------------------------------------------------------------------------------------------------------------------------------------------------------------------------------------------------------------------------------------------ACAAAGA--------------------------------------------------------------------------------------------------------------------------------------------------------------------------------------------------------------------------------------------------------------------------------------------------------------------------------------------------------------------------------------------------------------------------------------------------------------------------------------------------------------------------------------------------------------------------------------------------------------------------------------------------------------------------------

L.cr.DQ678 ---------------------------------------------------------------------------------------------------------------------------------------------------------------------------------------------------------------------------------------------------------------------------------------------------------------------------------------------------------------------------------------------------------------------------------------------------------------------------------------------------------------------------------------------------------------------------------------------------------------------------------------------------------ACTTAAGCATATCAATAAGCGGAGGAAAAGAAA--CCAAC-----------------------------------------AGGG-ATTGCCCTAGTAAC--GGCGAGTGAAGCGGCAACAGCTCAAATTTGAAATCTGGCTCCTTCGGG---GTCCGAGTTGTAATTTGTAGAGGGTGCTTTGGCGTTGG-CTGTGGTCTAAGTTCCTTGGAACAGGACGTCACAGAGGGTGA-GAATCCCGTACGTGGCCGCC-AGCCTCCGCCGTGTAAAGCCCCTTCGACGAGTCGAGTTGTTTGGGAATGCAGCTCTAAATGGGAGGTAAATTTCTTCTAAAGCTAAATATTTGCCAGAGACCGATAGCGCACAAGTAGAGTGATCGAAAGATGAAAAGCACTTTG-GAAAGAGAGTCAAAAAGCACGTGAAATTGTTGAAAGGGAAGCGCTTGCAGCTAGACGTGCCTTGGGTTGATCAGCCGGACTTT--TGCCCGGTGCACTCTTCCCTTGGCAGGCCAGCATTAGTTTGGGCGGCTGGATAAAGGTCTGTTAAACGTGACTTCCTT---CGGGGAGAGCTTATAG-GGCAGACGACATGCAGCCAGCCTGAACTGAGGTCCGCG-CA-TC--TGCTAGGATGCTGGCATAATAGCTGTAAGCGGCCCGTCTTGAAACAC-GG--------------------------------------------ACCAAGGAGTCTAACATCTATGCGAGTGTTTGGGTGTCAAGCCCGAGCGCGTAA--TGAAAGTA-AACGGAGGTGGGAA-CCTTTTA--GGCGCACCATCG-ACCGATCCTG--ATGTCTTCGGAT-GGATTTGAGTAAG-AGCATAG-CTGTTGGGA-CCCGAAAGATGGTGAACTATGCCTGAATAGGGTGAAGCCAGAGG-AAACTCTGGTGGAGGCTCGCAGCGG--------------------------------------------------------------TTCTGAC-GTGCA--------------------------------------------------------------------------------AATCGATCGTCAA--------------ATTTGGGCATA-GGGGCGAAAGACTAATC----------------------------------------------------------------------------------------------------------GAACTAT--------------------------------CTAGTAGCTGGTTCCTGCCGAAGTTTCCCTCAGGATAGCAGTAACGTTTTCAGTTTTATGAGGTAAAGCGAATGATTAGAGGCCTGGGGGTTGAAACAACCTTCACCTATTCTCAAACTTTAAATATGTAAGAAGTCC-TTGTTGCTTAATTGAACGTGGACATTTGAATGTATCGTTACTAGTGGGCCATTTTTGGTAAGCAGAACTGGCGATGCGGGATGAACCGAACGTGGGGTTAAGGTGCCAGAATGCACGCTCATCAGACACCACAAAAGGTGTTAGTTCATCTAGACAGCAGGACGGTGGCCATGGAAGTCGGAATCCGCTAAGGAGTGTGTAACAACTCACCTGCCGAATGAACTAGCCCTGAAAATGGATGGCGCTTAAGCGTGTTACCTATACCCCGCCGCCAGGGCAGAATTTA-------------------------------------------------------------------------------

L.ar.DQ782 --------------------------------------------------------------------------------------------------------------------------------------------------------------------------------------------------------------------------------------------------------------------------------------------------------------------------------------------------------------------------------------------------------------------------------------------------------------------------------------------------------------------------------------------------------------------------------------------------------------------------------------------------------------AGCATATCAATAAGCGGAGGAAAAGAAA--CCAAC-----------------------------------------AGGG-ATTGCCCTAGTAAC--GGCGAGTGAAGCGGCAACAGCTCAAATTTGAAATCTGGCTCCTTTGGG---GTCCGAGTTGTAATTTGTAGAGGGTGCTTTGGCGTCGG-TTGTGGTCTAAGTTCCTTGGAACAGGACGTCACAGAGGGTGA-GAATCCCGTACGTGGCCGCC-AGCCTTCGCCGTGTAAAGCCCCTTCGATGAGTCGAGTTGTTTGGGAATGCAGCTCTAAATGGGAGGTAAATTTCTTCTAAAGCTAAATATTTGCCAGAGACCGATAGCGCACAAGTAGAGTGATCGAAAGATGAAAAGCACTTTG-GAAAGAGAGTCAAAAAGCACGTGAAATTGTTGAAAGGGAAGCGCTTGCAGCTAGACGTGCCTTGGGTTGATCAGCCGGACTTT--TGCCCGGTGCACTCTTCCTTTGGCAGGCCAGCATCAGTTTGGGCGGCTGGATAAAGGTCTGTTAAACGTGACTTCCTT---CGGGGAGAGCTTATAG-GGCAGACGACATGCAGCCAGCCTGAACTGAGGTCCGCG-CA-TC--TGCTAGGATGCTGGCATAATAGCTGTAAGCGGCCCGTCTTGAAACAC-GG--------------------------------------------ACCAAGGAGTCTAACATCTATGCGAGTGTTTGGGTGTCAAGCCCGAGCGCGTAA--TGAAAGTA-AACGGAGGTGGGAA-CCTTTTA--GGTGCACCATCG-ACCGATCTTG--ATGTCTTCGGAT-GGATTTGAGTAAG-AGCATAG-CTGTTGGGA-CCCGAAAGATGGTGAACTATGCCTGAATAGGGTGAAGCCAGAGG-AAACTCTGGTGGAGGCTCGCAGCGG--------------------------------------------------------------TTCTGAC-GTGCA--------------------------------------------------------------------------------AATCGATCGTCAA--------------ATTTGGGCATA-GGGGCGAAAGACTAATC----------------------------------------------------------------------------------------------------------GAACTAT--------------------------------CTAGTAGCTGGTTCCTGCCGAAGTTTCCCTCAGGATAGCAGTAACGTTTTCAGTTTTATGAGGTAAAGCGAATGATTAGAGGCCTGGGGGTTGAAACAACCTTCACCTATTCTCAAACTTTAAATATGTAAGAAGTCC-TTGTTACTTAATTGAACGTGGACATTTGAATGTATCGTTACTAGTGGGCCATTTTTGGTAAGCAGAACTGGCGATGCGGGATGAACCGAACGTGGGGTTAAGGTGCCAGAATGCACGCTCATCAGACACCACAAAAGGTGTTAGTTCATCTAGACAGCAGGACGGTGGCCATGGAAGTCGGAATCCGCTAAGGAGTGTGTAACAACTCACCTGCCGAATGAACTAGCCCTGAAAATGGATGGCGCTTAAGCGTGTTACCTATACCCCGCCGCCAGGGCAGAATTTATGCCCTGGCGAGTAGGCAGGCG---------------------------------------------------------

Pl.iq.NG_0 --------------------------------------------------------------------------------------------------------------------------------------------------------------------------------------------------------------------------------------------------------------------------------------------------------------------------------------------------------------------------------------------------------------------------------------------------------------------------------------------------------------------------------------------------------------------------------------------------------------------------------------------------------------------------------------------------------------------------------------------------GCCCCAGTAAC--GGCGAGTGAAGCGGCAACAGCTCAAATTTGAAATCTGGCCCTTGCAGG---GTCCGAGTTGTAATTTGCAGAGGGTGCTTTGGCGTCGG-CTGTGGTCTAAGTTCCTTGGAACAGGACGTCGCAGAGGGTGA-GAATCCCGTATGTGGCCGCC-AGTCTTCGCCGTGTAAAGCCCCTTCGACGAGTCGAGTTGTTTGGGAATGCAGCTCTAAATGGGAGGTAAATTTCTTCTAAAGCTAAATATTGGCCAGAGACCGATAGCGCACAAGTAGAGTGATCGAAAGATGAAAAGCACTTTG-GAAAGAGAGTCAAAAAGCACGTGAAATTGTTGAAAGGGAAGCGCTTGCAGCCAGACTTGCCCGCAGTTGCTCATCCGGGCTCG--CGCCCGGTGCACTCTTCTGCGGGCAGGCCAGCATCAGTCCGGGCGGTCGGATAAAGGTCTGCGGCATGTACCTCCCCT---CGGGGAGGACTTATAG-GGTAGACGACATGCGACCAGCCTGGATTGAGGTCCGCG-CA-TC--TGCTAGGATGCTGGCGTAATGGCTGTAAGCGGCCCGTCTTGAAACAC-GG--------------------------------------------ACCAAGGAGTCTAACATCTATGCGAGTGTTTGGGTGTCAAGCCCAAGCGCGCAA--TGAAAGTG-AACGGAGGTGGGAA-CCCCTCG-GGGCGCACCATCG-ACCGATCCTG--ATGTCTTCGGAT-GGATTTGAGTAAG-AGCATAG-CTGTTGGGA-CCCGAAAGATGGTGAACTATGCCTGAATAGGGTGAAGCCAGAGG-AAACTCTGGTGGAGGCTCGCAGCGG--------------------------------------------------------------TTCTGAC-GTGCA--------------------------------------------------------------------------------AATCGATCGTCAA--------------ATTTGGGCATA-GGGGCGAAAGACTAATC----------------------------------------------------------------------------------------------------------GAACTAT--------------------------------CTAGTAGCTGG-------------------------------------------------------------------------------------------------------------------------------------------------------------------------------------------------------------------------------------------------------------------------------------------------------------------------------------------------------------------------------------------------------------------------------------------------------------------------------------------------------------

Po2.1 ---------------------------------------------------------------------------------------------------------------------------------------------------------------------------------------------------------------------------------------------------------------------------------------------------------------------------------------------------------------------------------------------------------------------------------------------------------------------------------------------------------------------------------------------------------------------------------------------------------------------------------------------------------------------------GCGGAGGAAAAGAAA--CCAAC-----------------------------------------CGGG-ATTGCCTCAGTAAC--GGCGAGTGAAGCGGCATCAGCTCAAATTTGAAATCTGGCTCCTCTGGG---GCCCGAGTTGTAATTTGCAGAGGGCGCTTTGGCGTTGG-CAGCGGTCTAAGTTCCTTGGAACAGGACGTCACAGAGGGTGA-GAATCCCGTACGTGGTCGCT-AGCGTTCGCCGTGTAAAGCCCCTTCGACGAGTCGAGTTGTTTGGGAATGCAGCTCTAAATGGGAGGTAAATTTCTTCTAAAGCTAAATACTGGCCAGAGACCGATAGCGCACAAGTAGAGTGATCGAAAGATGAAAAGCACTTTG-GAAAGAGAGTCAAACAGCACGTGAAATTGTTGAAAGGGAAGCGCTTGCAGCCAGACTTGCCTGTAGTTGCTCATCCAGGCTTT--TGCCCGGTGCACTCTTCTGCAGGCAGGCCAGCATCAGTTTGGGCGGTTGGATAAAGGCCTCTGTCACGTACCTCCCTT---CGGGGAGGCCTTATAG-GGGAGGCGGCATGCAACCAGCCTGGACTGAGGTCTGCG-CA-TC--TGCTAGGATGCTGGCGTAATGGCTGTAAGCGGCCCGTCTTGAAACAC-GG--------------------------------------------ACCAAGGAGTCTAACATCTATGCGAGTGTTTGGGTGTCAAGCCCGAGCGCGGAA--TGAAAGTG-AACGGAGGTGGGAT-CCCTTGC-GGGTGCACCATCG-ACCGATCCTG--ATGTCTTCGGAA-GGATTTGAGTAAG-AGCATGG-CTGTTGGGA-CCCGAAAGATGGTGAACTATGCTTGAATAGGGTGAAGCCAGAGG-AAACTCTGGTGGAGGCTCGCAGCGG--------------------------------------------------------------TTCTGAC-GTGCA--------------------------------------------------------------------------------AATCGATCGTCAA--------------ATTTGGGCATA-GGGGCGAAAGACTAATC----------------------------------------------------------------------------------------------------------GAACTAT--------------------------------CTAGTAGCTGG-------------------------------------------------------------------------------------------------------------------------------------------------------------------------------------------------------------------------------------------------------------------------------------------------------------------------------------------------------------------------------------------------------------------------------------------------------------------------------------------------------------

Po.nv.zela -----------------------------------------------------------------------------------------------------------------------------------------------------------------------------------------------------------------------------------------------------------------------------------------------------------------------------------------------------------------------------------------------------------------------------------------------------------------------------------------------------------------------------------------------------------------------------------------------------------------------------------------------------------------------------GGAGGAAAAGAAA--CCAAC-----------------------------------------CGGG-ATTGCCTCAGTAAC--GGCGAGTGAAGCGGCATCAGCTCAAATTTGAAATCTGGCTCCTCTGGG---GCCCGAGTTGTAATTTGCAGAGGGCGCTTTGGCGTTGG-CAGCGGTCTAAGTTCCTTGGAACAGGACGTCACAGAGGGTGA-GAATCCCGTACGTGGTCGCT-AGCGTTCGCCGTGTAAAGCCCCTTCGACGAGTCGAGTTGTTTGGGAATGCAGCTCTAAATGGGAGGTAAATTTCTTCTAAAGCTAAATACTGGCCAGAGACCGATAGCGCACAAGTAGAGTGATCGAAAGATGAAAAGCACTTTG-GAAAGAGAGTCAAACAGCACGTGAAATTGTTGAAAGGGAAGCGCTTGCAGCCAGACTTGCCTGTAGTTGCTCATCCAGGCTTT--TGCCCGGTGCACTCTTCTGCAGGCAGGCCAGCATCAGTTTGGGCGGTTGGATAAAGGCCTCTGTCACGTACCTCCCTT---CGGGGAGGCCTTATAG-GGGAGGCGGCATGCAACCAGCCTGGACTGAGGTCTGCG-CA-TC--TGCTAGGATGCTGGCGTAATGGCTGTAAGCGGCCCGTCTTGAAACAC-GG--------------------------------------------ACCAAGGAGTCTAACATCTATGCGAGTGTTTGGGTGTCAAGCCCGAGCGCGGAA--TGAAAGTG-AACGGAGGTGGGAT-CCCTTGC-GGGTGCACCATCG-ACCGATCCTG--ATGTCTTCGGAA-GGATTTGAGTAAG-AGCATGG-CTGTTGGGA-CCCGAAAGATGGTGAACTATGCTTGAATAGGGTGAAGCCAGAGG-AAACTCTGGTGGAGGCTCGCAGCGG--------------------------------------------------------------TTCTGAC-GTGCA--------------------------------------------------------------------------------AATCGATCGTCAA--------------ATTTGGGCATA-GGGGCGAAAGACTAATC----------------------------------------------------------------------------------------------------------GAACTAT--------------------------------CTAGT-------------------------------------------------------------------------------------------------------------------------------------------------------------------------------------------------------------------------------------------------------------------------------------------------------------------------------------------------------------------------------------------------------------------------------------------------------------------------------------------------------------------

Co.palm.DQ ----------------------------------------------------------------------------------------------------------------------------------------------------------------------------------------------------------------------------------------------------------------------------------------------------------------------------------------------------------------------------------------------------------------------------------------------------------------------------------------------------------------------------------------------------------------------------------------------------------------------------------------------------------------------------------------------------------------------------------------------------------------------------------------------------------------------------------------------------------------------------------------------------------------------------------------------------------------------------------------------------------------------------------------------------------------------------------------------------------------------------------------------------------------------------------------------------------------AGATAAAGGTCTCTGTCGTGTACCTCTCTT---CGGGGAGGCTGTATAG-GGGAGGCGTCATACAACCAGCCTGGACTGAGGTCCGCG-CATTT--TGCTAGGATGCTGGCGTAATGGCTGTAAGCGGCCTGTCTTGAAACAC-GG--------------------------------------------ACCAAGGAGTCTAACATCTATGCGAGTGTTTGGGTGTCAAGCCCGAACGCGTAA--TGAAAGTG-AACGGAGGTGGGAA-NCCTTAC-GGGTGCACCATCG-ACCGATCCTG--ATGTCTTCGGAT-GGATTTGAGTAAG-AGCATGG-CTGTTGGGA-CCCGAAAGATGGTGAACTATGCTTGAATAGGGTGAAGCCAGAGG-AAACTCTGGTGGAGGCTCGCAGCGG--------------------------------------------------------------TTCTGAC-GTGCA--------------------------------------------------------------------------------AATCGATCGTCAA--------------ATTTGGGCATA-GGGGCGAAAGACTAATC----------------------------------------------------------------------------------------------------------GAACTAT--------------------------------CTAGTAGCTGGTTCCTGCCGAAGTTTCCCTCAGGATAGCAGTAACGTATTCAGTTTTATGAGGTAAAGCGAATGATTAGAGGCCTGGGGGTTGAAACAACCTTCACCTATTCTCAAACTTTAAATATGTAAGAAGTCC-TTGTTACTTGATTGAACGTGGACACTTGAATGTACCGTTACTAGTGGGCCATTTTTGGTAAGCAGAACNGGCGATGCGGGATGAACCGAACGCGGGGTTAAGGTGCCAGAATGCACGCTCATCAGACACCACAAAAGGTGTTAGTTCATCTAGACAGCAGGACGGTGGCCATGGAAGTCGGAATCCGCTAAGGAGTGTGTAACAACTCACCTGCCGAATGAACTAGCCCTGAAAATGGATGGCGCTCAAGCGTGTTACCCATACCCCGCCGCCGGGGCAAGATTTAAGCCCCGGCGAGTAGGCAGGCGTGGAGGCTCGTG---------------------------------------------

Pyc.nb.DQ6 -------------------------------------------------------------------------------------------------------------------------------------------------------------------------------------------------------------------------------------------------------------------------------------------------------------------------------------------------------------------------------------------------------------------------------------------------------------------------------------------------------------------------------------------------------------------------------------------------------------------------------------------------------------------------------------------------------------------------------------------------------------------------------TCAGCTCAAATTTGAAATCTGGCTCTTTCAGA---GTCCGAGTTGTAATTTGCAGAGGGCGCTTTGGCGTTGG-CAGCGGTCCAAGTTCCTTGGAACAGGACGTCACAGAGGGTGA-GAATCCCGTACGTGGTCGCT-GGCCTTCGCCGTGTAAAGCCCCTTCGACGAGTCGAGTTGTTTGGGAATGCAGCTCTAAATGGGAGGTAAATTTCTTCTAAAGCTAAATACTGGCCAGAGACCGATAGCGCACAAGTAGAGTGATCGAAAGATGAAAAGCACTTTG-GAAAGAGAGTCAAATAGCACGTGAAATTGTTGAAAGGGAAGCGCTTGCAGCCAGACTTGCCTGTAGTTGCTCATCCGGGCTTT--TGCCCGGTGCACTCTTCTGCGGGCAGGCCAGCATCAGTTTGGGCGGTTGGATAAAGGCCTCTGTCATGTACCTCCTTT---CGGGGAGGCCTTATAG-GGGAGGCGACATGCAACCAGCCTGGACTGAGGTCCGCG-CA-TT--TGCTAGGATGCTGGCGTAATGGCTGTAAGCGGCCCGTCTTGAAACAC-GG--------------------------------------------ACCAAGGAGTCTAACATCTATGCGAGTGTTTGGGTGTCAAGCCCGAACGCGTAA--TGAAAGTG-AACGGAGGTGGGAT-CCCTTAA-GGGTGCACCATCG-ACCGATCCTG--ATGTCTTCGGAT-GGATTTGAGTAAG-AGCATGG-CTGTTGGGA-CCCGAAAGATGGTGAACTATGCTTGAATAGGGTGAAGCCAGAGG-AAACTCTGGTGGAGGCTCGCAGCGG--------------------------------------------------------------TTCTGAC-GTGCA--------------------------------------------------------------------------------AATCGATCGTCAA--------------ATTTGGGCATA-GGGGCGAAAGACTAATC----------------------------------------------------------------------------------------------------------GAACTAT--------------------------------CTAGTAGCTGGTTCCTGCCGAAGTTTCCCTCAGGATAGCAGTAACGTATTCAGTTTTATGAGGTAAAGCGAATGATTAGAGGCCTGGGGGTTGAAACAACCTTCACCTATTCTCAAACTTTAAATATGTAAGAAGTCC-TTGTTACTTAATTGAACGTGGACACTTGAATGTACCGTTACTAGTGGGCCATTTTTGGTAAGCAGAACTGGCGATGCGGGATGAACCGAACGCGGGGTTAAGGTGCCAGAATATACGCTCATCAGACACCACAAAAGGTGTTAGTTCATCTAGACAGCAGGACGGTGGCCATGGAAGTCGGAATCCGCTAAGGAGTGTGTAACAACTCACCTGCCGAATGAACTAGCCCTGAAAATGGATGGCGCTCAAGCGTGTTACCCATACCCCGCCGCCGGGGCAAGATTTAAGCCCCGGCGAGTAGGCAGGCGTGGAGGCT-------------------------------------------------

N.k.MH8747 -----------------------------------------------------------------------------------------------------------------------------------------------------------------------------------------------------------------------------------------------------------------------------------------------------------------------------------------------------------------------------------------------------------------------------------------------------------------------------------------------------------------------------------------------------------------------------------------------------------------------------------------------------------------------------------------------AC-----------------------------------------AGGG-ATTGCCCTAGTAAC--GGCGAGTGAAGCGGCATCAGCTCAAATTTGAAATCTGGCTCTTTCAGG---GTCCGAGTTGTAATTTGCAGAGGGCGCTTTGGCGTTGG-CAGCGGTCCAAGTTCCTTGGAACAGGACGTCACAGAGGGTGA-GAATCCCGTACGTGGTCGCT-GGCCTTCGCCGTGTAAAGCCCCTTCGACGAGTCGAGTTGTTTGGGAATGCAGCTCTAAATGGGAGGTAAATTTCTTCTAAAGCTAAATACTGGCCAGAGACCGATAGCGCACAAGTAGAGTGATCGAAAGATGAAAAGCACTTTG-GAAAGAGAGTCAAATAGCACGTGAAATTGTTGAAAGGGAAGCGCTTGCAGCCAGACTTGCCTGTAGTTGCTCATCCGGGCTTT--TGCCCGGTGCACTCTTCTGCGGGCAGGCCAGCATCAGTCTGGGCGGTTGGATAAAGGCCTCTGTCATGTACCTCCTCT---CGGGGAGGCCTTATAG-GGGAGGCGTAATGCAACCAGCCTGGACTGAGGTCCGCG-CA-TC--TGCTAGGATGCTGGCGTAATGGCTGTAAGCGGCCCGTCTTGAAACAC-GG--------------------------------------------ACCAAGGAGTCTAACATCTATGCGAGTGTTTGGGTGTCAAGCCCGAGCGCGTAA--TGAAAGTG-AACGGAGGTGGGAT-CCCTTTA-GGGTGCACCATCG-ACCGATCCTG--ATGTCTTCGGAT-GGATTTGAGTAAG-AGCATGG-CTGTTGGGA-CCCGAAAGATGGTGAACTATGCTTGAATAGGGTGAAGCCAGAGG-AAACTCTGGTGGAGGCTCGCAGCGG--------------------------------------------------------------TTCTGAC-GTGCA--------------------------------------------------------------------------------AATCGATCGTCAA--------------ATTTGGGCATA-GGGGCGAAAGACTAATC----------------------------------------------------------------------------------------------------------GAACTAT--------------------------------CTAGTAGCTGGTTCCTGCCGAAGTTTCCCTCAGGATAGCAGTAACGTATTCAGTTTTATGAGGTAAAGCGAATGATTAGAGGCCTGGGGGTTGAAACAACCTTCACCTATTCTCAAACTTTAAATATGTAAGAAGTCC-TTGTTACTTAATTGAACGTGGACACTTGAATGTACCGTTACTAGTGGGCCATTTTTGGTAAGCAGAACTGGCGATGCGGGATGAACCGAACGCGGGGTTAAGGTGCCAGAATATACGCTCATCAGACACCACAAAAGGTGTTAGTTCATCTAGACAGCAGGACGGTGGCCATGGAAGTCGGAATCCGCTAAGGAGTGTGTAACAACTCACCTGCCGAATGAACTAGCCCTGAAAATGGATGGCGCTCAAGCGTGTTACCCATACCCCGCCGCCGGGGCAAGATTTAAGCCCCGGCGAGTAGGCAGGCGTGGAGGCTCGTGACGAAGCCTTGG---------------------------------

Pa.u.isola --------------------------------------------------------------------------------------------------------------------------------------------------------------------------------------------------------------------------------------------------------------------------------------------------------------------------------------------------------------------------------------------------------------------------------------------------------------------------------------------------------------------------------------------------------------------------------------------------------------------------------------------------------------------------------------GAAA--CCAAC-----------------------------------------AGGG-ATTGCCCTAGTAAC--GGCGAGTGAAGCGGCATCAGCTCAAATTTGAAATCTGGCTCTTTTAGG---GTCCGAGTTGTAATTTGCAGAGGGCGCTTTGGCGTTGG-CAGCGGTCCAAGTTCCTTGGAACAGGACGTCACAGAGGGTGA-GAATCCCGTACGTGGTCGCT-GGCCTTCGCCGTGTAAAGCCCCTTCGACGAGTCGAGTTGTTTGGGAATGCAGCTCTAAATGGGAGGTAAATTTCTTCTAAAGCTAAATACTGGCCAGAGACCGATAGCGCACAAGTAGAGTGATCGAAAGATGAAAAGCACTTTG-GAAAGAGAGTCAAATAGCACGTGAAATTGTTGAAAGGGAAGCGCTTGCAGCCAGACTTGCCTGTAGTTGCTCATCCGGGCTTT--TGCCCGGTGCACTCTTCTGCGGGCAGGCCAGCATCAGTCTAGGCGGTTGGATAAAGGCCTCTGTCACGTACCTCCTTT---CGGGGAGGCCTTATAG-GGGAGGCGTAATGCAACCAGCCTGGACTGAGGTCCGCG-CA-TC--TGCTAGGATGCTGGCGTAATGGCTGTAAGCGGCCCGTCTTGAAACAC-GG--------------------------------------------ACCAAGGAGTCTAACATCTATGCGAGTGTTTGGGTGTCAAGCCCGAACGCGTAA--TGAAAGTG-AACGGAGGTGGGAT-CCCTTTA-GGGTGCACCATCG-ACCGATCCTG--ATGTCTTCGGAT-GGATTTGAGTAAG-AGCATGG-CTGTTGGGA-CCCGAAAGATGGTGAACTATGCTTGAATAGGGTGAAGCCAGAGG-AAACTCTGGTGGAGGCTCGCAGCGG--------------------------------------------------------------TTCTGAC-GTGCA--------------------------------------------------------------------------------AATCGATCGTCAA--------------ATTTGGGCATA-GGGGCGAAAGACTAATC----------------------------------------------------------------------------------------------------------GAA------------------------------------------------------------------------------------------------------------------------------------------------------------------------------------------------------------------------------------------------------------------------------------------------------------------------------------------------------------------------------------------------------------------------------------------------------------------------------------------------------------------------------------------------------------

Pa.te.MK35 ACGACCACCCCGAGCCGGAAAGTTGTCCAAACTCGGTCATTTAGAGGAAGTAAAAGTCGTAACAAGGTTTCCGTAGGTGAACCTGCGGAAGGATCATTACCATATCAAAACTGTCTGGGGGACGCACGCCGACGAGGTTTCACACAATGAAATGCGTCAGGATCCGTCCCTGTCTGGACCCTTGTTTTTTGCGTACTATTTGTTTCCTTGGTGGGCTTTGCCTGCCAAAAGGACACTATATCACCCTTTGTAATTGCAATCAGCGTCAGAAAAACATAATAGTTACAACTTTCAACAACGGATCTCTTGGTTCTGGCATCGATGAAGAACGCAGCGAAATGCGAAAAGTAGTGTGAATTGCAGAATTCAGTGAATCATCGAATCTTTGAACGCACATTGCGCCCCTTGGTATTCCATGGGGCATGCCTGTTCGAGCGTCATTTGTACCTTCAAGCCCTGCTTGGTGTTGGGTGTTTGTCCCCGTTTTACATGTGGGACTCGCCTTAAAGTGATTGGCAGCCGGCGTATAAGCCTTGGAGCGCAGCACATTTTGCGTCCCTCGGCCTGAACGTTGGCGTCCAGTAAGCCTATACTTTTGCTCTTGACCTCGGATCAGGTAGGGATACCCGCTGAACTTAAGCATATCAATAAGCGGAGGAAAAGAAA--CCAAC-----------------------------------------AGGG-ATTGCCCTAGTAAC--GGCGAGTGAAGCGGCATCAGCTCAAATTTGAAATCTGGCTCTTTTAGG---GTCCGAGTTGTAATTTGCAGAGGGCGCTTTGGCGTTGG-CAGCGGTCCAAGTTCCTTGGAACAGGACGTCACAGAGGGTGA-GAATCCCGTACGTGGTCGCT-GGCCTTCGCCGTGTAAAGCCCCTTCGACGAGTCGAGTTGTTTGGGAATGCAGCTCTAAATGGGAGGTAAATTTCTTCTAAAGCTAAATACTGGCCAGAGACCGATAGCGCACAAGTAGAGTGATCGAAAGATGAAAAGCACTTTG-GAAAGAGAGTCAAATAGCACGTGAAATTGTTGAAAGGGAAGCGCTTGCAGCCAGACTTGCCTGTAGTTGCTCATCCGGGCTTT--TGCCCGGTGCACTCTTCTGCGGGCAGGCCAGCATCAGTCTAGGCGGTTGGATAAAGGCCTCTGTCACGTACCTCCTTT---CGGGGAGGCCTTATAG-GGGAGGCGTAATGCAACCAGCCTGGACTGAGGTCCGCG-CA-TC--TGCTAGGATGCTGGCGTAATGGCTGTAAGCGGCCCGTCTTGAAACAC-GG--------------------------------------------ACCAAGGAGTCTAACATCTATGCGAGTGTTTGGGTGTCAAGCCCGAACGCGTAA--TGAAAGTG-AACGGAGGTGGGAT-CCCTTTA-GGGTGCACCATCG-ACCGATCCTG--ATGTCTTCGGAT-GGATTTGAGTAAG-AGCATGG-CTGTTGGGA-CCCGAAAGATGGTGAACTATGCTTGAATAGGGTGAAGCCAGAGG-AAACTCTGGTGGAGGCTCGCAGCGG--------------------------------------------------------------TTCTGAC-GTGCA--------------------------------------------------------------------------------AATCGATCGTCAA--------------ATTTGGGCATA-GGGGCGAAAGACTAATC----------------------------------------------------------------------------------------------------------GAACTAT--------------------------------CTAGTAGCTGGTTCCTGC------------------------------------------------------------------------------------------------------------------------------------------------------------------------------------------------------------------------------------------------------------------------------------------------------------------------------------------------------------------------------------------------------------------------------------------------------------------------------------------------------

Pl.am.AY78 -----------------------------------------------------------------------------------------------------------------------------------------------------------------------------------------------------------------------------------------------------------------------------------------------------------------------------------------------------------------------------------------------------------------------------------------------------------------------------------------------------------------------------------------------------------------------------------------------------------------------------------------------------------------------------------------A--CCAAC-----------------------------------------AGGG-ATTGCCTCAGTAAC--GGCGAGTGAAGCGGCATCAGCTCAAATTTGAAATCTGGCTCTTTTAGA---GTCCGAGTTGTAATTTGCAGAGGGCGCTTTGGCATAGG-CAGCGATTCAAGTTCCTTGGAACAGGACGTCACAGAGGGTGA-GAATCCCGTACGTGGTCGCT-AGCTCTTGCCGTGTAAAGCCCCTTCGACGAGTCGAGTTGTTTGGGAATGCAGCTCTAAATGGGAGGTAAATTTCTTCTAAAGCTAAATATTGGCCAGAGACCGATAGCGCACAAGTAGAGTGATCGAAAGATGAAAAAAACTTTG-GAAAGAGAGTTAAACAGCATGTGAAATTGTTGAAAGGGAAGCGCTTGCAGCCAGACTTGCCTGTAGTTGCTCATCCGGGCTCT--TGCCCGGTGCACTCTTCTGTAGGCAGGCCAGCATCAGTTTGGGCGGTTGGATAAAGGTCTCTGTCATGTACCTCTCTT---CGGGGAGGCCTTATAG-GGGAGGCGACATACAACCAGCCTAGACTGAGGTCCGCG-CA-TTCGTGCTAGGATGCTGGCGTAATGGCTGTAAGCGGCCCGTCTTGAAACAC-GG--------------------------------------------ACCAAGGAGTCTAACATCTATGCGAGTGTTTGGGTGTCAAGCCCGAGCGCGTAA--TGAAAGTG-AACGGAGGTGGGAA-CCCCTCG-GGGTGCACCATCG-ACCGATCCTG--ATGTCTTCGGAA-GGATTTGAGTAAG-AGCATGG-CTGTTGGGA-CCCGAAAGATGGTGAACTATGCTTGAATAGGGTGAAGCCAGAGG-AAACTCTGGTGGAGGCTCGCAGCGG--------------------------------------------------------------TTCTGAC-GTGCA--------------------------------------------------------------------------------AATCGATCGTCAA--------------ATTTGGGCATA-GGGGCGAAAGACTAATC----------------------------------------------------------------------------------------------------------GAACTAT--------------------------------CTAGTAGCTGGTTCCTGCCGA---------------------------------------------------------------------------------------------------------------------------------------------------------------------------------------------------------------------------------------------------------------------------------------------------------------------------------------------------------------------------------------------------------------------------------------------------------------------------------------------------

Al.al.DQ67 --------------------------------------------------------------------------------------------------------------------------------------------------------------------------------------------------------------------------------------------------------------------------------------------------------------------------------------------------------------------------------------------------------------------------------------------------------------------------------------------------------------------------------------------------------------------------------------------------------------------------------------------------------------------------AGCGGAGGAAAAGAAA--CCANC-----------------------------------------AGGG-ATTGCCCTAGTAAC--GGCGAGTGAAGCGGCAACAGCTCAAATTTGAAATCTGGCTCTTTTAGA---GTCCGAGTTGTAATTTGCAGAGGGCGCTTTGGCTTTGG-CAGCGGTCCAAGTTCCTTGGAACAGGACGTCACAGAGGGTGA-GAATCCCGTACGTGGTCGCT-GGCTATTGCCGTGTAAAGCCCCTTCGACGAGTCGAGTTGTTTGGGAATGCAGCTCTAAATGGGAGGTACATTTCTTCTAAAGCTAAATATTGGCCAGAGACCGATAGCGCACAAGTAGAGTGATCGAAAGATGAAAAGCACTTTG-GAAAGAGAGTCAAACAGCACGTGAAATTGTTGAAAGGGAAGCGCTTGCAGCCAGACTTGCTTACAGTTGCTCATCCGGGTTTC--TACCCGGTGCACTCTTCTGTAGGCAGGCCAGCATCAGTTTGGGCGGTAGGATAAAGGTCTCTGTCACGTACCTCCTTT---CGGGGAGGCCTTATAG-GGGAGACGACATACTACCAGCCTGGACTGAGGTCCGCG-CA-TC--TGCTAGGATGCTGGCGTAATGGCTGTAAGCGGCCCGTCTTGAAACAC-GG--------------------------------------------ACCAAGGAGTCTAACATCTATGCGAGTGTTTGGGTGTCAAGCCCGAGCGCGTAA--TGAAAGTG-AACGGAGGTGGGAA-CCCGCAA-GGGTGCACCATCG-ACCGATCCTG--ATGTCTTCGGAA-GGATTTGAGTAAG-AGCATGG-CTGTTGGGA-CCCGAAAGATGGTGAACTATGCTTGAATAGGGTGAAGCCAGAGG-AAACTCTGGTGGAGGCTCGCAGCGG--------------------------------------------------------------TTCTGAC-GTGCA--------------------------------------------------------------------------------AATCGATCGTCAA--------------ATTTGGGCATA-GGGGCGAAAGACTAATC----------------------------------------------------------------------------------------------------------GAACTAT--------------------------------CTAGTAGCTGGTTCCTGCCGAAGTTTCCCTCAGGATAGCAGTAACGTATTCAGTTTTATGAGGTAAAGCGAATGATTAGAGGCCTGGGGGTTGAAACAACCTTCACCTATTCTCAAACTTTAAATATGTAAGAAGTCC-TTGTTACTTAATTGAACGTGGACAGTTGAATGAAACGTTATTAGTGGGCCATTTTTGGTAAGCAGAACTGGCGATGCGGGATGAACCGAACGAGGGGTTAAAGTGCCGGAATATACGCTCATCAGACACCACAAAAGGTGTTGGTTCATCTAGACAGCAGGACGGTGGCCATGGAAGTCGGAATCCGCTAAGGAGTGTGTAACAACTCACCTGCCGAATGAACTAGCCCTGAAAATGGATGGCGCTCAAGCGTGTTACTTATACCCCTCCGCTGGGGCAAAATTTACGCCCCAGCGAGTAGGCAGGCGTGGAGGTCCGTGACGAA----------------------------------------

Pl.he.DQ67 ----------------------------------------------------------------------------------------------------------------------------------------------------------------------------------------------------------------------------------------------------------------------------------------------------------------------------------------------------------------------------------------------------------------------------------------------------------------------------------------------------------------------------------------------------------------------------------------------------------------------------------------------------------------------------------------------------------------------------------------------------------------------------GCAACAGCTCAAATTTGAAATCTGGCTCTTTTAGG---GTCCGAGTTGTAATTTGCAGAGGGCGCTTTGGCTTTGG-CAGCGGTCCAAGTTCCTTGGAACAGGACGTCACAGAGGGTGA-GAATCCCGTACGTGGTCGCT-AGCTATTGCCGTGTAAAGCCCCTTCGACGAGTCGAGTTGTTTGGGAATGCAGCTCTAAATGGGAGGTAAATTTCTTCTAAAGCTAAATATTGGCCAGAGACCGATAGCGCACAAGTAGAGTGATCGAAAGATGAAAAGCACTTTG-GAAAGAGAGTCAAACAGCACGTGAAATTGTTGAAAGGGAAGCGCTTGCAGCCAGACTTGCTTGCAGTTGCTCATCCGGGCTTT--TGCCCGGTGCACTCTTCTGTAGGCAGGCCAGCATCAGTTTGGGCGGTGGGATAAAGGTCTCTGTCACGTACCTCTCTT---CGGGGAGGCCTTATAG-GGGAGACGACATACCACCAGCCTAGACTGAGGTCCGCG-CA-TC--TGCTAGGATGCTGGCGTAATGGCTGTAAGCGGCCCGTCTTGAAACAC-GG--------------------------------------------ACCAAGGAGTCTAACATCTATGCGAGTGTTTGGGTGTCAAGCCCGAGCGCGTAA--TGAAAGTG-AACGGAGGTGGGAA-CCCGCAA-GGGTGCACCATCG-ACCGATCCTG--AAGTTTTCGGAA-GGATTTGAGTAAG-AGCATGG-CTGTTGGGA-CCCGAAAGATGGTGAACTATGCTTGAATAGGGTGAAGCCAGAGG-AAACTCTGGTGGAGGCTCGCAGCGG--------------------------------------------------------------TTCTGAC-GTGCA--------------------------------------------------------------------------------AATCGATCGTCAA--------------ATTTGGGCATA-GGGGCGAAAGACTAATC----------------------------------------------------------------------------------------------------------GAACTAT--------------------------------CTAGTAGCTGGTTCCTGCCGAAGTTTCCCTCAGGATAGCAGTAACGTATTCAGTTTTATGAGGTAAAGCGAATGATTAGAGGCATGGGGGTTGAAACAACCTTCACCTATTCTCAAACTTTAAATATGTAAGAAGCCCTTTGTTACTTGATTGAACGCGGGCATTTGAATGAAACGTTATTAGTGGGCCATTTTTGGTAAGCAGAACTGGCGATGCGGGATGAACCGAACGAGGGGTTACGGTGCCGGAGTACACGCTCATCAGACACCACAAAAGGTGTTGGTTCATCTAGACAGCAGGACGGTGGCCATGGAAGTCGGAATCCGCTAAGGAGTGTGTAACAACTCACCTGCCGAATGAACTAGCCCTGAAAATGGATGGCGCTCAAGCGTGTCACCTATACCCCTCCGCCGGGGCAAAATTTACGCCCCG------------------------------------------------------------------------

Pyp.tr.rep ---------------------------------------------------------------------------------------------------------------------------------------------------------------------------------------------------------------------------------------------------------------------------------------------------------------------------------------------------------------------------------------------------------------------------------------------------------------------------------------------------------------------------------------------------------------------------------------------------------------------------------------------------------------------------------GGAAAGAAA--CCAAC-----------------------------------------AGGG-ATTGCCCTAGTAAC--GGCGAGTGAAGCGGCAACAGCTCAAATTTGAAATCTGGCTCTTTTAGA---GTCCGAGTTGTAATTTGCAGAGGGCGCTTTGGCTTTGG-CAGCGGTCCAAGTTCCTTGGAACAGGACGTCACAGAGGGTGA-GAATCCCGTACGTGGTCGCT-AGCTATTGCCGTGTAAAGCCCCTTCGACGAGTCGAGTTGTTTGGGAATGCAGCTCTAAATGGGAGGTAAATTTCTTCTAAAGCTAAATACTGGCCAGAGACCGATAGCGCACAAGTAGAGTGATCGAAAGATGAAAAGCACTTTG-GAAAGAGAGTCAAACAGCACGTGAAATTGTTGAAAGGGAAGCGCTTGCAGCCAGACTTGCTTGCAGTTGCTCACCCGGGCCTCTGTGCCCGGTGCATTCTTCTGCAGGCAGGCCAGCATCAGTTTGGGCGGTGGGATAAAGGTCTCTGTCACGTACCTCTCTT---CGGGGAGGCCTTATAG-GGGAGGCGACATACCACCAGCCTGGACTGAGGTCCGCG-CA-TTTATGCTAGGATGCTGGCGTAATGGCTGTAAGCGGCCCGTCTTGAAACAC-GG--------------------------------------------ACCAAGGAGTCTAACATCTATGCGAGTGTTTGGGTGTCAAGCCCGAACGCGTAA--TGAAAGTG-AACGGAGGTGGGAA-CCCGCAA-GGGTGCACCATCG-ACCGATCCTG--ATGTCTTCGGAA-GGATTTGAGTAAG-AGCATGG-CTGTTGGGA-CCCGAAAGATGGTGAACTATGCTTGAATANGGTGAAGCCAGAGG-AAACTCTGGTGGAGGCTCGCAGCGG--------------------------------------------------------------TTCTGAC-GTGCA--------------------------------------------------------------------------------AATCGATCGTCAA--------------ATTTGGGCATA-GGGGCGAAAGACTAATC----------------------------------------------------------------------------------------------------------GAACTAT--------------------------------CTAGTAGCTGGTTCCTGCCGAAGTTTCCCTCAGGATAGCAGTAACGTATTCAGTTTTATGAGGTAAAGCGAATGATTAGAGGCCTGGGGGTTGAAACAACCTTCACCTATTCTCAAACTTTAAATATGTAAGAAGTGT-TTGTTGCTTAATTGAACGTACACATTTGAATGAAACGTTATTAGTGGGCCATTTTTGGTAAGCAGAACTGGCGATGCGGGATGAACCGAACGAGGGGTTAAAGTGCCGGAATATACGCTCATCAGACACCACAAAAGGTGTTGGTTCATCTAGACAGCAGGACGGTGGCCATGGAAGTCGGAATCCGCTAAGGAGTGTGTAACAACTCACCTGCCGAATGAACTAGCCCTGAAAATGGATGGCGCTCAAGCGTGTTACTTATACCCCTCCGCCGGGGCAAAATTTACGCCCCGGCGAGTAGGCAGGCGTGGAGGCTCGTGACGAAGCCCTAGGGGTGACCCT-----------------------

Pyp.phe.DQ ------------------------------------------------------------------------------------------------------------------------------------------------------------------------------------------------------------------------------------------------------------------------------------------------------------------------------------------------------------------------------------------------------------------------------------------------------------------------------------------------------------------------------------------------------------------------------------------------------------------------------------------------------------TAAGCATATCAATAAGCGGAGGAAAAGAAA--CCAAC-----------------------------------------AGGG-ATTGCCCTAGTAAC--GGCGAGTGAAGCGGCAACAGCTCAAATTTGAAATCTGGCTCTTTTAGG---GTCCGAGTTGTAATTTGCAGAGGGCGCTTTGGCTTTGG-CAGCGGTCCAAGTTCCTTGGAACAGGACGTCACAGAGGGTGA-GAATCCCGTACGTGGTCGCT-AGCCATTGCCGTGTAAAGCCCCTTCGACGAGTCGAGTTGTTTGGGAATGCAGCTCTAAATGGGAGGTAAATTTCTTCTAAAGCTAAATATTGGCCAGAGACCGATAGCGCACAAGTAGAGTGATCGAAAGATGAAAAGCACTTTG-GAAAGAGAGTCAAACAGCACGTGAAATTGTTGAAAGGGAAGCGCTTGCAGCCAGACTTGCTTGCAGTTGCTCATCCGGGCTTT--TGCCCGGTGCACTCTTCTGCAGGCAGGCCAGCATCAGTTTGGGCGGTGGGATAAAGGTCTCTGTCACGTACCTCCTTT---CGGGGAGGCCTTATAG-GGGAGGCGACATACCACCAGCCTGGACTGAGGTCCGCG-CA-TT--CGCTAGGATGCTGGCGTAATGGCTGTAAGCGGCCCGTCTTGAAACACGGG--------------------------------------------ACCAAGGAGTCTAACATCTATGCGAGTGTTTGGGTGTCAAGCCCGAACGCGTAA--TGAAAGTG-AACGGAGGTGGGAA-CCCGCAA-GGGTGCACCATCG-ACCGATCCTG--ATGTCTTCGGAA-GGATTTGAGTAAG-AGCATGG-CTGTTGGGA-CCCGAAAGATGGTGAACTATGCTTGAATAGGGTGAAGCCAGAGG-AAACTCTGGTGGAGGCTCGCAGCGG--------------------------------------------------------------TTCTGAC-GTGCA--------------------------------------------------------------------------------AATCGATCGTCAA--------------ATTTGGGCATA-GGGGCGAAAGACTAATC----------------------------------------------------------------------------------------------------------GAACTAT--------------------------------CTAGTAGCTGGTTCCTGCCGAAGTTTCCCTCAGGATAGCAGTAACGTATTCAGTTTTATGAGGTAAAGCGAATGATTAGAGGCCTGGGGGTTGAAACAACCTTCACCTATTCTCAAACTTTAAATATGTAAGAAGTGC-TTGTTGCTTAATTGAACGTGCACATTTGAATGAAACGTTATTAGTGGGCCATTTTTGGTAAGCAGAACTGGCGATGCGGGATGAACCGAACGAGGGGTTAAGGTGCCGGAATATACGCTCATCAGACACCACAAAAGGTGTTGGTTCATCTAGACAGCAGGACGGTGGCCATGGAAGTCGGAATCCGCTAAGGAGTGTGTAACAACTCACCTGCCGAATGAACTAGCCCTGAAAATGGATGGCGCTCAAGCGTGTTACCTATACCCCTCCGCCGGGGCAGAATTTACGCCCCGGCGAGTAGGCAGGCGTGGAGGCTCGTGACGAAGCCCTAGGG-------------------------------

;

END;

**Tree**

(((((((((((Neocucurbitaria_keratinophila_MH874704.1:0.01126,

Pyrenochaeta_nobilis_DQ678096.1:0)0.81:0.01125,

(Parafenestella_tetratrupha_MK356319.1:0,

Parafenestella_ulmi_isolate_OL897166.1:0)0:0)0.79:0.01132,

(Podonectria_novae-zelandiae_MW462902.1:0,

Podonectria_novae-zelandiae_MW462903.1:0)0.84:0.01137)0:0,

((Pleospora_ambigua_AY787937:0.01058,Coniothyrium_palmarum_DQ767653.1:0.02343)

0.77:0.01106,((Pyrenophora_tritici-repentis_AY544672.1:0,

(Pyrenophora_phaeocomes_DQ499596.1:0.01189,

Alternaria_alternata_DQ678082.1:0.01097)0.89:0.02323)0:0,

Pleospora_herbarum_DQ678049.1:0.0111)0.67:0.01235)0.94:0.03533)0.74:0.02011,

(Helminthosporium_jiulianshanense_OQ172253.1:0,

Helminthosporium_chengduense_ON557745.1:0)0.79:0.02739)0.47:0.02554,

Helminthosporium_meilingense_OQ172238.1:0.02872)0:0,

Pleospora_iqbalii_NG_057738.1:0.07366)0.78:0.04717,

(Lophiostoma_arundinis_DQ782384.1:0,Lophiostoma_crenatum_DQ678069.1:0)

0.98:0.1595)0.89:0.0756,(((Macrophomina_phaseolina_DQ678088.1:0,

((Botryosphaeria_dothidea_DQ678051.1:0,Botryosphaeria_sinensis_OQ845867:0)

0.87:0.02424,Botryosphaeria_viticola_DQ678087.1:0.01116)0.4:0.01249)0.86:0.04816,

Guignardia_bidwellii_DQ678085.1:0.07321)0.86:0.03698,

((Acanthostigma_scopulum_GQ850489.1:0.01735,Tubeufia_paludosa_AY849966.1:0.04554)

0.75:0.03486,Tubeufia_cerea_DQ470982.1:0.05754)0.82:0.03868)0.77:0.01342)0:0,

((((Elsinoe_centrolobi_DQ678094.1:0,Elsinoe_phaseoli_DQ678095.1:0.0241)

0.9:0.04487,Myriangium_duriaei_DQ678059.1:0.1308)0.45:0.01661,

(((Dothidea_hippophaeos_DQ678048.1:0.01172,Dothidea_sambuci_AY544681.1:0.01183)

0:0,Dothidea_insculpta_DQ247802.1:0.02402)0.76:0.03166,

Dothiora_cannabinae_DQ470984.1:0.0097)0.81:0.02865)0.84:0.02854,

(((((MN032446.1_Ellismarsporium_parvum_isolate_INIFAT2483_large_subun:0,

Ellismarsporium_parvum_MN032445.1:0)0.84:0.02828,

(Kirschsteiniothelia_aethiops_MH872853.1:0,

Kirschsteiniothelia_aethiops_MH877780.1:0)0.98:0.07066)0:0.00318,

Helminthosporium_asterinum_MH867156:0.0437)0.9:0.06493,

((Malacaria_flagellata_AK4H_Coffea_arabica:0,

(Atractilina_parasitica_MB136_Clerodendrum_capitatum:0,

(Malacaria_flagellata_AK06H_Coffea_arabica:0,

(Atractilina_parasitica_MB178a_Coffea_arabica:0,

Atractilina_parasitica_AK06h_Coffea_arabica:0)0:0)0:0)0:0)0:0,

Atractilina_parasitica_MB178b_Coffea_arabica:0.01185)0.99:0.1462)0.69:0.01395,

(Hysterobrevium_mori_FJ161196:0,Hysterobrevium_mori_FJ161202:0)0.96:0.08933)

0.73:0.0143)0:0)0.95:0.03087,((Capnodium_coffeae_DQ247800.1:0.05957,

Capnodium_salicinum_DQ678050.1:0.05118)0.88:0.05945,

(Cladosporium_cladosporioides_isolate_DQ678057.1:0.2407,

(Mycosphaerella_punctiformis_DQ470968.1:0,

(Cercospora_beticola_DQ678091.1:0.03995,

Mycosphaerella_fijiensis_DQ678098.1:0.0486)0.94:0.05822)0.94:0.109)0:0)

0.95:0.04142);
